# Ventricular CSF proteomic profiles and predictors of surgical treatment outcome in chronic hydrocephalus

**DOI:** 10.1007/s00701-023-05832-y

**Published:** 2023-10-19

**Authors:** Nina Rostgaard, Markus Harboe Olsen, Sara Diana Lolansen, Nicolas Hernandez Nørager, Peter Plomgaard, Nanna MacAulay, Marianne Juhler

**Affiliations:** 1 Department of Neurosurgery, The Neuroscience Centre, Copenhagen University Hospital, Rigshospitalet, Copenhagen, Denmark; 2 Department of Neuroanaesthesiology, The Neuroscience Centre, Copenhagen University Hospital, Rigshospitalet, Copenhagen, Denmark; 3 Department of Neuroscience, Faculty of Health and Medical Sciences, University of Copenhagen, Copenhagen, Denmark; 4 Department of Clinical Biochemistry, Centre of Diagnostic Investigations, Copenhagen University Hospital, Rigshospitalet, Copenhagen, Denmark; 5 Department of Clinical Medicine, Faculty of Health and Medical Sciences, University of Copenhagen, Copenhagen, Denmark

**Keywords:** Obstructive hydrocephalus, Idiopathic normal pressure hydrocephalus, Cerebrospinal fluid, Biomarkers, Proteomic profile, Predictors, Mass spectrometry, Proteomics

## Abstract

**Background:**

By applying an unbiased proteomic approach, we aimed to search for cerebrospinal fluid (CSF) protein biomarkers distinguishing between obstructive and communicating hydrocephalus in order to improve appropriate surgical selection for endoscopic third ventriculostomy vs. shunt implants. Our second study purpose was to look for potential CSF biomarkers distinguishing between patients with adult chronic hydrocephalus benefitting from surgery (responders) vs. those who did not (non-responders).

**Methods:**

Ventricular CSF samples were collected from 62 patients with communicating hydrocephalus and 28 patients with obstructive hydrocephalus. CSF was collected in relation to the patients’ surgical treatment. As a control group, CSF was collected from ten patients with unruptured aneurysm undergoing preventive surgery (vascular clipping).

**Results:**

Mass spectrometry-based proteomic analysis of the samples identified 1251 unique proteins. No proteins differed significantly between the communicating hydrocephalus group and the obstructive hydrocephalus group. Four proteins were found to be significantly less abundant in CSF from communicating hydrocephalus patients compared to control subjects. A PCA plot revealed similar proteomic CSF profiles of obstructive and communicating hydrocephalus and control samples. For obstructive hydrocephalus, ten proteins were found to predict responders from non-responders.

**Conclusion:**

Here, we show that the proteomic profile of ventricular CSF from patients with hydrocephalus differs slightly from control subjects. Furthermore, we find ten predictors of response to surgical outcome (endoscopic third ventriculostomy or ventriculo-peritoneal shunt) in patients with obstructive hydrocephalus.

**Supplementary Information:**

The online version contains supplementary material available at 10.1007/s00701-023-05832-y.

## Introduction

Classification of chronic hydrocephalus is based on the cerebrospinal fluid (CSF) drainage routes. Overall, the route can be either obstructed – blocking the CSF flow – or communicating, where the CSF passages appear clear [35, 36, 41, 44, 51, 64]. Regardless of classification, the treatment of chronic hydrocephalus is almost exclusively surgical by either implantation of a mechanically operated drainage system (shunt system) or endoscopic fenestration of the ventricular system [1, 19]. Implanted shunt systems can be used regardless of individual hydrocephalus characteristics; however, functional limitations and complications affect the durability of these systems, and many publications report limited survival of hydrocephalus shunts [26, 27, 37, 39, 49, 57, 59, 65].

Endoscopic hydrocephalus procedures – e.g., endoscopic third ventriculostomy (ETV) – are usually performed through the 3rd ventricle floor and less frequently by opening an obstructed aqueduct [71]. These procedures have the advantage of being implant-free and thereby make use of the normal physiological CSF clearance. The long-term durability is also suggested favorable compared to implanted shunts [23], with the evidence mostly based on data in pediatric hydrocephalus [9, 23, 32, 38, 53]. However, endoscopic surgery is only successful if the endoscopic fenestration bypasses or eliminates an obstruction in the CSF pathways [1, 8, 67]. Thus, identification of CSF obstruction is essential for selecting endoscopic hydrocephalus surgery. This is relatively simple to determine when there are obvious anatomic obstructions visible in imaging studies, e.g., aqueductal stenosis [35, 48, 62, 71]. However, it is not always possible to distinguish between patients who present such CSF obstructions and those who do not. In such cases, a possible obstruction site with the potential to treat endoscopically is only inferred from imaging studies [18, 33, 58]. Furthermore, good outcomes following ETV for hydrocephalus with communicating CSF pathways, such as normal pressure hydrocephalus (NPH), have been reported [11, 17, 61, 66]. In those cases, sub-structural obstacles to CSF flow might be invisible with current imaging technology. Therefore, it would be useful for the surgical decision between shunt implantation and endoscopic surgery to have a CSF biomarker which could (1) support the distinction between obstructive and non-obstructive hydrocephalus and thus further support the surgical decision between shunt implantation and ETV and (2) be used as a pre-surgical predictor for treatment effect.

Prediction of response to surgical treatment for chronic hydrocephalus is a clinical challenge, particularly in communicating cases like NPH. In NPH, treatment decision is based on a combination of symptoms (“Hakim’s triad”), radiological features, and, in many institutions, additional CSF dynamic studies or clinical testing before and after tapping of CSF. With application of strict selection criteria, the response rate is 75–80%. However, as many as 30% of patients not fulfilling these strict selection criteria might have benefitted from surgery [10, 17, 70]. Criteria to predict the statistical success rate for ETV have been published and are widely used for children, but currently, no similar prediction scores exist for ETV in adults [31, 62]. An inappropriate surgical decision may result in redundant procedures, e.g., further surgery in order to treat the hydrocephalus condition, further neurological work-up to determine possible pharmacological treatment, or finally acceptance of a non-treatable condition. A pre-surgical biomarker improving the selection of patients for surgery is thus greatly needed, particularly in this group of patients.

Accordingly, the main purpose of this study is to reveal CSF biomarkers distinguishing between obstructive and communicating hydrocephalus by determining  the proteomic profile of pre-surgically collected CSF and comparing this profile in cases with definite or probable CSF pathway obstruction (patients with obstructive hydrocephalus) against cases without such obstructions (patients with communicating hydrocephalus). Our second study purpose was to look for potential biomarkers distinguishing between patients with adult chronic hydrocephalus benefitting from surgery (either ventriculo-peritoneal (VP) shunt or ETV; responders) vs. those who did not (non-responders) by comparison of the proteomic profiles in the responder and non-responder group.

## Materials and methods

### Patients and sample collection

This study included ventricular CSF samples extracted from 62 patients with communicating hydrocephalus (mean age: 75 years, range: 54–87 years, 23F/39 M), where CSF was collected upon shunt surgery, taptest through shunt chamber (*n* = 1) or insertion of the extra-ventricular drain (EVD) (*n* = 1). All of the patients with communicating hydrocephalus were diagnosed with NPH as adults without suspicion of the condition being congenital. CSF was sampled from 28 patients with obstructive hydrocephalus (mean age: 59 years, range: 24–75 years, 15F/13 M), where CSF was collected upon ETV (Table 1). Of the patients with obstructive hydrocephalus, 14 were diagnosed as adults without suspicion of the condition being congenital, 13 were diagnosed as adults with suspicion of the condition being congenital, and one was diagnosed as a child with the condition being congenital. All CSF samples were collected directly in connection with the surgical procedure according to standard surgical procedures at the Department of Neurosurgery at Rigshospitalet, Copenhagen, Denmark. The CSF samples were kept on ice and centrifuged at 2000 × *g* for 10 min at 4 °C within 2 h from the collection before being aliquoted in polypropylene microtubes (Sarstedt, Germany) and subsequently stored at − 80 °C [7]. Patients receiving a VP shunt or undergoing ETV were divided into groups on the basis of their response to surgical treatment. The assessment/scoring was carried out by an experienced neurosurgeon (MJ). As the control group, ten patients with unruptured aneurism undergoing preventive surgery (vascular clipping) were enrolled (mean age: 56 years, range: 39–71 years, 6F/4 M), and the CSF was collected from the basal cisterns during surgery prior to the clipping of the aneurism. Written informed consent was obtained from all patients and control subjects, and the study was approved by the Danish National Committee on Health Research Ethics (approval no. H-19001474 and H-17011472) and the Danish Data Protection Agency (VD-2019–210).

**Table 1:** Clinical characteristics of the study cohort

Study cohort	Communicating HC	Obstructive HC	Control subjects (cold aneurysms)
*N*	62	28	10
Age (years), median (range)	76 (54–87)	63 (24–75)	56 (39–71)
Sex (F/M)	23/39	15/13	6/4
Responders	44	20	NA
Non-responders	16	8	NA
Response rate % (responders/no. of treated)	71 (44/62)	71 (20/28)	NA

*N*, number of included individuals; *F*, female; *M*, male; *HC*, hydrocephalus

### Protein digestion and Evotips loading

Human CSF sample preparation was performed on an Agilent Bravo Liquid Handling Platform (Agilent, CA, USA) according to an optimized version of previously published protocols [2, 12]. Briefly, CSF samples were aliquoted into a 96-well format plate and introduced to the Bravo Robot (Agilent, CA, USA). 20 µl CSF sample was mixed with 30 µl PreOmics Lysis buffer (P.O. 00001, PreOmics GmbH, Germany) and incubated at 95 °C for 10 min in order to denature proteins and reduce disulfide bridges and alkylate cysteines [30]. After cooling the sample for 15 min at room temperature, trypsin and LysC (0.5 µg/ul, Promega, WI, USA) were added in a ratio of 1 µg enzyme to 100 µg proteins and the mixture incubated at 37 °C for 4 h. The peptide mixtures were diluted in 100 µl 99% isopropanol and 1% Trifluoro-acetic acid (TFA) and desalted using two-gauge reversed-phase styrenedivinylbenzene (SDB-RPS) stage-tips. Afterwards, the stage-tips were washed using 200 µl 99% isopropanol and 1% TFA, followed by 200 µl 0.2% TFA. The purified peptides were eluted using 80% acetonitrile (VWR chemicals, PA, USA) containing 1% ammonia (Merck, Germany) and subsequently dried down. Peptides were resuspended in solvent A (0.1% formic acid (FA) in water) and loaded onto Evotips (Evosep Biosystem, Denmark) according to the manufacturer’s recommendations. The Evotips were wetted with isopropanol for 5 min, activated with 20 µl solvent B (99% CAN, 0.1% FA) and centrifuged at 700 − *g* for 1 min. 20 µl of solution A was then added to equilibrate the tips, followed by sample loading. Finally, 20 µl buffer A was used to wash the Evotip, and 100 µl was added to avoid drying.

### Liquid chromatography and mass spectrometry (MS) analysis

The samples were injected into an Exploris 480 Thermo Fischer Scientific system using Evosep One (Evosep Biosystem, Denmark). A preset chromatographic method was used, corresponding to 60 samples per day**.** The peptides were separated on an 8 cm Pepsep column (150 μm, ID 1.5 μm bead size Reprosil-Pur C18 beads, Denmark) at 1 μl/min flow rate with a 21-min gradient. The heated capillary temperature was set to 275 °C, the spray voltage to 2650 V, and the funnel radiofrequency to 40 Hz. The mass spectrometer was operated in a data-independent mode (DIA) with a full MS range from 350 to 1650 m/z at a resolution of 60,000 at 200 m/z. The AGC target was set to 300% with an injection time of 50 ms. The AGC value of the targeted MS2 experiment was set to 1000%. Thirty-two windows of variable sizes were defined for target MS2 (tMS2) acquisition and subjected to high-energy collisional dissociation (HCD) fragmentation with a normalized collision energy of 30%. Each tMS2 scan was acquired at a resolution of 30,000 with a maximum ion injection time (IT) of 100 ms for a scan range of m/z 349.5 to 1650.5.

### Data handling

The MS raw files were processed with Spectronaut version 15 (Biognosys, Switzerland). A previously generated CSF spectral library was imported from MaxQuant software analyses. The library contained 2733 protein groups and 17,301 peptides. DIA files were searched against the library using default parameters except for the normalization, which was set to local. Dynamic mass and retention time tolerances (for both MS1 and MS2) were applied. The Q-value cut-off was set to 1% both at precursor and protein levels using a mutated decoy method [5]. The calibration was performed based on a local regression model [6]. Protein data was exported from Spectronaut and further processed using the clinical knowledge graph (CKG) [56], together with their matching experimental and clinical data. Intensities were log-transformed before further statistical analysis.

### Statistical analysis

Statistical analyses were carried out using R v. 4.2.1 (R Core Team, Austria). Continuous data were presented as mean and standard deviation (SD) or median and interquartile range (IQR)/range depending on normality, while categorical data were presented as *n*, proportion, and percentage. Proteomics data were assessed for availability in CSF from all patient groups to assess which proteins are available (above level of detection). A principal component analysis (PCA) plot with complete case analysis (those proteins available in all samples) [24]. PCA addresses if the patterns in the groups differ. Protein abundances were considered significantly higher or lower in one group if the difference in the Bonferroni-corrected *P* value was < 0.05 (corresponding to *P* values below 0.0001) and there was a two-fold change between the groups. Only proteins available in at least 5 healthy controls and 10 patients of either hydrocephalus group were assessed. The lack of blood contamination of the CSF samples was verified by quantification of blood proteins (Supplementary information 1).

## Results

To determine the proteomic profile of obstructive and communicating hydrocephalus, we analyzed ventricular CSF by MS-based proteomics. In total, 1251 unique proteins were identified. Of those, 640 were detected in at least 5 control samples and 10 communicating hydrocephalus samples, and 616 were detected in at least 5 control samples and 10 obstructive hydrocephalus samples.

### Identifying biomarkers for communicating vs. obstructive hydrocephalus

**Fig. 1:**
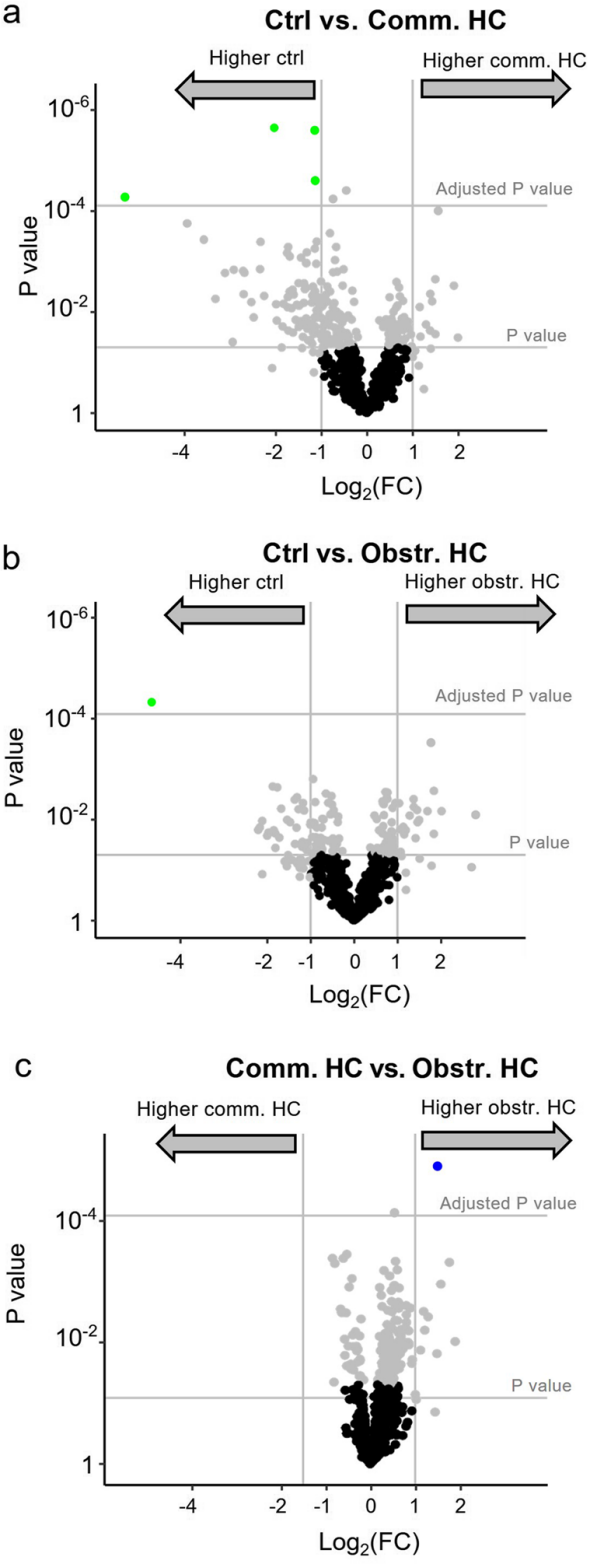
Volcano plots of proteomic data obtained from ventricular CSF from hydrocephalus patients. Comparisons of proteomic data in CSF from (**a**) control subjects and patients with communicating hydrocephalus (*n* = 640 proteins), (**b**) control subjects and patients with obstructive hydrocephalus (*n* = 616 proteins), and (**c**) patients with communicating hydrocephalus and patients with obstructive hydrocephalus (*n* = 619 proteins). The threshold of percentage change was set to a two-fold increase or decrease compared to either control CSF or obstructive hydrocephalus (marked with vertical lines) and *P* value after Bonferroni correction. Gray dots represent proteins which show a *tendency* to be increased in either of the groups by meeting the threshold of twofold change or the unadjusted *P* value but did not reach the significance of the Bonferroni-corrected *P* value. Black dots represent proteins found in *similar* abundance in the two groups. Ctrl: control subjects; Comm.: communicating; Obstr.: obstructive; HC: hydrocephalus

**Fig. 2:**
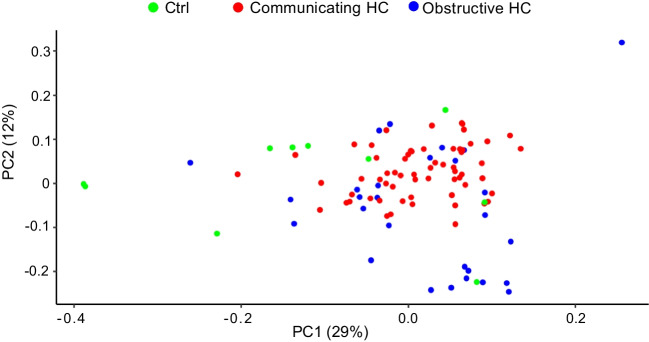
A PCA plot of CSF samples from control subjects and patients with communicating or obstructive hydrocephalus. The plot shows no clear clustering of proteomic profiles of different types of hydrocephalus and controls. Patients with communicating hydrocephalus: *n* = 62; patients with obstructive hydrocephalus: *n* = 28; control subjects: *n* = 10; ctrl: controls; PCA: principal component analysis; HC: hydrocephalus

To determine if CSF from patients with communicating hydrocephalus, obstructive hydrocephalus and control subjects showed a distinct proteomic profile; data were plotted as a volcano plot to visualize the profiles of the groups (Fig. 1). When CSF from communicating hydrocephalus patients was compared to CSF from control subjects, four proteins, vimentin (VIM), protocadherin alpha subfamily C2 (PCDHAC2), glutathione synthetase (GSS), and prolyl 4-hydroxylase subunit beta (P4HB), were found in significantly higher abundance in control subjects (Fig. 1a and Table 2). When comparing obstructive hydrocephalus to controls, only vimentin (VIM) was found to show a higher abundance in controls (Fig. 1b and Table 3). When the obstructive hydrocephalus group was compared to the communicating hydrocephalus group, we found one protein, syndecan binding protein (SDCBP), to differ significantly, with higher abundance in obstructive hydrocephalus (Fig. 1c and Table 4). In accordance with these few differences in the proteomic group profiles, the PCA plot did not reveal distinct proteomic distributions of either obstructive hydrocephalus, communicating hydrocephalus, or control samples (Fig. 2). All proteins detected by the MS-based analysis, which were included in the three comparisons in Fig. 1, are listed in Supplementary information 2, 3, and 4.

**Table 2:** Proteins found in significantly higher abundance in controls compared to communicating HC

Protein name	Uniprot ID	Ctrl subjects, mean (SD) [*N*]	Comm. HC, mean (SD) [*N*]	*P* value	*P* adjusted	Fold change	Log_2_ (fold change)
VIM	P08670	18.2 (1.8) [8]	12.8 (1.0) [49]	< 0.001	0.034	0.03	− 5.06
PCDHAC2	Q9Y5I4	14.6 (0.3) [6]	13.4 (0.9) [50]	< 0.001	0.002	0.45	− 1.15
GSS	P48637	15.6 (0.4) [5]	13.6 (0.9) [20]	< 0.001	0.001	0.24	− 2.06
P4HB	P07237	13.4 (0.4) [7]	12.3 (0.8) [21]	< 0.001	0.016	0.46	− 1.12

Four proteins were found in significantly higher abundance in CSF from control individuals compared to patients with communicating HC

*Ctrl*, control subjects; *comm.*, communicating; *Obstr.*, obstructive; *HC*, hydrocephalus; *SD*, standard deviation, *N*, number of subjects/patients; *VIM*, vimentin; *PCDHAC2*, protocadherin alpha subfamily C2; *GSS*, glutathione synthetase; *P4HB*, prolyl 4-hydroxylase subunit beta

**Table 3:** Protein found in significantly higher abundance in controls compared to obstructive HC

Protein name	Uniprot ID	Ctrl subjects, mean (SD) [*N*]	Obstr. HC, mean (SD) [*N*]	*P* value	*P* adjusted	Fold change	Log_2_ (fold change)
VIM	P08670	18.2 (1.8) [8]	13.5 (1.5) [17]	< 0.001	0.029	0.04	− 4.64

One protein was found in significantly higher abundance in CSF from control individuals compared to patients with obstructive HC

*Ctrl*, control subjects; *comm.*, communicating; *Obstr.*, obstructive; *HC*, hydrocephalus; *SD*, standard deviation; *N*, number of subjects/patients; *VIM*, vimentin

**Table 4:** Protein found in significantly higher abundance in obstructive HC compared to communicating HC

Protein name	Uniprot ID	Comm. HC, mean (SD) [*N*]	Obstr. HC, mean (SD) [*N*]	*P* value	*P* adjusted	Fold change	Log_2_ (fold change)
SDCBP	B4DHN5	13.8 (0.7) [16]	15.3 (0.8) [14]	< 0.001	0.009	2.82	1.50

One protein was found in significantly higher abundance in CSF from obstructive HC compared to patients with communicating HC

*Ctrl*, control subjects; *comm.*, communicating; *Obstr.*, obstructive; *HC*, hydrocephalus; *N*, number of subjects/patients; *SD*, standard deviation; *SDCBP*, syndecan binding protein

### Identifying biomarkers for treatment response

**Fig. 3:**
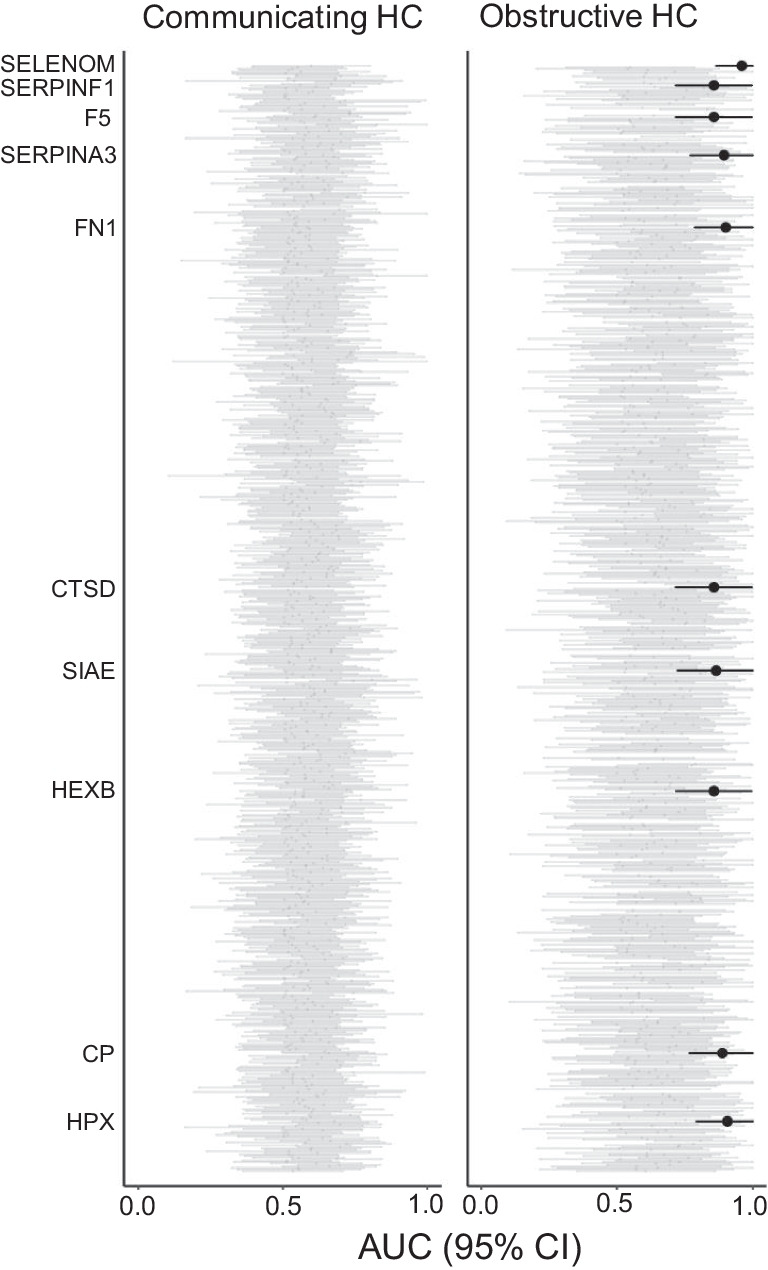
Proteomic markers as predictors of surgery-responsiveness. The figure illustrates AUC and 95% CI for the prediction of responsiveness to surgery (ETV or VP shunt) for each of the detected proteins. The proteins that reached the required AUC level of ≥ 0.7 of the lower confidence limit were accepted as a prognostic marker of responsiveness to surgery (black bars). Patients with communicating hydrocephalus: *n* = 62; patients with obstructive hydrocephalus: *n* = 28; AUC: area under the curve; CI: confidence interval; HC: hydrocephalus

**Fig. 4:**
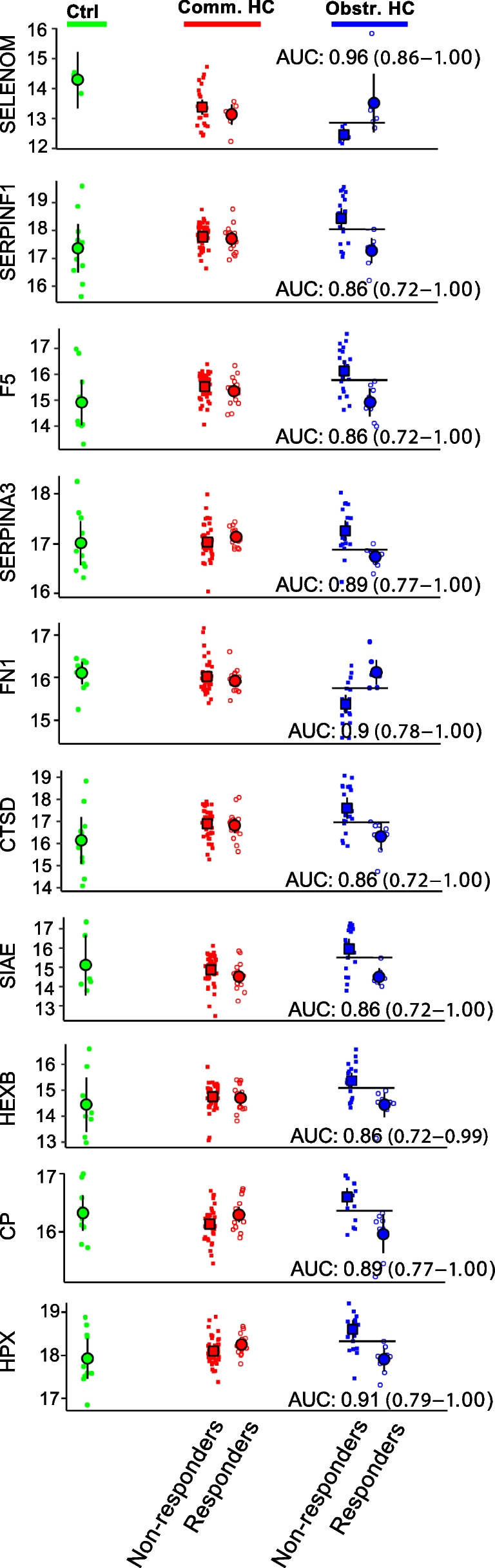
Potential predictors of treatment responsiveness in obstructive hydrocephalus. The abundance of the proteins identified in ventricular CSF samples from control subjects, responders, and non-responders in communicating and obstructive hydrocephalus. Predictors of treatment responsiveness in obstructive hydrocephalus are shown with AUC (AUC; arbitrary units with 95% confidence interval). Black lines show Youden’s threshold as a cut-off for sensitivity and specificity for the proteins found to be predictors. Patients with communicating hydrocephalus: *n* = 62; patients with obstructive hydrocephalus: *n* = 28; control subjects: *n* = 10; a.u.: arbitrary units; AUC: area under the curve; ctrl: control; comm.: communicating; obstr.: obstructive; HC: hydrocephalus

In order to address the question regarding the prediction of treatment response, we analyzed data by a receiver operating curve (ROC). Proteins with a lower confidence limit of the area under the curve (AUC) of ≥ 0.7 were considered potentially valid markers. Among patients with communicating hydrocephalus, 44 patients were categorized as responders and 16 as non-responders following surgical treatment. Twenty-eight patients with obstructive hydrocephalus were treated surgically, and 20 patients were categorized as responders and 8 patients as non-responders. For two patients, the response to treatment could not be evaluated as they had either tap test through a shunt chamber or inserted ventricular drainage. No proteins were found to be markers of treatment response for communicating hydrocephalus (Fig. 3, left panel). However, 10 proteins were found to be potential markers of treatment response in obstructive hydrocephalus (Fig. 3, right panel): ceruloplasmin (CP), cathepsin D (CTSD), coagulation factor 5 (F5), fibronectin (FN1), beta-hexosaminidase subunit beta (HEXB), hemopexin (HPX), serpin F member 1 (SERPINF1), selenoprotein M (SELENOM), serpin family A member 3 (SERPINA3), and sialate-O-acetylesterase (SIAE). Further analysis of these 10 proteins revealed significantly different levels in CSF of responders versus non-responders with eight of these proteins (CP, CTSD, F5, HEXB, HPX, SERPINF1, SERPINA3, and SIA) being significantly higher in the responder group compared to the non-responding group, whereas two (FN1 and SELENOM) were significantly lower in the responder group (Fig. 4 and Table 5).

**Table 5:** Acceptable predictors of responders/non-responders of surgical treatment

Protein name	Diagnosis	Responder, mean (SD) [*N*]	Non-responder, mean (SD) [*N*]	AUC (95%CI)	Cut-off	Sensitivity	Specificity
SELENOM	Obstr. HC	12.5 (0.24) [7]	13.5 (1.07) [7]	0.96 (0.86–1.00)	12.9	1.00	0.86
SERPINF1	Obstr. HC	18.4 (0.82) [20]	17.3 (0.55) [8]	0.86 (0.72–1.00)	18.0	0.70	1.00
F5	Obstr. HC	16.1 (0.88) [20]	14.9 (0.65) [8]	0.86 (0.72–1.00)	15.8	0.65	1.00
SERPINA3	Obstr. HC	17.3 (0.44) [20]	16.7 (0.19) [8]	0.89 (0.77–1.00)	16.9	0.90	0.88
FN1	Obstr. HC	15.4 (0.47) [20]	16.1 (0.35) [8]	0.90 (0.78–1.00)	15.8	0.80	1.00
CTSD	Obstr. HC	17.6 (1.04) [20]	16.3 (0.75) [8]	0.86 (0.72–1.00)	17.0	0.75	1.00
SIAE	Obstr. HC	16.0 (1.13) [20]	14.5 (0.48) [7]	0.86 (0.72–1.00)	15.5	0.70	1.00
HEXB	Obstr. HC	15.4 (0.63) [20]	14.4 (0.57) [8]	0.86 (0.72–0.99)	15.1	0.70	1.00
CP	Obstr. HC	16.6 (0.33) [20]	16.0 (0.39) [8]	0.89 (0.77–1.00)	16.4	0.80	1.00
HPX	Obstr. HC	18.6 (0.48) [20]	17.9 (0.33) [8]	0.91 (0.79–1.00)	18.3	0.75	1.00

Ten proteins were found to differ significantly between responders and non-responders

*AUC*, area under the curve; *CI*, confidence interval; *Obstr. HC*, obstructive hydrocephalus; *SD*, standard deviation; *CP*, ceruloplasmin; *CTSD*, cathepsin D; *F5*, coagulation factor 5; *FN1*, fibronectin; *HEXB*, beta-hexosaminidase subunit beta; *HPX*, hemopexin; *SERPINF1*, serpin F member 1; *SELENOM*, selenoprotein M; *SERPINA3*, serpin family A member 3; *SIAE*, sialate-O-acetylesterase

## Discussion

### Potential diagnostic biomarkers

By applying an unbiased MS-based proteomic approach to analyze ventricular CSF in patients with obstructive vs. non-obstructive hydrocephalus compared to a control group, we found that the proteomic profile of hydrocephalus patients differs slightly from control subjects, as a small number of proteins are present in CSF from control subjects that are less abundant in CSF from patients with hydrocephalus. There are thus a number of candidate proteins, which might be potential diagnostic biomarkers for hydrocephalus in general. Of these candidate proteins, only one protein, VIM, an intermediate filament protein predominantly known for its role in the maintenance of cell integrity [52], was found less abundant in CSF from both types of included hydrocephalus groups (communicating and obstructive) when compared to that of controls. Others have previously reported increased expression of VIM-positive cells in areas of ependymal disruption from hydrocephalic post-mortem brains [60]. Activated microglial cells are also known to express vimentin in response to brain damage or local inflammation [15]. Although the reason for the reduced CSF VIM in our hydrocephalic patient groups remains unclear, it may suggest the absence of any major structural disruptions or inflammatory responses.

The proteomic profiles of patients with communicating vs. obstructive hydrocephalus were similar with only one protein, SDCBP, found to be significantly more abundant in patients with obstructive hydrocephalus. SDCBP is a widely expressed multifunctional scaffold protein mostly known for its role in cancer progression and metastasis [47]. Whether some of the same intracellular signaling pathways involved in cancer progressions such as the focal adhesion kinase (FAK) pathway or the p38 mitogen-activated protein kinase (MAPK) pathway [47] are likewise activated in obstructive hydrocephalus, but not communicating hydrocephalus, remains unclear, and whether this single protein might be clinically useful as a biomarker to select patients with obstructive hydrocephalus and ETV as the primary treatment option needs to be investigated further. Eight proteins show a tendency to be increased in patients with obstructive hydrocephalus but did not meet our cut-off for the Bonferroni-corrected *P* value (Supplementary information 5). These might be alternatives to investigate as possible markers of obstructive hydrocephalus.

### Potential treatment predictor biomarkers

The response rates in our series were similar for shunt implantation in communicating hydrocephalus and ETV in obstructive hydrocephalus (Table 1). Approximately 30% in both groups underwent a neurosurgical procedure with the inherent risks and discomfort but without any benefit. Predictors of surgical treatment outcome of ETVs and VP shunts have been investigated extensively [14, 16, 17, 25, 34, 40, 43, 45, 48, 62, 63]; however, a robust and generally accepted scoring system for ETV success prediction in adult hydrocephalus has yet to be established [62]. Cortical biopsies were not a significant predictor of ETV success in NPH [29]. Whereas the response rate to shunt implantation in obstructive hydrocephalus and in secondary communicating hydrocephalus, e.g., secondary NPH, is very high, the prediction of treatment response in idiopathic NPH is much less clear.

Pre-surgical CSF biomarkers predicting shunt response in iNPH have been reported earlier, where the main focus has been to distinguish between iNPH and degenerative brain disease, e.g., Alzheimer’s disease [13, 22, 28, 42, 55, 69]. For this purpose, biomarkers for neurodegeneration, tau protein, beta-amyloid (Aβ), and neurofilament light (NfL) chain are widely used [3]. The biomarkers Aβ, phosphorylated and total tau, NfL, and leucine-rich alpha-2-glycoprotein show the strongest evidence for their predictive value when determining shunt responsiveness in iNPH patients [46]. In addition, an increased expression of a number of inflammatory markers, such as interleukins, seems to be associated with a lack of benefit from shunting [4, 20, 46]. In contrast to earlier studies, we here applied an unbiased approach by MS-based proteomics. We identified ten protein markers to be predictors of response to treatment for patients with obstructive hydrocephalus. The majority of the identified predictive proteins are found in higher abundance in the responder group compared to the non-responder group. The proteins which showed significantly higher abundance in the responder group compared to the non-responder group are connected to iron metabolism, heme detoxification, endo-protease, coagulation system, protease inhibitors, and downregulation B-lymphocyte antigen receptor signaling. Selenoprotein M, which was found to have a higher abundance in the non-responder group compared to responders, is highly expressed in the brain and has neuroprotective properties [50]. We did not identify any protein biomarkers as predictors of response to treatment for patients with communicating hydrocephalus. Particularly in the group of iNPH patients, biomarkers to improve prediction of shunt responsiveness would be very desirable. Interestingly, a recent study identified four CSF proteins as possible biomarkers of shunt responsiveness in iNPH patients when evaluated 1-year post-surgery [68]. The study employed lumbar CSF samples for their proteomic analysis, the protein composition of which differs from that of ventricular CSF as here employed [54]. Hence, methodological differences may explain some of these discrepancies. In the present study, we employed an unbiased MS-based proteomic approach to analyze our CSF samples. CSF studies by MS-based proteomics are challenging due to the high dynamic range of protein abundances in human biological samples, which, combined with the limited dynamic range of MS instruments, means that only the medium to most abundant proteins will be identified. However, despite limitations within the method and study design, it is important to emphasize that the proteins identified are not random, but represent pathways or structures important for hydrocephalus such as the brain parenchyma as well as the coagulation system. Both can potentially play an important biological role in chronic hydrocephalus as well as the surgical outcome afterward. This underlines the importance of further studies within this area using larger patient cohorts and more specific CSF analysis techniques such as conventional immunoassays. The complex nature of chronic hydrocephalus may require a panel of several biomarkers to reliably predict shunt responsiveness. This study aimed to identify a list of potential biomarkers that can aid future research and ultimately result in identification of biomarkers that can be utilized to improve clinical decision-making and treatment of patients with chronic hydrocephalus.

### Limitations

The present study employed a limited number of control subjects (*n* = 10) in comparison to hydrocephalus patients (*n* = 62 communicating hydrocephalus, *n* = 28 obstructive hydrocephalus). Furthermore, a limitation to our findings was that the responder group for obstructive hydrocephalus had more patients (*n* = 20) than the non-responder group (*n* = 8) and could thus explain why proteins were found in higher abundance in the responder group. However, the same was true for communicating hydrocephalus (responders *n* = 44 vs. non-responders *n* = 16), where no predictors were found in higher abundance in the responder group. The control CSF was obtained from the cisterns, whereas the CSF obtained from the hydrocephalus patients was collected from the ventricular compartment. The different sampling sites were dictated by ethical limitations but could potentially influence our results if the protein content and concentration of individual proteins differ between the ventricular and cisternal compartments. However, a recent study revealed no overall difference in cisternal and ventricular CSF protein content [21]. CSF comparison studies remain limited and have predominantly relied on control CSF collected from the lumbar compartment which differs in protein composition from that of ventricular CSF [54]. Additional studies are thus required to elucidate the extent to which the protein content differs between the different CSF compartments (ventricular, cisternal, lumbar), as any differences may constitute a potential confounding factor. It should furthermore be acknowledged that the present results were obtained based on a single time point of CSF collection but that the CSF is continuously produced and circulated through the ventricular system. We thus cannot draw any definite conclusions as to whether similar results would be obtained with CSF samples acquired at an earlier or later stage of disease progression. As disturbances in brain fluid dynamics are anticipated to be best reflected in ventricular CSF residing adjacent to the affected brain itself, we chose to analyze ventricular CSF from our cohort of patients with hydrocephalus. We cannot rule out that the surgical procedure under which the ventricular CSF samples were obtained may have led to the release of cellular debris or other proteins into the CSF, which could potentially have altered the protein content and thus our present results. Moreover, although the collected CSF samples were kept on ice before processing and stored at − 80 °C as fast as possible (within 2 h from collection), we cannot exclude that changes in the protein content may have occurred within this limited time frame. As the biomarkers identified in the present study were measured in ventricular CSF, it would be of interest to evaluate if the same biomarkers are found in lumbar CSF. This would make it possible to give an early indication of treatment outcome if the markers could be analyzed in CSF samples collected during a pre-surgical lumbar puncture which is swifter and less invasive, albeit often contraindicated in suspected obstructive hydrocephalus.

## Conclusion

Here, we show that the proteomic profile of ventricular CSF from patients with hydrocephalus differs slightly from CSF obtained from control subjects. We detected ten proteins that could serve as potential predictors of response to surgical outcomes in patients with obstructive hydrocephalus. These findings should be confirmed in a larger cohort and preferably also in lumbar CSF from patients with obstructive hydrocephalus to establish if the CSF markers can be assessed pre-surgically through the lumbar puncture and thereby aid clinicians in decision-making.

### Supplementary Information

Below is the link to the electronic supplementary material.

Supplementary file1 (PDF 170 KB)

Supplementary file2 (PDF 991 KB)

Supplementary file3 (PDF 945 KB)

Supplementary file4 (PDF 1102 KB)

Supplementary file5 (PDF 40 KB)

## Data Availability

Anonymized data available from corresponding author upon reasonable request.

## Abbreviations

Aβ: Beta-amyloid
AUC: Area under the curve
CP: Ceruloplasmin
CSF: Cerebrospinal fluid
CTSD: Cathepsin D
FAK: Focal adhesion kinase
F5: Coagulation factor 5
FN1: Fibronectin
ETV: Endoscopic third ventriculostomy
EVD: Extra-ventricular drain
GSS: Glutathione synthetase
HYDROCEPHALUS: Hydrocephalus
HEXB: Beta-hexosaminidase subunit beta
HPX: Hemopexin
MAPK: Mitogen-activated protein kinase
MS: Mass spectroscopy
NfL: Neurofilament light
NPH: Normal pressure hydrocephalus
P4HB: Prolyl 4-hydroxylase subunit beta
PCA: Principal component analysis
PCDHAC2: Protocadherin alpha subfamily C2
SD: Standard deviation
SDCBP: Syndecan binding protein
SELENOM: Selenoprotein M
SERPINA3: Serpin family A member 3
SERPINF1: Serpin F member 1
SIAE: Sialate-O-acetylesterase
VIM: Vimentin
VP: Ventriculo-peritoneal
