# Supplementary material for: Ventricular CSF proteomic profiles and predictors of surgical treatment outcome in chronic hydrocephalus
Source: Acta Neurochir (Wien). 2023 Oct 19;165(12):4059–70. doi: 10.1007/s00701-023-05832-y (PMC10739511; doi:10.1007/s00701-023-05832-y)
Supplement: Supplementary file 1 — Supplementary file1 (PDF 170 KB) [file 701_2023_5832_MOESM1_ESM.pdf]

Supplemental file 1

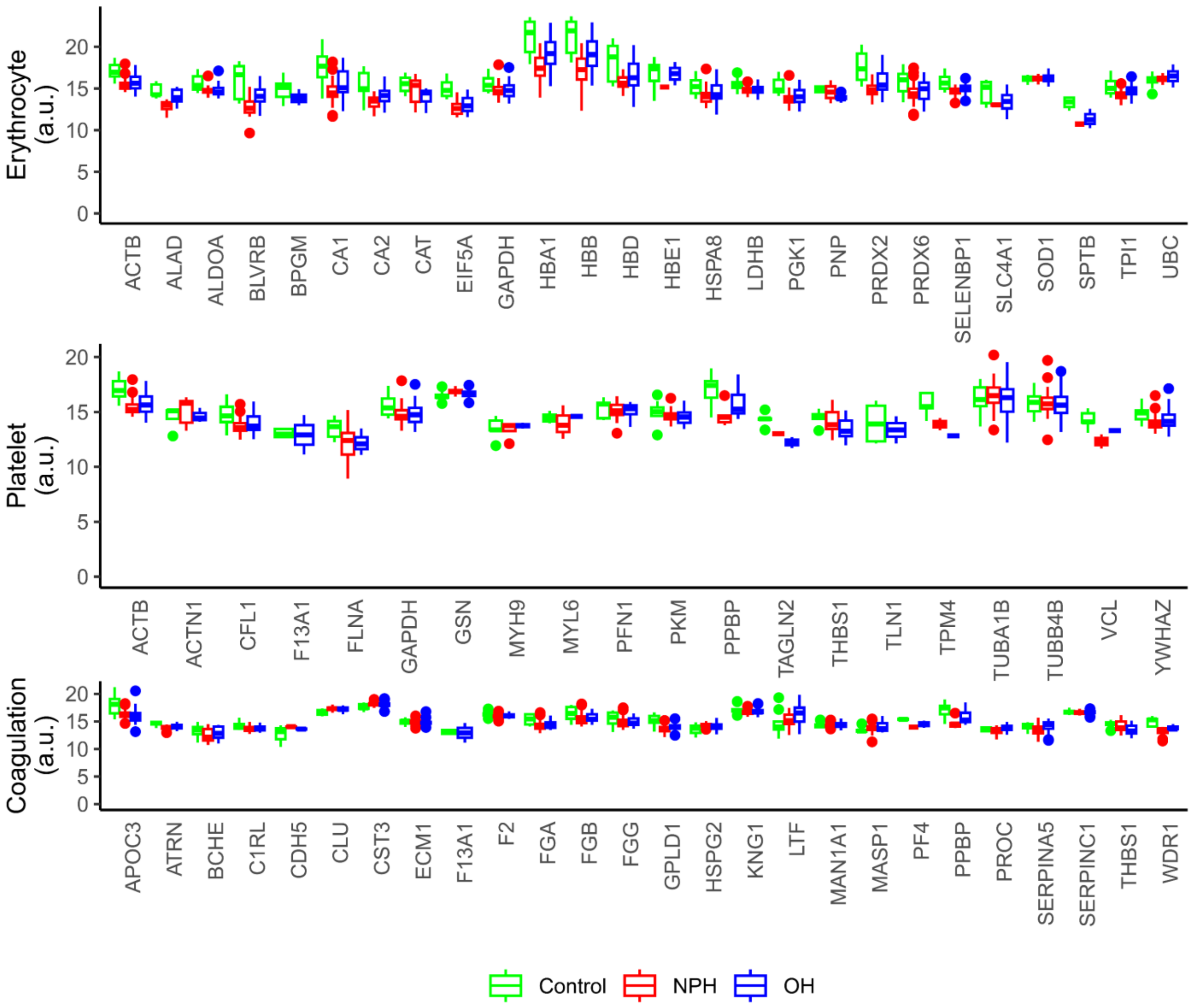

Figure S1. Blood contamination of CSF samples. No signs of blood contamination in the CSF in either the control individuals, communicating HC or obstructive HC. Visual assessment of data obtained by mass spectrometry. Data demonstrate that CSF samples in all three groups contains overall similar levels of erythrocytes, platelets and coagulation factors.
a.u.: arbitrary units; HC: hydrocephalus
