# Supplementary material for: Ventricular CSF proteomic profiles and predictors of surgical treatment outcome in chronic hydrocephalus
Source: Acta Neurochir (Wien). 2023 Oct 19;165(12):4059–70. doi: 10.1007/s00701-023-05832-y (PMC10739511; doi:10.1007/s00701-023-05832-y)
Supplement: Supplementary file 2 — Supplementary file2 (PDF 991 KB) [file 701_2023_5832_MOESM2_ESM.pdf]

**Supplemental file 2**

# Control subjects compared to communicating HC

All proteins identified by mass-spectrometry-based proteomics in at least 5 samples from control subjects and 10 samples from patients with communicating hydrocephalus (HC). Proteins that differed significantly between the two groups are highlighted in green.

| | | Control | | | Communicating HC | | | | | | |
|---|---|---|---|---|---|---|---|---|---|---|---|
| Name | Uniprot ID | Mean | (SD) | [N] | Mean | (SD) | [N] | P value | P adjusted | Fold change | $Log_2$(fold change) |
| **GSS** | P48637 | 15.6 | (0.4) | [5] | 13.6 | (0.9) | [20] | <0.001 | 0.001 | 0.24 | -2.059 |
| **PCDHAC2** | Q9Y5I4 | 14.6 | (0.3) | [6] | 13.4 | (0.9) | [50] | <0.001 | 0.002 | 0.45 | -1.152 |
| **P4HB** | P07237 | 13.4 | (0.4) | [7] | 12.3 | (0.8) | [21] | <0.001 | 0.016 | 0.46 | -1.120 |
| **IGHV3-30** | P01768 | 16.9 | (0.2) | [10] | 16.4 | (0.5) | [62] | <0.001 | 0.025 | 0.73 | -0.454 |
| **VIM** | P08670 | 18.2 | (1.8) | [8] | 12.8 | (1.0) | [49] | <0.001 | 0.034 | 0.03 | -5.059 |
| **PDIA3** | P30101 | 14.2 | (0.3) | [7] | 13.5 | (0.5) | [53] | <0.001 | 0.037 | 0.60 | -0.737 |
| **CLSTN3** | Q9BQT9 | 11.3 | (0.8) | [10] | 12.8 | (1.7) | [62] | <0.001 | 0.063 | 2.95 | 1.561 |
| **HBB** | P68871 | 21.2 | (2.1) | [10] | 17.3 | (1.6) | [62] | <0.001 | 0.113 | 0.07 | -3.837 |
| **IGKV3D-20** | A0A0C4DH25 | 18.4 | (0.5) | [10] | 17.6 | (0.8) | [62] | <0.001 | 0.178 | 0.57 | -0.811 |
| **HBA1** | P69905 | 21.1 | (2.1) | [10] | 17.6 | (1.5) | [62] | <0.001 | 0.236 | 0.08 | -3.644 |
| **IGFALS** | P35858 | 15.2 | (0.7) | [10] | 14.1 | (0.5) | [62] | <0.001 | 0.262 | 0.47 | -1.089 |
| **EIF5A** | I3L397 | 15.0 | (1.1) | [8] | 12.6 | (0.8) | [37] | <0.001 | 0.265 | 0.20 | -2.322 |
| **ACTB** | P60709 | 17.1 | (1.1) | [10] | 15.4 | (0.6) | [62] | 0.001 | 0.335 | 0.30 | -1.737 |
| **ATRN** | O75882 | 14.6 | (0.4) | [9] | 13.9 | (0.3) | [56] | 0.001 | 0.335 | 0.63 | -0.667 |
| **GPI** | A0A0A0MTS2 | 13.6 | (0.6) | [8] | 12.5 | (0.6) | [37] | 0.001 | 0.361 | 0.45 | -1.152 |
| **SH3BGRL3** | Q5T123 | 14.0 | (0.6) | [7] | 12.7 | (0.6) | [22] | 0.001 | 0.427 | 0.40 | -1.322 |

| Name | Uniprot ID | Control | | | Communicating HC | | | P value | P adjusted | Fold change | $Log_2$(fold change) |
|---|---|---|---|---|---|---|---|---|---|---|---|
| | | Mean | (SD) | [N] | Mean | (SD) | [N] | | | | |
| **ACTBL2** | Q562R1 | 18.9 | (1.1) | [10] | 17.2 | (0.7) | [62] | 0.001 | 0.441 | 0.30 | -1.737 |
| **HSPA1B** | A0A0G2JIW1 | 15.5 | (1.0) | [9] | 13.9 | (0.9) | [41] | 0.001 | 0.517 | 0.31 | -1.690 |
| **PGK1** | P00558 | 15.2 | (1.0) | [10] | 13.8 | (0.7) | [58] | 0.001 | 0.545 | 0.37 | -1.434 |
| **CNTFR** | P26992 | 13.7 | (0.4) | [8] | 13.0 | (0.8) | [59] | 0.001 | 0.61 | 0.62 | -0.690 |
| **SH3BGRL** | O75368 | 13.8 | (0.7) | [7] | 12.5 | (0.5) | [27] | 0.001 | 0.696 | 0.40 | -1.322 |
| **SLC39A10** | Q9ULF5 | 13.9 | (0.6) | [6] | 12.5 | (0.7) | [48] | 0.001 | 0.705 | 0.40 | -1.322 |
| **ITIH4** | Q14624 | 16.6 | (0.8) | [10] | 15.4 | (0.4) | [62] | 0.001 | 0.722 | 0.46 | -1.120 |
| **HBG2** | P69892 | 16.3 | (1.5) | [9] | 13.9 | (1.9) | [31] | 0.001 | 0.92 | 0.20 | -2.322 |
| **TFRC** | G3V0E5 | 11.5 | (0.3) | [7] | 11.0 | (0.4) | [19] | 0.001 | 0.926 | 0.70 | -0.515 |
| **CA1** | P00915 | 17.5 | (2.1) | [10] | 14.6 | (1.3) | [45] | 0.001 | 0.948 | 0.13 | -2.943 |
| **PRDX2** | P32119 | 17.6 | (1.9) | [10] | 14.9 | (0.8) | [62] | 0.002 | 0.999 | 0.15 | -2.737 |
| **A1BG** | P04217-2 | 17.1 | (0.5) | [10] | 16.9 | (0.3) | [62] | 0.27 | 1 | 0.87 | -0.201 |
| **A2M** | P01023 | 17.2 | (0.7) | [10] | 16.7 | (0.3) | [62] | 0.031 | 1 | 0.68 | -0.556 |
| **ABHD14B** | Q96IU4 | 14.1 | (0.7) | [8] | 12.3 | (0.5) | [36] | 0.005 | 1 | 0.30 | -1.737 |
| **ACSBG2** | Q5FVE4 | 20.3 | (0.7) | [10] | 21.8 | (0.8) | [62] | 0.006 | 1 | 2.69 | 1.428 |
| **ACTA2** | P62736 | 16.5 | (0.7) | [10] | 15.4 | (0.8) | [61] | 0.004 | 1 | 0.45 | -1.152 |
| **ACYP2** | P14621 | 12.5 | (0.7) | [7] | 12.0 | (0.4) | [56] | 0.111 | 1 | 0.71 | -0.494 |
| **ADAM22** | F8WAD8 | 12.0 | (0.7) | [9] | 12.8 | (1.7) | [62] | 0.125 | 1 | 1.66 | 0.731 |
| **ADAMTS1** | Q9UHI8 | 14.7 | (0.7) | [5] | 14.5 | (0.3) | [48] | 0.157 | 1 | 0.90 | -0.152 |
| **ADGRB2** | A2A3C1 | 15.4 | (0.7) | [9] | 15.2 | (0.6) | [62] | 0.595 | 1 | 0.88 | -0.184 |
| **ADGRB3** | O60242 | 12.2 | (0.7) | [5] | 11.4 | (0.7) | [55] | 0.187 | 1 | 0.60 | -0.737 |

| Name | Uniprot ID | Control | | | Communicating HC | | | P value | P adjusted | Fold change | $Log_2$(fold change) |
|---|---|---|---|---|---|---|---|---|---|---|---|
| | | Mean | (SD) | [N] | Mean | (SD) | [N] | | | | |
| **ADGRL1** | O94910 | 14.1 | (0.7) | [7] | 13.8 | (0.6) | [60] | 0.072 | 1 | 0.82 | -0.286 |
| **ADGRL3** | E7EN28 | 13.8 | (0.7) | [6] | 13.8 | (0.9) | [57] | 0.905 | 1 | 1.02 | 0.029 |
| **ADIPOQ** | Q15848 | 13.0 | (0.7) | [8] | 11.8 | (0.4) | [50] | 0.017 | 1 | 0.43 | -1.218 |
| **AEBP1** | Q8IUX7 | 13.6 | (0.7) | [7] | 13.8 | (0.5) | [61] | 0.229 | 1 | 1.19 | 0.251 |
| **AFM** | P43652 | 15.2 | (0.7) | [10] | 15.1 | (0.5) | [62] | 0.49 | 1 | 0.95 | -0.074 |
| **AGA** | P20933 | 13.9 | (0.7) | [6] | 13.9 | (2.0) | [61] | 0.899 | 1 | 0.96 | -0.059 |
| **AGRN** | O00468-6 | 13.9 | (0.7) | [9] | 14.2 | (0.6) | [62] | 0.46 | 1 | 1.18 | 0.239 |
| **AGT** | P01019 | 16.9 | (0.7) | [10] | 17.0 | (0.4) | [62] | 0.473 | 1 | 1.12 | 0.163 |
| **AHSG** | P02765 | 17.0 | (0.7) | [10] | 16.7 | (0.4) | [62] | 0.163 | 1 | 0.81 | -0.304 |
| **AK1** | P00568 | 15.2 | (0.7) | [6] | 14.3 | (0.9) | [25] | 0.192 | 1 | 0.52 | -0.943 |
| **ALAD** | P13716 | 14.7 | (0.7) | [5] | 12.9 | (0.7) | [12] | 0.007 | 1 | 0.28 | -1.837 |
| **ALB** | P02768 | 19.1 | (0.7) | [10] | 19.4 | (0.3) | [62] | 0.015 | 1 | 1.21 | 0.275 |
| **ALCAM** | Q13740 | 14.7 | (0.7) | [10] | 15.3 | (0.3) | [62] | 0.1 | 1 | 1.54 | 0.623 |
| **ALDOA** | P04075 | 15.7 | (0.7) | [10] | 14.7 | (0.4) | [62] | 0.02 | 1 | 0.52 | -0.943 |
| **ALDOC** | P09972 | 14.4 | (0.7) | [10] | 14.7 | (0.4) | [62] | 0.159 | 1 | 1.28 | 0.356 |
| **AMBP** | P02760 | 16.1 | (0.7) | [10] | 15.7 | (0.5) | [62] | 0.061 | 1 | 0.72 | -0.474 |
| **ANXA5** | P08758 | 15.7 | (0.7) | [9] | 14.1 | (0.7) | [30] | 0.004 | 1 | 0.31 | -1.690 |
| **APCS** | P02743 | 16.0 | (0.7) | [10] | 14.3 | (0.6) | [62] | 0.026 | 1 | 0.33 | -1.599 |
| **APLP1** | B7Z4G8 | 16.4 | (0.7) | [10] | 17.0 | (0.3) | [62] | 0.034 | 1 | 1.5 | 0.585 |
| **APLP2** | Q06481 | 14.9 | (0.7) | [10] | 14.9 | (0.6) | [62] | 0.966 | 1 | 1.01 | 0.014 |
| **APOA1** | P02647 | 18.8 | (0.7) | [10] | 18.2 | (0.4) | [62] | 0.137 | 1 | 0.67 | -0.578 |

| Name | Uniprot ID | Control | | | Communicating HC | | | P value | P adjusted | Fold change | Log₂(fold change) |
|---|---|---|---|---|---|---|---|---|---|---|---|
| | | Mean | (SD) | [N] | Mean | (SD) | [N] | | | | |
| **APOA2** | P02652 | 18.0 | (0.7) | [10] | 17.2 | (0.5) | [62] | 0.004 | 1 | 0.57 | -0.811 |
| **APOA4** | P06727 | 16.7 | (0.7) | [10] | 16.6 | (0.5) | [62] | 0.694 | 1 | 0.93 | -0.105 |
| **APOB** | P04114 | 16.4 | (0.7) | [10] | 13.7 | (1) [ | 61] | 0.002 | 1 | 0.16 | -2.644 |
| **APOC1** | K7ERI9 | 16.5 | (0.7) | [10] | 15.4 | (0.6) | [62] | 0.021 | 1 | 0.46 | -1.120 |
| **APOC3** | B0YIW2 | 18.1 | (0.7) | [10] | 16.2 | (0.7) | [62] | 0.02 | 1 | 0.28 | -1.837 |
| **APOC4-APOC2** | A0A024R0T9 | 15.9 | (0.7) | [10] | 14.4 | (0.9) | [53] | 0.014 | 1 | 0.34 | -1.556 |
| **APOD** | C9JF17 | 16.5 | (0.7) | [10] | 17.0 | (0.3) | [62] | 0.055 | 1 | 1.43 | 0.516 |
| **APOE** | P02649 | 17.6 | (0.7) | [10] | 17.7 | (0.5) | [62] | 0.611 | 1 | 1.08 | 0.111 |
| **APOH** | P02749 | 15.8 | (0.7) | [10] | 15.6 | (0.4) | [62] | 0.224 | 1 | 0.84 | -0.252 |
| **APOL1** | O14791 | 14.9 | (0.7) | [9] | 12.9 | (0.8) | [52] | 0.015 | 1 | 0.26 | -1.943 |
| **APOM** | O95445 | 16.5 | (0.7) | [9] | 15.3 | (0.5) | [62] | 0.024 | 1 | 0.44 | -1.184 |
| **APP** | P05067 | 15.2 | (0.7) | [10] | 15.2 | (0.4) | [62] | 0.808 | 1 | 1.04 | 0.057 |
| **ARPC4-TTLL3** | A0A0A6YYG9 | 16.3 | (0.7) | [10] | 16.5 | (0.4) | [62] | 0.177 | 1 | 1.12 | 0.163 |
| **ART3** | E7ESB3 | 14.1 | (0.7) | [10] | 15.1 | (0.4) | [62] | 0.028 | 1 | 1.95 | 0.963 |
| **ASAH1** | A0A1B0GTM3 | 14.8 | (0.7) | [5] | 14.8 | (0.6) | [58] | 0.991 | 1 | 1.00 | 0.000 |
| **ATP1A1** | P05023 | 14.2 | (0.7) | [5] | 13.8 | (0.9) | [24] | 0.305 | 1 | 0.77 | -0.377 |
| **ATP6AP1** | Q15904 | 15.1 | (0.7) | [10] | 15.1 | (0.4) | [62] | 0.978 | 1 | 1.01 | 0.014 |
| **AXL** | P30530 | 14.7 | (0.7) | [7] | 15.1 | (1.0) | [45] | 0.377 | 1 | 1.37 | 0.454 |
| **AZGP1** | P25311 | 16.4 | (0.7) | [10] | 16.8 | (0.3) | [62] | 0.018 | 1 | 1.28 | 0.356 |
| **B2M** | P61769 | 16.4 | (0.7) | [10] | 17.1 | (0.4) | [62] | 0.034 | 1 | 1.62 | 0.696 |
| **B3GALNT1** | O75752 | 13.2 | (0.7) | [7] | 13.4 | (0.6) | [58] | 0.213 | 1 | 1.16 | 0.214 |

| Name | Uniprot ID | Control Mean | Control (SD) | Control [N] | Communicating HC Mean | Communicating HC (SD) | Communicating HC [N] | P value | P adjusted | Fold change | $Log_2$(fold change) |
|---|---|---|---|---|---|---|---|---|---|---|---|
| **B4GAT1** | O43505 | 16.5 | (0.7) | [10] | 17.0 | (0.7) | [62] | 0.208 | 1 | 1.36 | 0.444 |
| **BASP1** | P80723 | 11.4 | (0.7) | [10] | 11.9 | (0.7) | [61] | 0.109 | 1 | 1.42 | 0.506 |
| **BCAN** | Q96GW7 | 15.3 | (0.7) | [10] | 16.1 | (0.6) | [62] | 0.020 | 1 | 1.72 | 0.782 |
| **BGN** | P21810 | 14.9 | (0.7) | [8] | 13.5 | (1.0) | [62] | 0.004 | 1 | 0.39 | -1.358 |
| **BLVRB** | P30043 | 15.8 | (0.7) | [9] | 12.7 | (1.1) | [23] | 0.002 | 1 | 0.12 | -3.059 |
| **BTD** | P43251 | 15.8 | (0.7) | [10] | 16.3 | (0.3) | [62] | 0.047 | 1 | 1.44 | 0.526 |
| **C16orf89** | A0A0A0MT71 | 14.4 | (0.7) | [9] | 14.6 | (0.4) | [62] | 0.482 | 1 | 1.17 | 0.227 |
| **C1QA** | P02745 | 14.8 | (0.7) | [9] | 14.5 | (0.5) | [62] | 0.133 | 1 | 0.82 | -0.286 |
| **C1QB** | D6R934 | 15.4 | (0.7) | [10] | 15.4 | (0.4) | [62] | 0.841 | 1 | 0.97 | -0.044 |
| **C1QC** | P02747 | 17.1 | (0.7) | [10] | 16.8 | (0.4) | [62] | 0.220 | 1 | 0.83 | -0.269 |
| **C1QTNF5** | Q9BXJ0 | 13.6 | (0.7) | [5] | 13.2 | (0.7) | [59] | 0.602 | 1 | 0.78 | -0.358 |
| **C1R** | B4DPQ0 | 15.6 | (0.7) | [10] | 15.6 | (0.3) | [62] | 0.951 | 1 | 0.99 | -0.014 |
| **C1RL** | Q9NZP8 | 14.3 | (0.7) | [9] | 13.7 | (0.5) | [61] | 0.049 | 1 | 0.68 | -0.556 |
| **C1S** | P09871 | 16.3 | (0.7) | [10] | 16.3 | (0.3) | [62] | 0.911 | 1 | 0.99 | -0.014 |
| **C2** | P06681 | 15.0 | (0.7) | [10] | 14.6 | (0.3) | [62] | 0.049 | 1 | 0.79 | -0.340 |
| **C2orf40** | B8ZZE5 | 15.2 | (0.7) | [9] | 16.1 | (0.9) | [62] | 0.203 | 1 | 1.90 | 0.926 |
| **C3** | P01024 | 17.3 | (0.7) | [10] | 17.0 | (0.2) | [62] | 0.045 | 1 | 0.81 | -0.304 |
| **C4A** | A0A0G2JPR0 | 15.6 | (0.7) | [10] | 15.5 | (0.7) | [62] | 0.632 | 1 | 0.91 | -0.136 |
| **C4B** | P0C0L5 | 16.8 | (0.7) | [10] | 16.8 | (0.3) | [62] | 0.815 | 1 | 0.98 | -0.029 |
| **C4BPA** | P04003 | 14.2 | (0.7) | [9] | 12.5 | (1.3) | [37] | 0.024 | 1 | 0.30 | -1.737 |
| **C5** | P01031 | 14.7 | (0.7) | [10] | 13.9 | (0.4) | [62] | 0.003 | 1 | 0.55 | -0.862 |

| Name | Uniprot ID | Control Mean | Control (SD) | Control [N] | Communicating HC Mean | Communicating HC (SD) | Communicating HC [N] | P value | P adjusted | Fold change | $Log_2$(fold change) |
|---|---|---|---|---|---|---|---|---|---|---|---|
| **C6** | P13671 | 15.5 | (0.7) | [10] | 14.8 | (0.4) | [62] | 0.010 | 1 | 0.63 | -0.667 |
| **C7** | P10643 | 15.8 | (0.7) | [10] | 16.1 | (0.4) | [62] | 0.119 | 1 | 1.26 | 0.333 |
| **C8A** | P07357 | 15.3 | (0.7) | [10] | 15.3 | (0.4) | [62] | 0.735 | 1 | 0.96 | -0.059 |
| **C8B** | F5GY80 | 14.1 | (0.7) | [10] | 14.0 | (0.3) | [62] | 0.288 | 1 | 0.91 | -0.136 |
| **C8G** | P07360 | 14.5 | (0.7) | [6] | 14.2 | (0.8) | [56] | 0.34 | 1 | 0.83 | -0.269 |
| **C9** | P02748 | 15.5 | (0.7) | [10] | 15.2 | (0.4) | [62] | 0.104 | 1 | 0.85 | -0.234 |
| **CA2** | P00918 | 15.4 | (0.7) | [9] | 13.4 | (0.7) | [39] | 0.007 | 1 | 0.25 | -2.000 |
| **CACHD1** | A0A0A0MQY7 | 13.3 | (0.7) | [8] | 13.3 | (0.5) | [61] | 0.813 | 1 | 1.04 | 0.057 |
| **CACNA2D1** | P54289 | 14.6 | (0.7) | [10] | 14.8 | (0.3) | [62] | 0.277 | 1 | 1.19 | 0.251 |
| **CADM1** | A0A087X0T8 | 15.7 | (0.7) | [8] | 16.1 | (0.5) | [61] | 0.431 | 1 | 1.24 | 0.310 |
| **CADM2** | Q8N3J6 | 14.6 | (0.7) | [6] | 14.8 | (0.5) | [62] | 0.387 | 1 | 1.12 | 0.163 |
| **CADM3** | Q8N126 | 15.3 | (0.7) | [10] | 15.8 | (0.4) | [62] | 0.071 | 1 | 1.33 | 0.411 |
| **CADM4** | Q8NFZ8 | 15.9 | (0.7) | [9] | 16.4 | (0.4) | [62] | 0.117 | 1 | 1.40 | 0.485 |
| **CALR** | P27797 | 13.5 | (0.7) | [10] | 14.0 | (0.3) | [62] | 0.136 | 1 | 1.44 | 0.526 |
| **CAMK2A** | Q9UQM7 | 14.7 | (0.7) | [5] | 14.2 | (0.7) | [45] | 0.207 | 1 | 0.74 | -0.434 |
| **CAMK2G** | A0A0A0MS52 | 13.2 | (0.7) | [5] | 13.2 | (0.7) | [18] | 0.928 | 1 | 1.01 | 0.014 |
| **CANT1** | Q8WVQ1 | 13.8 | (0.7) | [6] | 13.3 | (0.5) | [48] | 0.016 | 1 | 0.69 | -0.535 |
| **CARTPT** | Q16568 | 13.7 | (0.7) | [9] | 13.7 | (0.6) | [61] | 0.908 | 1 | 0.98 | -0.029 |
| **CASP14** | P31944 | 14.0 | (0.7) | [7] | 14.3 | (0.9) | [34] | 0.262 | 1 | 1.21 | 0.275 |
| **CAT** | P04040 | 15.5 | (0.7) | [7] | 14.8 | (1.5) | [29] | 0.174 | 1 | 0.60 | -0.737 |
| **CBLN1** | P23435 | 13.4 | (0.7) | [5] | 12.6 | (0.8) | [47] | 0.278 | 1 | 0.59 | -0.761 |

| Name | Uniprot ID | Control | | | Communicating HC | | | P value | P adjusted | Fold change | Log$_2$(fold change) |
|---|---|---|---|---|---|---|---|---|---|---|---|
| | | Mean | (SD) | [N] | Mean | (SD) | [N] | | | | |
| **CBR1** | P16152 | 14.7 | (0.7) | [8] | 14.2 | (0.7) | [60] | 0.218 | 1 | 0.73 | -0.454 |
| **CCDC93** | F8W9X7 | 14.1 | (0.7) | [5] | 14.4 | (0.5) | [55] | 0.020 | 1 | 1.28 | 0.356 |
| **CCP110** | O43303 | 21.1 | (0.7) | [7] | 21.6 | (0.6) | [41] | 0.112 | 1 | 1.43 | 0.516 |
| **CD14** | P08571 | 16.0 | (0.7) | [10] | 16.4 | (0.4) | [62] | 0.236 | 1 | 1.32 | 0.401 |
| **CD44** | H0YD13 | 16.5 | (0.7) | [10] | 17.1 | (0.5) | [62] | 0.032 | 1 | 1.59 | 0.669 |
| **CD59** | E9PNW4 | 15.1 | (0.7) | [8] | 15.2 | (0.9) | [62] | 0.884 | 1 | 1.04 | 0.057 |
| **CD9** | A6NNI4 | 15.5 | (0.7) | [5] | 15.2 | (0.5) | [26] | 0.708 | 1 | 0.83 | -0.269 |
| **CD99** | P14209 | 15.4 | (0.7) | [8] | 16.4 | (0.8) | [62] | 0.022 | 1 | 1.90 | 0.926 |
| **CD99L2** | Q8TCZ2 | 16.2 | (0.7) | [10] | 16.9 | (0.5) | [62] | 0.003 | 1 | 1.63 | 0.705 |
| **CDH13** | P55290 | 15.3 | (0.7) | [10] | 15.5 | (0.4) | [62] | 0.416 | 1 | 1.15 | 0.202 |
| **CDH2** | P19022 | 15.7 | (0.7) | [10] | 16.3 | (0.3) | [62] | 0.076 | 1 | 1.44 | 0.526 |
| **CDH4** | P55283 | 13.7 | (0.7) | [6] | 13.3 | (0.7) | [62] | 0.282 | 1 | 0.77 | -0.377 |
| **CDH6** | D6RF86 | 13.2 | (0.7) | [6] | 13.2 | (0.5) | [61] | 0.977 | 1 | 1.01 | 0.014 |
| **CETP** | P11597 | 15.6 | (0.7) | [6] | 15.3 | (0.4) | [46] | 0.274 | 1 | 0.81 | -0.304 |
| **CFD** | K7ERG9 | 15.7 | (0.7) | [10] | 16.4 | (0.4) | [62] | 0.005 | 1 | 1.65 | 0.722 |
| **CFH** | P08603 | 16.0 | (0.7) | [10] | 15.5 | (0.3) | [62] | 0.013 | 1 | 0.72 | -0.474 |
| **CFHR1** | B1AKG0 | 15.4 | (0.7) | [10] | 14.8 | (0.7) | [62] | 0.067 | 1 | 0.67 | -0.578 |
| **CFI** | E7ETH0 | 14.7 | (0.7) | [10] | 14.6 | (0.3) | [62] | 0.092 | 1 | 0.90 | -0.152 |
| **CFL1** | E9PK25 | 14.8 | (0.7) | [10] | 13.6 | (0.6) | [61] | 0.013 | 1 | 0.45 | -1.152 |
| **CGREF1** | Q99674 | 13.8 | (0.7) | [10] | 13.9 | (0.5) | [62] | 0.820 | 1 | 1.06 | 0.084 |
| **CHGA** | P10645 | 16.1 | (0.7) | [10] | 16.6 | (0.4) | [62] | 0.036 | 1 | 1.45 | 0.536 |

| Name | Uniprot ID | Control Mean | Control (SD) | Control [N] | Communicating HC Mean | Communicating HC (SD) | Communicating HC [N] | P value | P adjusted | Fold change | $Log_2$(fold change) |
|---|---|---|---|---|---|---|---|---|---|---|---|
| **CHGB** | P05060 | 16.0 | (0.7) | [10] | 16.3 | (0.5) | [62] | 0.199 | 1 | 1.22 | 0.287 |
| **CHI3L1** | P36222 | 15.4 | (0.7) | [10] | 16.4 | (0.5) | [62] | 0.048 | 1 | 1.97 | 0.978 |
| **CHI3L2** | Q15782 | 12.6 | (0.7) | [5] | 13.2 | (0.7) | [60] | 0.522 | 1 | 1.51 | 0.595 |
| **CHL1** | O00533 | 15.4 | (0.7) | [10] | 15.7 | (0.4) | [62] | 0.298 | 1 | 1.22 | 0.287 |
| **CHST10** | O43529 | 17.7 | (0.7) | [5] | 18.0 | (2.2) | [47] | 0.770 | 1 | 1.2 | 0.263 |
| **CKB** | P12277 | 15.4 | (0.7) | [9] | 15.4 | (1.1) | [60] | 0.967 | 1 | 0.99 | -0.014 |
| **CKM** | P06732 | 17.7 | (0.7) | [6] | 14.7 | (1.1) | [52] | 0.039 | 1 | 0.13 | -2.943 |
| **CLCNKB** | A0A087X136 | 16.2 | (0.7) | [10] | 16.5 | (0.6) | [62] | 0.297 | 1 | 1.25 | 0.322 |
| **CLEC11A** | Q9Y240 | 13.0 | (0.7) | [7] | 13.1 | (0.7) | [51] | 0.834 | 1 | 1.04 | 0.057 |
| **CLEC3B** | E9PHK0 | 16.4 | (0.7) | [10] | 16.9 | (0.3) | [62] | 0.005 | 1 | 1.41 | 0.496 |
| **CLN5** | A0A024R644 | 14.4 | (0.7) | [5] | 13.9 | (0.6) | [53] | 0.439 | 1 | 0.71 | -0.494 |
| **CLSTN1** | O94985 | 16.4 | (0.7) | [10] | 16.7 | (0.4) | [62] | 0.318 | 1 | 1.18 | 0.239 |
| **CLU** | P10909 | 16.6 | (0.7) | [10] | 17.3 | (0.3) | [62] | 0.003 | 1 | 1.57 | 0.651 |
| **CNDP1** | Q96KN2 | 16.7 | (0.7) | [10] | 17.2 | (0.4) | [62] | 0.025 | 1 | 1.43 | 0.516 |
| **CNTN1** | Q12860 | 15.5 | (0.7) | [10] | 16.1 | (0.4) | [62] | 0.057 | 1 | 1.46 | 0.546 |
| **CNTN2** | A0A1W2PQ11 | 15.3 | (0.7) | [10] | 16.2 | (0.5) | [62] | 0.055 | 1 | 1.78 | 0.832 |
| **CNTNAP4** | A0A087WTA1 | 14.3 | (0.7) | [7] | 14.2 | (0.7) | [61] | 0.708 | 1 | 0.92 | -0.120 |
| **COL18A1** | P39060 | 14.8 | (0.7) | [9] | 15.3 | (0.3) | [62] | 0.09 | 1 | 1.36 | 0.444 |
| **COL1A1** | P02452 | 15.6 | (0.7) | [9] | 14.3 | (0.5) | [62] | 0.037 | 1 | 0.41 | -1.286 |
| **COL1A2** | A0A087WTA8 | 15.3 | (0.7) | [10] | 14.2 | (0.3) | [62] | 0.063 | 1 | 0.44 | -1.184 |
| **COL2A1** | P02458 | 18.3 | (0.7) | [7] | 15.0 | (0.7) | [18] | 0.006 | 1 | 0.10 | -3.322 |

| Name | Uniprot ID | Control Mean | Control (SD) | Control [N] | Communicating HC Mean | Communicating HC (SD) | Communicating HC [N] | P value | P adjusted | Fold change | $Log_2$(fold change) |
|---|---|---|---|---|---|---|---|---|---|---|---|
| **COL3A1** | P02461 | 13.9 | (0.7) | [5] | 13.2 | (0.8) | [45] | 0.377 | 1 | 0.62 | -0.690 |
| **COL6A1** | A0A087X0S5 | 14.8 | (0.7) | [10] | 15.0 | (0.4) | [62] | 0.333 | 1 | 1.19 | 0.251 |
| **COL6A3** | P12111 | 13.9 | (0.7) | [8] | 13.7 | (0.4) | [62] | 0.295 | 1 | 0.86 | -0.218 |
| **COLEC12** | Q5KU26 | 14.2 | (0.7) | [9] | 14.5 | (0.5) | [60] | 0.295 | 1 | 1.24 | 0.310 |
| **CP** | P00450 | 16.3 | (0.7) | [10] | 16.2 | (0.3) | [62] | 0.316 | 1 | 0.90 | -0.152 |
| **CPB2** | A0A087WSY5 | 15.4 | (0.7) | [10] | 15.1 | (0.4) | [62] | 0.051 | 1 | 0.82 | -0.286 |
| **CPE** | P16870 | 16.5 | (0.7) | [10] | 17.0 | (0.5) | [62] | 0.182 | 1 | 1.43 | 0.516 |
| **CPN2** | P22792 | 15.3 | (0.7) | [10] | 14.0 | (0.6) | 5[7] | 0.007 | 1 | 0.38 | -1.396 |
| **CPQ** | Q9Y646 | 15.3 | (0.7) | [10] | 15.7 | (0.6) | [62] | 0.200 | 1 | 1.38 | 0.465 |
| **CPVL** | Q9H3G5 | 14.7 | (0.7) | [8] | 14.8 | (0.6) | [62] | 0.910 | 1 | 1.03 | 0.043 |
| **CRP** | P02741 | 14.0 | (0.7) | [5] | 14.0 | (1.0) | [37] | 0.983 | 1 | 0.99 | -0.014 |
| **CRTAC1** | A0A0C4DFP6 | 15.3 | (0.7) | [10] | 15.6 | (0.3) | [62] | 0.153 | 1 | 1.24 | 0.310 |
| **CSF1** | P09603 | 14.5 | (0.7) | [10] | 14.6 | (0.4) | [62] | 0.609 | 1 | 1.09 | 0.124 |
| **CSF1R** | E9PEK4 | 15.5 | (0.7) | [8] | 15.5 | (0.4) | [62] | 0.99 | 1 | 1.00 | 0.000 |
| **CST3** | P01034 | 17.6 | (0.7) | [10] | 18.1 | (0.3) | [62] | 0.017 | 1 | 1.49 | 0.575 |
| **CSTB** | P04080 | 15.3 | (0.7) | [10] | 13.9 | (0.6) | [49] | 0.003 | 1 | 0.40 | -1.322 |
| **CTBS** | Q01459 | 14.5 | (0.7) | [8] | 14.8 | (0.4) | [62] | 0.254 | 1 | 1.25 | 0.322 |
| **CTSA** | P10619 | 13.6 | (0.7) | [6] | 13.4 | (0.5) | [53] | 0.722 | 1 | 0.90 | -0.152 |
| **CTSB** | P07858 | 13.6 | (0.7) | [9] | 13.6 | (0.6) | [59] | 0.875 | 1 | 0.96 | -0.059 |
| **CTSC** | P53634 | 14.1 | (0.7) | [5] | 13.2 | (0.5) | [37] | 0.065 | 1 | 0.53 | -0.916 |
| **CTSD** | A0A1B0GV23 | 16.1 | (0.7) | [10] | 16.9 | (0.6) | [62] | 0.152 | 1 | 1.68 | 0.748 |

| Name | Uniprot ID | Control | | | Communicating HC | | | P value | P adjusted | Fold change | $Log_2$(fold change) |
|---|---|---|---|---|---|---|---|---|---|---|---|
| | | Mean | (SD) | [N] | Mean | (SD) | [N] | | | | |
| **CTSF** | Q9UBX1 | 14.5 | (0.7) | [6] | 14.8 | (0.3) | [60] | 0.462 | 1 | 1.22 | 0.287 |
| **CTSH** | A0A087X0D5 | 14.6 | (0.7) | [9] | 15.4 | (0.5) | [60] | 0.086 | 1 | 1.72 | 0.782 |
| **CTSL** | P07711 | 15.3 | (0.7) | [8] | 15.2 | (0.5) | [62] | 0.964 | 1 | 0.99 | -0.014 |
| **CTSS** | P25774 | 14.5 | (0.7) | [8] | 14.1 | (0.5) | [57] | 0.034 | 1 | 0.76 | -0.396 |
| **CTSZ** | Q9UBR2 | 15.5 | (0.7) | [8] | 15.5 | (0.6) | [62] | 0.873 | 1 | 0.96 | -0.059 |
| **CUTA** | O60888 | 15.2 | (0.7) | [9] | 15.2 | (0.8) | [61] | 0.84 | 1 | 1.05 | 0.070 |
| **CYCS** | C9JFR7 | 13.5 | (0.7) | [9] | 13.3 | (0.5) | [58] | 0.564 | 1 | 0.88 | -0.184 |
| **DAG1** | Q14118 | 15.3 | (0.7) | [10] | 15.8 | (0.3) | [62] | 0.019 | 1 | 1.4 | 0.485 |
| **DBI** | A0A0A0MTI5 | 14.7 | (0.7) | [10] | 15.2 | (0.5) | [62] | 0.029 | 1 | 1.42 | 0.506 |
| **DCN** | P07585 | 14.1 | (0.7) | [7] | 14.0 | (0.8) | [62] | 0.52 | 1 | 0.89 | -0.168 |
| **DDAH1** | O94760 | 13.5 | (0.7) | [7] | 12.6 | (1.6) | [44] | 0.007 | 1 | 0.53 | -0.916 |
| **DKK3** | F6SYF8 | 16.2 | (0.7) | [10] | 16.5 | (0.4) | [62] | 0.263 | 1 | 1.22 | 0.287 |
| **DPP7** | Q9UHL4 | 13.8 | (0.7) | [8] | 14.1 | (0.6) | [61] | 0.442 | 1 | 1.24 | 0.310 |
| **DPYSL2** | A0A1C7CYX9 | 14.9 | (0.7) | [5] | 14.3 | (1.0) | [55] | 0.202 | 1 | 0.68 | -0.556 |
| **DSC2** | Q02487 | 13.8 | (0.7) | [7] | 14.3 | (0.4) | [62] | 0.145 | 1 | 1.42 | 0.506 |
| **ECM1** | Q16610 | 15.0 | (0.7) | [10] | 14.9 | (0.4) | [62] | 0.816 | 1 | 0.97 | -0.044 |
| **ECM2** | O94769 | 14.5 | (0.7) | [8] | 14.7 | (0.5) | [62] | 0.351 | 1 | 1.15 | 0.202 |
| **EEF1A1** | P68104 | 14.4 | (0.7) | [9] | 13.6 | (1.1) | [21] | 0.11 | 1 | 0.57 | -0.811 |
| **EFCAB14** | O75071 | 14.4 | (0.7) | [6] | 14.0 | (0.6) | [56] | 0.242 | 1 | 0.77 | -0.377 |
| **EFEMP1** | A0A0U1RQV3 | 16.0 | (0.7) | [10] | 16.8 | (0.4) | [62] | 0.063 | 1 | 1.81 | 0.856 |
| **EFNA1** | P20827 | 14.0 | (0.7) | [7] | 13.5 | (1.2) | [56] | 0.377 | 1 | 0.73 | -0.454 |

| Name | Uniprot ID | Control | | | Communicating HC | | | P value | P adjusted | Fold change | Log2(fold change) |
|---|---|---|---|---|---|---|---|---|---|---|---|
| | | Mean | (SD) | [N] | Mean | (SD) | [N] | | | | |
| **ENDOD1** | O94919 | 15.1 | (0.7) | [10] | 15.7 | (0.3) | [62] | 0.092 | 1 | 1.54 | 0.623 |
| **ENO1** | P06733 | 15.8 | (0.7) | [10] | 15.1 | (0.7) | [61] | 0.008 | 1 | 0.60 | -0.737 |
| **ENO2** | P09104 | 14.7 | (0.7) | [9] | 14.6 | (0.7) | [61] | 0.602 | 1 | 0.92 | -0.120 |
| **ENPP2** | E7EUF1 | 16.4 | (0.7) | [10] | 17.4 | (0.6) | [62] | 0.080 | 1 | 2.01 | 1.007 |
| **ENPP4** | Q9Y6X5 | 13.1 | (0.7) | [8] | 13.0 | (0.5) | [60] | 0.852 | 1 | 0.93 | -0.105 |
| **EPDR1** | Q9UM22 | 14.1 | (0.7) | [6] | 13.8 | (0.6) | [58] | 0.521 | 1 | 0.82 | -0.286 |
| **EPHA4** | E9PG71 | 15.2 | (0.7) | [9] | 15.4 | (0.8) | [62] | 0.438 | 1 | 1.15 | 0.202 |
| **ERN1** | O75460 | 16.8 | (0.7) | [8] | 15.4 | (1.2) | [61] | 0.053 | 1 | 0.37 | -1.434 |
| **ESD** | H7BZT7 | 13.9 | (0.7) | [7] | 12.7 | (0.7) | [25] | 0.028 | 1 | 0.45 | -1.152 |
| **EXTL2** | Q9UBQ6 | 14.5 | (0.7) | [10] | 15.0 | (0.4) | [62] | 0.088 | 1 | 1.40 | 0.485 |
| **F10** | P00742 | 14.3 | (0.7) | [9] | 14.3 | (0.5) | [43] | 0.958 | 1 | 1.01 | 0.014 |
| **F12** | P00748 | 16.3 | (0.7) | [10] | 15.7 | (0.5) | [62] | 0.070 | 1 | 0.67 | -0.578 |
| **F2** | P00734 | 16.3 | (0.7) | [10] | 15.9 | (0.3) | [62] | 0.031 | 1 | 0.76 | -0.396 |
| **F5** | A0A0A0MRJ7 | 14.9 | (0.7) | [10] | 15.5 | (0.5) | [62] | 0.183 | 1 | 1.48 | 0.566 |
| **F9** | P00740 | 13.5 | (0.7) | [10] | 13.1 | (0.6) | [62] | 0.046 | 1 | 0.78 | -0.358 |
| **FABP5** | Q01469 | 13.2 | (0.7) | [5] | 11.1 | (4.0) | [11] | 0.131 | 1 | 0.24 | -2.059 |
| **FAM3C** | Q92520 | 15.3 | (0.7) | [10] | 15.5 | (0.3) | [62] | 0.346 | 1 | 1.22 | 0.287 |
| **FAT2** | Q9NYQ8 | 13.5 | (0.7) | [6] | 12.9 | (0.7) | [52] | 0.087 | 1 | 0.70 | -0.515 |
| **FBLN1** | P23142 | 16.2 | (0.7) | [10] | 16.6 | (0.4) | [62] | 0.122 | 1 | 1.33 | 0.411 |
| **FBLN5** | G3V4U0 | 14.3 | (0.7) | [8] | 15.1 | (0.8) | [62] | 0.013 | 1 | 1.76 | 0.816 |
| **FBLN7** | Q53RD9 | 12.7 | (0.7) | [5] | 12.6 | (0.4) | [56] | 0.691 | 1 | 0.91 | -0.136 |

| Name | Uniprot ID | Control Mean | (SD) | [N] | Communicating HC Mean | (SD) | [N] | P value | P adjusted | Fold change | $Log_2$(fold change) |
|---|---|---|---|---|---|---|---|---|---|---|---|
| **FCGBP** | Q9Y6R7 | 14.6 | (0.7) | [8] | 14.3 | (0.7) | [62] | 0.575 | 1 | 0.82 | -0.286 |
| **FCGR3A** | A0A1W2PQB1 | 14.3 | (0.7) | [10] | 14.5 | (0.8) | [62] | 0.513 | 1 | 1.18 | 0.239 |
| **FETUB** | Q9UGM5 | 14.6 | (0.7) | [9] | 14.0 | (0.7) | [62] | 0.092 | 1 | 0.69 | -0.535 |
| **FGA** | P02671 | 15.3 | (0.7) | [10] | 14.2 | (0.7) | [62] | 0.006 | 1 | 0.47 | -1.089 |
| **FGB** | P02675 | 16.5 | (0.7) | [10] | 15.4 | (0.9) | [62] | 0.027 | 1 | 0.48 | -1.059 |
| **FGFR2** | A0A0A0MR25 | 16.2 | (0.7) | [5] | 15.7 | (0.6) | [62] | 0.375 | 1 | 0.71 | -0.494 |
| **FGG** | P02679 | 15.5 | (0.7) | [10] | 14.8 | (0.8) | [62] | 0.127 | 1 | 0.61 | -0.713 |
| **FKBP1A** | P62942 | 15.0 | (0.7) | [9] | 14.2 | (0.6) | [15] | 0.044 | 1 | 0.58 | -0.786 |
| **FLNA** | P21333 | 13.5 | (0.7) | [8] | 12.2 | (1.7) | [16] | 0.018 | 1 | 0.38 | -1.396 |
| **FMOD** | Q06828 | 13.9 | (0.7) | [8] | 13.8 | (0.5) | [61] | 0.712 | 1 | 0.91 | -0.136 |
| **FN1** | P02751 | 16.1 | (0.7) | [10] | 16.0 | (0.3) | [62] | 0.418 | 1 | 0.93 | -0.105 |
| **FRRS1L** | Q9P0K9 | 10.7 | (0.7) | [6] | 10.2 | (2.0) | [50] | 0.356 | 1 | 0.69 | -0.535 |
| **FRZB** | Q92765 | 14.3 | (0.7) | [8] | 14.4 | (0.8) | [60] | 0.679 | 1 | 1.09 | 0.124 |
| **FSTL1** | Q12841 | 14.0 | (0.7) | [8] | 13.7 | (0.6) | [62] | 0.476 | 1 | 0.83 | -0.269 |
| **FSTL4** | Q6MZW2 | 13.8 | (0.7) | [7] | 13.1 | (0.7) | [58] | 0.12 | 1 | 0.65 | -0.621 |
| **FTH1** | P02794 | 12.0 | (0.7) | [7] | 11.7 | (0.9) | [33] | 0.423 | 1 | 0.84 | -0.252 |
| **FTL** | P02792 | 15.0 | (0.7) | [5] | 13.1 | (0.7) | [55] | 0.051 | 1 | 0.27 | -1.889 |
| **FUCA1** | P04066 | 14.1 | (0.7) | [8] | 14.6 | (0.7) | [62] | 0.199 | 1 | 1.33 | 0.411 |
| **FUCA2** | Q9BTY2 | 14.1 | (0.7) | [10] | 14.7 | (0.8) | [56] | 0.113 | 1 | 1.46 | 0.546 |
| **FXYD6** | Q9H0Q3 | 14.8 | (0.7) | [5] | 15.0 | (0.6) | [61] | 0.684 | 1 | 1.22 | 0.287 |
| **GALNT2** | Q10471 | 13.8 | (0.7) | [8] | 13.9 | (0.3) | [59] | 0.702 | 1 | 1.04 | 0.057 |

| Name | Uniprot ID | Control | | | Communicating HC | | | P value | P adjusted | Fold change | $Log_2$(fold change) |
|---|---|---|---|---|---|---|---|---|---|---|---|
| | | Mean | (SD) | [N] | Mean | (SD) | [N] | | | | |
| **GANAB** | Q14697 | 13.3 | (0.7) | [8] | 12.7 | (0.6) | [59] | 0.035 | 1 | 0.66 | -0.599 |
| **GAPDH** | P04406 | 15.6 | (0.7) | [10] | 14.7 | (0.7) | [62] | 0.034 | 1 | 0.54 | -0.889 |
| **GC** | P02774 | 16.7 | (0.7) | [10] | 16.6 | (0.3) | [62] | 0.143 | 1 | 0.92 | -0.120 |
| **GDA** | Q9Y2T3 | 14.2 | (0.7) | [6] | 13.7 | (0.7) | [60] | 0.106 | 1 | 0.75 | -0.415 |
| **GDI1** | P31150 | 14.8 | (0.7) | [7] | 14.5 | (0.8) | [40] | 0.253 | 1 | 0.85 | -0.234 |
| **GDI2** | P50395 | 14.2 | (0.7) | [9] | 13.5 | (0.6) | [61] | 0.013 | 1 | 0.63 | -0.667 |
| **GGH** | Q92820 | 14.8 | (0.7) | [10] | 14.6 | (0.5) | [61] | 0.518 | 1 | 0.89 | -0.168 |
| **GLOD4** | F6TLX2 | 15.0 | (0.7) | [7] | 15.4 | (1.4) | [38] | 0.387 | 1 | 1.28 | 0.356 |
| **GM2A** | P17900 | 15.9 | (0.7) | [10] | 16.4 | (0.4) | [62] | 0.134 | 1 | 1.40 | 0.485 |
| **GNPTG** | Q9UJJ9 | 15.6 | (0.7) | [8] | 15.5 | (0.4) | [62] | 0.615 | 1 | 0.91 | -0.136 |
| **GOLM1** | Q8NBJ4 | 13.5 | (0.7) | [8] | 13.5 | (0.6) | [61] | 0.902 | 1 | 1.01 | 0.014 |
| **GOT1** | P17174 | 15.3 | (0.7) | [10] | 14.9 | (0.3) | [62] | 0.149 | 1 | 0.76 | -0.396 |
| **GPC1** | P35052 | 13.6 | (0.7) | [5] | 13.2 | (1.6) | [45] | 0.329 | 1 | 0.78 | -0.358 |
| **GPLD1** | P80108 | 15.2 | (0.7) | [7] | 13.5 | (0.6) | [38] | 0.009 | 1 | 0.32 | -1.644 |
| **GPR37** | O15354 | 15.5 | (0.7) | [8] | 16.0 | (0.3) | [62] | 0.157 | 1 | 1.34 | 0.422 |
| **GPR37L1** | O60883 | 14.9 | (0.7) | [10] | 14.6 | (0.6) | [62] | 0.313 | 1 | 0.76 | -0.396 |
| **GPX3** | A0A087X1J7 | 16.2 | (0.7) | [10] | 15.9 | (0.4) | [62] | 0.358 | 1 | 0.82 | -0.286 |
| **GRIA4** | G3V164 | 12.5 | (0.7) | [6] | 12.8 | (0.5) | [57] | 0.478 | 1 | 1.27 | 0.345 |
| **GSN** | P06396 | 16.4 | (0.7) | [10] | 16.9 | (0.2) | [62] | 0.014 | 1 | 1.34 | 0.422 |
| **GSTO1** | P78417 | 15.2 | (0.7) | [7] | 13.8 | (0.6) | [62] | 0.003 | 1 | 0.39 | -1.358 |
| **GSTP1** | P09211 | 16.1 | (0.7) | [10] | 15.1 | (0.6) | [62] | 0.008 | 1 | 0.50 | -1.000 |

| Name | Uniprot ID | Control Mean | (SD) | [N] | Communicating HC Mean | (SD) | [N] | P value | P adjusted | Fold change | Log$_2$(fold change) |
|---|---|---|---|---|---|---|---|---|---|---|---|
| **HARS** | B3KWE1 | 20.9 | (0.7) | [5] | 21.3 | (0.4) | [24] | 0.368 | 1 | 1.33 | 0.411 |
| **HBD** | P02042 | 18.2 | (0.7) | [10] | 15.7 | (0.8) | [51] | 0.006 | 1 | 0.17 | -2.556 |
| **HDHD2** | K7ER15 | 14.4 | (0.7) | [6] | 12.9 | (0.6) | [25] | 0.02 | 1 | 0.36 | -1.474 |
| **HEXA** | H3BP20 | 14.5 | (0.7) | [8] | 14.6 | (0.6) | [62] | 0.788 | 1 | 1.06 | 0.084 |
| **HEXB** | P07686 | 14.4 | (0.7) | [8] | 14.7 | (0.5) | [62] | 0.528 | 1 | 1.23 | 0.299 |
| **HGFAC** | D6RAR4 | 14.4 | (0.7) | [10] | 14.0 | (0.5) | [48] | 0.097 | 1 | 0.78 | -0.358 |
| **HIST1H2BK** | O60814 | 14.7 | (0.7) | [6] | 14.3 | (1.0) | [40] | 0.39 | 1 | 0.76 | -0.396 |
| **HIST1H4A** | P62805 | 14.8 | (0.7) | [9] | 13.5 | (1.1) | [60] | 0.014 | 1 | 0.40 | -1.322 |
| **HLA-C** | A0A140T921 | 14.0 | (0.7) | [6] | 13.7 | (0.9) | [21] | 0.329 | 1 | 0.82 | -0.286 |
| **HP** | P00738 | 17.3 | (0.7) | [10] | 17.0 | (1.0) | [62] | 0.583 | 1 | 0.80 | -0.322 |
| **HPR** | P00739 | 16.1 | (0.7) | [10] | 14.5 | (0.8) | [62] | 0.005 | 1 | 0.33 | -1.599 |
| **HPRT1** | P00492 | 14.1 | (0.7) | [6] | 13.2 | (1.1) | [25] | 0.05 | 1 | 0.51 | -0.971 |
| **HPX** | P02790 | 17.9 | (0.7) | [10] | 18.1 | (0.3) | [62] | 0.323 | 1 | 1.17 | 0.227 |
| **HRG** | P04196 | 16.0 | (0.7) | [10] | 15.7 | (0.4) | [62] | 0.228 | 1 | 0.81 | -0.304 |
| **HSP90B1** | P14625 | 14.5 | (0.7) | [6] | 13.8 | (0.4) | [49] | 0.002 | 1 | 0.64 | -0.644 |
| **HSP90AA1** | P07900 | 14.4 | (0.7) | [8] | 13.8 | (0.7) | [24] | 0.039 | 1 | 0.64 | -0.644 |
| **HSPA13** | P48723 | 13.6 | (0.7) | [6] | 13.1 | (0.7) | [40] | 0.276 | 1 | 0.7 | -0.515 |
| **HSPA5** | P11021 | 14.5 | (0.7) | [9] | 14.2 | (0.3) | [62] | 0.155 | 1 | 0.86 | -0.218 |
| **HSPA8** | P11142 | 15.3 | (0.7) | [10] | 14.1 | (0.8) | [61] | 0.007 | 1 | 0.43 | -1.218 |
| **HSPB1** | P04792 | 16.4 | (0.7) | [5] | 13.9 | (1.6) | [11] | 0.013 | 1 | 0.18 | -2.474 |
| **HSPG2** | P98160 | 13.6 | (0.7) | [10] | 14.3 | (0.3) | [62] | 0.037 | 1 | 1.64 | 0.714 |

| Name | Uniprot ID | Control Mean | Control (SD) | Control [N] | Communicating HC Mean | Communicating HC (SD) | Communicating HC [N] | P value | P adjusted | Fold change | $Log_2$(fold change) |
|---|---|---|---|---|---|---|---|---|---|---|---|
| **HTRA1** | Q92743 | 14.1 | (0.7) | [9] | 14.7 | (0.4) | [62] | 0.132 | 1 | 1.50 | 0.585 |
| **HYOU1** | A0A087X054 | 14.1 | (0.7) | [6] | 14.3 | (0.3) | [60] | 0.491 | 1 | 1.12 | 0.163 |
| **ICAM5** | Q9UMF0 | 13.3 | (0.7) | [6] | 13.1 | (0.6) | [59] | 0.441 | 1 | 0.87 | -0.201 |
| **ICOSLG** | K4DIA0 | 15.4 | (0.7) | [10] | 16.0 | (0.7) | [62] | 0.12 | 1 | 1.52 | 0.604 |
| **IDS** | P22304 | 14.3 | (0.7) | [7] | 14.7 | (0.7) | [61] | 0.398 | 1 | 1.32 | 0.401 |
| **IGF2** | P01344 | 14.6 | (0.7) | [5] | 14.6 | (0.6) | [58] | 0.871 | 1 | 0.98 | -0.029 |
| **IGFBP2** | P18065 | 14.0 | (0.7) | [7] | 14.0 | (0.7) | [62] | 0.926 | 1 | 1.02 | 0.029 |
| **IGFBP5** | P24593 | 12.1 | (0.7) | [8] | 12.5 | (0.6) | [60] | 0.221 | 1 | 1.39 | 0.475 |
| **IGFBP6** | P24592 | 17.1 | (0.7) | [10] | 17.9 | (0.4) | [62] | 0.014 | 1 | 1.70 | 0.766 |
| **IGFBP7** | Q16270 | 15.9 | (0.7) | [10] | 17.3 | (0.7) | [62] | 0.054 | 1 | 2.64 | 1.401 |
| **IGHA1** | A0A286YEY1 | 17.6 | (0.7) | [10] | 17.1 | (0.9) | [62] | 0.196 | 1 | 0.70 | -0.515 |
| **IGHA2** | A0A286YEY5 | 17.7 | (0.7) | [10] | 17.1 | (0.8) | [62] | 0.063 | 1 | 0.65 | -0.621 |
| **IGHD** | A0A0A0MS09 | 15.8 | (0.7) | [8] | 14.1 | (0.9) | [35] | 0.004 | 1 | 0.32 | -1.644 |
| **IGHG1** | P01857 | 19.5 | (0.7) | [10] | 19.3 | (0.4) | [62] | 0.309 | 1 | 0.85 | -0.234 |
| **IGHG2** | P01859 | 19.4 | (0.7) | [10] | 19.4 | (0.5) | [62] | 0.736 | 1 | 0.95 | -0.074 |
| **IGHG3** | P01860 | 16.6 | (0.7) | [10] | 16.0 | (0.7) | [62] | 0.128 | 1 | 0.62 | -0.690 |
| **IGHG4** | A0A286YFJ8 | 15.9 | (0.7) | [10] | 15.9 | (1.0) | [61] | 0.981 | 1 | 1.00 | 0.000 |
| **IGHM** | A0A1B0GUU9 | 17.4 | (0.7) | [10] | 14.7 | (1.3) | [62] | 0.005 | 1 | 0.15 | -2.737 |
| **IGHV1-18** | A0A0C4DH31 | 14.2 | (0.7) | [6] | 14.3 | (0.5) | [28] | 0.627 | 1 | 1.11 | 0.151 |
| **IGHV1-2** | P23083 | 15.1 | (0.7) | [9] | 15.2 | (0.5) | [40] | 0.958 | 1 | 1.01 | 0.014 |
| **IGHV1-69** | P01742 | 15.0 | (0.7) | [5] | 14.4 | (2.1) | [17] | 0.236 | 1 | 0.63 | -0.667 |

| Name | Uniprot ID | Control Mean | Control (SD) | Control [N] | Communicating HC Mean | Communicating HC (SD) | Communicating HC [N] | P value | P adjusted | Fold change | $Log_2$(fold change) |
|---|---|---|---|---|---|---|---|---|---|---|---|
| **IGHV1OR15-1** | A0A075B7D0 | 17.0 | (0.7) | [9] | 16.6 | (1.3) | [61] | 0.449 | 1 | 0.74 | -0.434 |
| **IGHV2-26** | A0A0B4J1V2 | 13.1 | (0.7) | [8] | 13.0 | (0.8) | [61] | 0.542 | 1 | 0.90 | -0.152 |
| **IGHV2-5** | P01817 | 12.7 | (0.7) | [5] | 12.5 | (1.3) | [23] | 0.703 | 1 | 0.90 | -0.152 |
| **IGHV3-15** | A0A0B4J1V0 | 16.0 | (0.7) | [10] | 15.4 | (0.6) | [62] | 0.003 | 1 | 0.69 | -0.535 |
| **IGHV3-38** | A0A0C4DH36 | 15.4 | (0.7) | [10] | 15.0 | (0.6) | [58] | 0.127 | 1 | 0.77 | -0.377 |
| **IGHV3-49** | A0A0A0MS15 | 16.9 | (0.7) | [10] | 16.3 | (0.8) | [62] | 0.047 | 1 | 0.66 | -0.599 |
| **IGHV3-64D** | A0A0J9YX35 | 15.9 | (0.7) | [10] | 15.8 | (0.6) | [60] | 0.761 | 1 | 0.97 | -0.044 |
| **IGHV3-7** | P01780 | 17.0 | (0.7) | [10] | 16.8 | (0.4) | [62] | 0.025 | 1 | 0.85 | -0.234 |
| **IGHV3-72** | A0A0B4J1Y9 | 16.3 | (0.7) | [10] | 16.0 | (0.5) | [62] | 0.127 | 1 | 0.81 | -0.304 |
| **IGHV3OR16-12** | A0A075B7B8 | 13.1 | (0.7) | [7] | 13.0 | (0.5) | [38] | 0.487 | 1 | 0.91 | -0.136 |
| **IGHV3OR16-9** | A0A0B4J2B5 | 19.8 | (0.7) | [10] | 19.1 | (0.8) | [62] | 0.026 | 1 | 0.63 | -0.667 |
| **IGHV4-28** | A0A0C4DH34 | 11.0 | (0.7) | [6] | 9.5 | (0.7) | [20] | 0.007 | 1 | 0.35 | -1.515 |
| **IGHV4-34** | P06331 | 15.1 | (0.7) | [10] | 14.5 | (0.6) | [62] | 0.012 | 1 | 0.65 | -0.621 |
| **IGHV5-51** | A0A0C4DH38 | 15.8 | (0.7) | [10] | 15.5 | (0.5) | [62] | 0.058 | 1 | 0.80 | -0.322 |
| **IGKC** | P01834 | 20.1 | (0.7) | [10] | 19.6 | (0.5) | [62] | 0.1 | 1 | 0.72 | -0.474 |
| **IGKV1-12** | A0A0C4DH73 | 16.9 | (0.7) | [10] | 16.4 | (0.7) | [62] | 0.103 | 1 | 0.67 | -0.578 |
| **IGKV1-16** | P04430 | 14.4 | (0.7) | [7] | 14.4 | (0.7) | [30] | 0.859 | 1 | 0.97 | -0.044 |
| **IGKV1-17** | P01599 | 15.6 | (0.7) | [10] | 15.1 | (0.7) | [59] | 0.045 | 1 | 0.73 | -0.454 |
| **IGKV1-39** | P01597 | 19.0 | (0.7) | [5] | 19.7 | (0.8) | [28] | 0.244 | 1 | 1.61 | 0.687 |
| **IGKV1-5** | P01602 | 16.4 | (0.7) | [10] | 15.9 | (0.6) | [62] | 0.016 | 1 | 0.69 | -0.535 |
| **IGKV1-8** | A0A0C4DH67 | 16.3 | (0.7) | [10] | 15.8 | (0.5) | [62] | 0.213 | 1 | 0.70 | -0.515 |

| Name | Uniprot ID | Control | | | Communicating HC | | | P value | P adjusted | Fold change | $Log_2$(fold change) |
|---|---|---|---|---|---|---|---|---|---|---|---|
| | | Mean | (SD) | [N] | Mean | (SD) | [N] | | | | |
| **IGKV1D-13** | A0A0B4J2D9 | 15.6 | (0.7) | [6] | 15.1 | (1.0) | [28] | 0.545 | 1 | 0.72 | -0.474 |
| **IGKV1D-33** | P01593 | 17.7 | (0.7) | [10] | 16.8 | (0.8) | [62] | 0.029 | 1 | 0.53 | -0.916 |
| **IGKV1D-37** | A0A075B6S9 | 16.7 | (0.7) | [8] | 15.8 | (1.0) | [35] | 0.048 | 1 | 0.52 | -0.943 |
| **IGKV2-28** | A0A075B6P5 | 16.0 | (0.7) | [10] | 15.7 | (0.6) | [62] | 0.283 | 1 | 0.83 | -0.269 |
| **IGKV2-29** | A2NJV5 | 16.3 | (0.7) | [10] | 15.6 | (0.7) | [62] | 0.011 | 1 | 0.62 | -0.690 |
| **IGKV2-40** | A0A087WW87 | 13.5 | (0.7) | [8] | 13.5 | (0.6) | [28] | 0.749 | 1 | 1.06 | 0.084 |
| **IGKV2D-24** | A0A075B6R9 | 16.9 | (0.7) | [10] | 16.0 | (0.8) | [62] | 0.036 | 1 | 0.51 | -0.971 |
| **IGKV2D-29** | A0A075B6S2 | 13.9 | (0.7) | [6] | 14.1 | (0.7) | [43] | 0.588 | 1 | 1.11 | 0.151 |
| **IGKV3-15** | P01624 | 17.4 | (0.7) | [10] | 17.5 | (0.6) | [62] | 0.646 | 1 | 1.08 | 0.111 |
| **IGKV3-20** | P01619 | 18.3 | (0.7) | [10] | 17.8 | (0.6) | [62] | 0.072 | 1 | 0.70 | -0.515 |
| **IGKV3-7** | A0A075B6H7 | 17.3 | (0.7) | [9] | 18.4 | (1.1) | [57] | 0.059 | 1 | 2.08 | 1.057 |
| **IGKV3D-11** | A0A0A0MRZ8 | 17.6 | (0.7) | [10] | 17.1 | (0.6) | [62] | 0.027 | 1 | 0.74 | -0.434 |
| **IGKV3D-15** | A0A087WSY6 | 17.5 | (0.7) | [9] | 16.7 | (0.8) | [54] | 0.035 | 1 | 0.60 | -0.737 |
| **IGKV4-1** | P06312 | 17.4 | (0.7) | [10] | 17.0 | (0.5) | [62] | 0.039 | 1 | 0.77 | -0.377 |
| **IGLC3** | P0DOY3 | 19.9 | (0.7) | [10] | 19.7 | (0.6) | [62] | 0.161 | 1 | 0.85 | -0.234 |
| **IGLL5** | A0A0B4J231 | 18.0 | (0.7) | [10] | 17.5 | (0.5) | [62] | 0.052 | 1 | 0.68 | -0.556 |
| **IGLON5** | A6NGN9 | 13.8 | (0.7) | [5] | 13.9 | (0.5) | [17] | 0.569 | 1 | 1.11 | 0.151 |
| **IGLV1-36** | A0A0B4J1U3 | 15.5 | (0.7) | [5] | 15.4 | (1.0) | [12] | 0.874 | 1 | 0.94 | -0.089 |
| **IGLV1-47** | P01700 | 16.2 | (0.7) | [10] | 15.9 | (0.7) | [62] | 0.075 | 1 | 0.81 | -0.304 |
| **IGLV1-51** | P01701 | 16.0 | (0.7) | [8] | 14.5 | (1.7) | [28] | 0.003 | 1 | 0.36 | -1.474 |
| **IGLV3-10** | A0A075B6K4 | 15.5 | (0.7) | [8] | 14.7 | (0.6) | [47] | 0.013 | 1 | 0.58 | -0.786 |

| Name | Uniprot ID | Control Mean | Control (SD) | Control [N] | Communicating HC Mean | Communicating HC (SD) | Communicating HC [N] | P value | P adjusted | Fold change | $Log_2$(fold change) |
|---|---|---|---|---|---|---|---|---|---|---|---|
| **IGLV3-19** | P01714 | 14.3 | (0.7) | [9] | 14.3 | (0.7) | [46] | 0.946 | 1 | 1.01 | 0.014 |
| **IGLV3-21** | P80748 | 15.2 | (0.7) | [9] | 14.7 | (1.1) | [55] | 0.244 | 1 | 0.73 | -0.454 |
| **IGLV3-25** | P01717 | 14.3 | (0.7) | [10] | 14.3 | (0.6) | [61] | 0.992 | 1 | 1.00 | 0.000 |
| **IGLV3-9** | A0A075B6K5 | 15.2 | (0.7) | [10] | 14.4 | (0.6) | [62] | 0.082 | 1 | 0.56 | -0.837 |
| **IGLV6-57** | P01721 | 14.6 | (0.7) | [8] | 13.6 | (0.7) | [46] | 0.008 | 1 | 0.51 | -0.971 |
| **IGLV7-46** | A0A075B6I9 | 15.5 | (0.7) | [7] | 15.2 | (1.1) | [36] | 0.301 | 1 | 0.83 | -0.269 |
| **IGLV8-61** | A0A075B6I0 | 15.8 | (0.7) | [9] | 14.7 | (0.8) | [45] | 0.005 | 1 | 0.49 | -1.029 |
| **IGSF21** | Q96ID5 | 14.9 | (0.7) | [6] | 14.2 | (1.5) | [55] | 0.02 | 1 | 0.61 | -0.713 |
| **IGSF8** | Q969P0 | 15.4 | (0.7) | [10] | 16.1 | (0.4) | [62] | 0.106 | 1 | 1.60 | 0.678 |
| **IL31RA** | Q8NI17 | 22.5 | (0.7) | [7] | 24.4 | (0.6) | [54] | 0.003 | 1 | 3.74 | 1.903 |
| **IL6ST** | P40189 | 14.5 | (0.7) | [8] | 14.6 | (0.4) | [61] | 0.678 | 1 | 1.07 | 0.098 |
| **IMPAD1** | Q9NX62 | 14.1 | (0.7) | [7] | 13.9 | (0.5) | [62] | 0.481 | 1 | 0.86 | -0.218 |
| **ISLR** | O14498 | 14.9 | (0.7) | [9] | 15.0 | (0.3) | [62] | 0.851 | 1 | 1.03 | 0.043 |
| **ISLR2** | Q6UXK2 | 13.4 | (0.7) | [5] | 14.7 | (2.3) | [37] | 0.341 | 1 | 2.38 | 1.251 |
| **ITIH1** | P19827 | 16.0 | (0.7) | [10] | 14.8 | (0.5) | [62] | 0.003 | 1 | 0.44 | -1.184 |
| **ITIH2** | P19823 | 16.0 | (0.7) | [10] | 15.0 | (0.4) | [62] | 0.014 | 1 | 0.52 | -0.943 |
| **ITIH3** | Q06033 | 14.8 | (0.7) | [9] | 13.6 | (0.7) | [53] | 0.005 | 1 | 0.44 | -1.184 |
| **ITIH5** | C9J2H1 | 13.3 | (0.7) | [8] | 13.8 | (0.3) | [61] | 0.035 | 1 | 1.41 | 0.496 |
| **ITM2B** | Q9Y287 | 14.1 | (0.7) | [8] | 13.6 | (1.8) | [62] | 0.431 | 1 | 0.74 | -0.434 |
| **ITPR2** | Q14571 | 18.1 | (0.7) | [10] | 18.9 | (0.8) | [62] | 0.047 | 1 | 1.74 | 0.799 |
| **JCHAIN** | D6RD17 | 16.6 | (0.7) | [9] | 14.4 | (1.2) | [57] | 0.005 | 1 | 0.21 | -2.252 |

| Name | Uniprot ID | Control | | | Communicating HC | | | P value | P adjusted | Fold change | $Log_2$(fold change) |
|---|---|---|---|---|---|---|---|---|---|---|---|
| | | Mean | (SD) | [N] | Mean | (SD) | [N] | | | | |
| **KIAA1549L** | H0YDE5 | 14.4 | (0.7) | [10] | 14.4 | (0.5) | [62] | 0.938 | 1 | 0.98 | -0.029 |
| **KLK6** | Q92876 | 16.4 | (0.7) | [10] | 17.6 | (0.5) | [62] | 0.031 | 1 | 2.25 | 1.170 |
| **KLKB1** | H0YAC1 | 14.6 | (0.7) | [10] | 13.5 | (0.5) | [62] | 0.011 | 1 | 0.48 | -1.059 |
| **KNG1** | P01042 | 17.1 | (0.7) | [10] | 16.6 | (0.4) | [62] | 0.111 | 1 | 0.72 | -0.474 |
| **KRT1** | P04264 | 14.9 | (0.7) | [8] | 15.4 | (1.9) | [61] | 0.357 | 1 | 1.47 | 0.556 |
| **KRT10** | P13645 | 14.1 | (0.7) | [5] | 13.9 | (1.5) | [47] | 0.435 | 1 | 0.83 | -0.269 |
| **KRT2** | P35908 | 13.7 | (0.7) | [9] | 14.9 | (1.7) | [51] | 0.117 | 1 | 2.20 | 1.138 |
| **KRT6A** | P02538 | 13.8 | (0.7) | [5] | 13.4 | (1.8) | [45] | 0.585 | 1 | 0.79 | -0.340 |
| **KRT77** | Q7Z794 | 14.7 | (0.7) | [6] | 15.4 | (1.8) | [45] | 0.176 | 1 | 1.63 | 0.705 |
| **KRT9** | P35527 | 14.4 | (0.7) | [7] | 14.3 | (1.6) | [49] | 0.96 | 1 | 0.98 | -0.029 |
| **L1CAM** | P32004 | 13.5 | (0.7) | [6] | 12.6 | (0.7) | [61] | 0.017 | 1 | 0.55 | -0.862 |
| **LAMA2** | A0A087WX80 | 11.5 | (0.7) | [6] | 11.1 | (1.3) | [44] | 0.448 | 1 | 0.78 | -0.358 |
| **LAMC1** | P11047 | 14.7 | (0.7) | [5] | 14.3 | (1.7) | [45] | 0.157 | 1 | 0.75 | -0.415 |
| **LAMP1** | P11279 | 13.8 | (0.7) | [5] | 13.0 | (0.5) | [53] | 0.036 | 1 | 0.6 | -0.737 |
| **LAMP2** | P13473 | 15.5 | (0.7) | [10] | 16.2 | (0.6) | [62] | 0.104 | 1 | 1.56 | 0.642 |
| **LBP** | P18428 | 14.0 | (0.7) | [7] | 13.4 | (0.6) | [54] | 0.014 | 1 | 0.66 | -0.599 |
| **LCAT** | P04180 | 14.9 | (0.7) | [8] | 15.0 | (0.6) | [62] | 0.677 | 1 | 1.05 | 0.070 |
| **LCP1** | P13796 | 14.6 | (0.7) | [7] | 13.6 | (0.6) | [42] | 0.006 | 1 | 0.49 | -1.029 |
| **LDHA** | P00338 | 15.0 | (0.7) | [10] | 13.5 | (0.6) | [62] | 0.008 | 1 | 0.36 | -1.474 |
| **LDHB** | P07195 | 15.4 | (0.7) | [10] | 14.8 | (0.4) | [62] | 0.027 | 1 | 0.64 | -0.644 |
| **LGALS1** | P09382 | 14.8 | (0.7) | [10] | 14.3 | (0.5) | [51] | 0.045 | 1 | 0.73 | -0.454 |

| Name | Uniprot ID | Control Mean | (SD) | [N] | Communicating HC Mean | (SD) | [N] | P value | P adjusted | Fold change | $Log_2$(fold change) |
|---|---|---|---|---|---|---|---|---|---|---|---|
| **LGALS3** | P17931 | 13.2 | (0.7) | [5] | 12.0 | (0.6) | [18] | 0.043 | 1 | 0.44 | -1.184 |
| **LGALS3BP** | Q08380 | 16.3 | (0.7) | [10] | 17.0 | (0.3) | [62] | 0.123 | 1 | 1.53 | 0.614 |
| **LIAS** | A0A1W2PNQ5 | 14.5 | (0.7) | [7] | 14.4 | (0.7) | [41] | 0.902 | 1 | 0.96 | -0.059 |
| **LMAN2** | D6RBV2 | 14.9 | (0.7) | [9] | 15.4 | (0.4) | [62] | 0.031 | 1 | 1.45 | 0.536 |
| **LRG1** | P02750 | 16.6 | (0.7) | [10] | 16.3 | (0.4) | [62] | 0.004 | 1 | 0.80 | -0.322 |
| **LRP1** | Q07954 | 13.8 | (0.7) | [7] | 13.7 | (0.5) | [62] | 0.674 | 1 | 0.94 | -0.089 |
| **LRRC4B** | Q9NT99 | 14.6 | (0.7) | [8] | 14.9 | (0.4) | [62] | 0.367 | 1 | 1.20 | 0.263 |
| **LSAMP** | H3BLU2 | 15.1 | (0.7) | [10] | 15.4 | (0.4) | [62] | 0.382 | 1 | 1.19 | 0.251 |
| **LTBP2** | G3V3X5 | 12.8 | (0.7) | [6] | 12.6 | (0.5) | [59] | 0.214 | 1 | 0.87 | -0.201 |
| **LTF** | E7EQB2 | 14.6 | (0.7) | [8] | 15.2 | (1.2) | [58] | 0.53 | 1 | 1.48 | 0.566 |
| **LUM** | P51884 | 15.8 | (0.7) | [10] | 15.6 | (0.3) | [62] | 0.218 | 1 | 0.85 | -0.234 |
| **LY6H** | O94772 | 11.0 | (0.7) | [6] | 11.7 | (0.8) | [57] | 0.109 | 1 | 1.60 | 0.678 |
| **LYVE1** | Q9Y5Y7 | 14.8 | (0.7) | [10] | 14.6 | (0.5) | [62] | 0.476 | 1 | 0.88 | -0.184 |
| **LYZ** | A0A0B4J259 | 16.3 | (0.7) | [10] | 16.9 | (0.6) | [62] | 0.195 | 1 | 1.46 | 0.546 |
| **MAN1A1** | P33908 | 14.3 | (0.7) | [10] | 14.2 | (0.3) | [62] | 0.725 | 1 | 0.96 | -0.059 |
| **MAN1C1** | Q9NR34 | 13.4 | (0.7) | [7] | 13.7 | (0.5) | [62] | 0.31 | 1 | 1.26 | 0.333 |
| **MAN2A2** | P49641 | 14.1 | (0.7) | [8] | 14.1 | (0.4) | [62] | 0.903 | 1 | 1.02 | 0.029 |
| **MANBA** | O00462 | 14.0 | (0.7) | [5] | 13.3 | (0.5) | [53] | 0.165 | 1 | 0.61 | -0.713 |
| **MARCKS** | P29966 | 12.3 | (0.7) | [8] | 11.7 | (0.6) | [61] | 0.029 | 1 | 0.68 | -0.556 |
| **MASP1** | P48740 | 13.4 | (0.7) | [10] | 13.9 | (0.8) | [42] | 0.027 | 1 | 1.39 | 0.475 |
| **MCAM** | P43121 | 14.7 | (0.7) | [10] | 14.9 | (0.6) | [62] | 0.392 | 1 | 1.15 | 0.202 |

| Name | Uniprot ID | Control Mean | Control (SD) | Control [N] | Communicating HC Mean | Communicating HC (SD) | Communicating HC [N] | P value | P adjusted | Fold change | $Log_2$(fold change) |
|---|---|---|---|---|---|---|---|---|---|---|---|
| **MDH1** | P40925 | 15.9 | (0.7) | [10] | 15.4 | (0.4) | [62] | 0.031 | 1 | 0.69 | -0.535 |
| **MDH2** | P40926 | 14.6 | (0.7) | [5] | 13.8 | (0.8) | [26] | 0.164 | 1 | 0.58 | -0.786 |
| **MEGF8** | Q7Z7M0 | 14.3 | (0.7) | [10] | 14.4 | (0.4) | [61] | 0.508 | 1 | 1.11 | 0.151 |
| **MFAP4** | K7ES70 | 14.5 | (0.7) | [7] | 14.5 | (0.5) | [61] | 0.936 | 1 | 0.98 | -0.029 |
| **MGP** | P08493 | 15.0 | (0.7) | [10] | 15.6 | (0.5) | [62] | 0.116 | 1 | 1.54 | 0.623 |
| **MIF** | P14174 | 16.1 | (0.7) | [9] | 15.3 | (0.5) | [55] | 0.011 | 1 | 0.57 | -0.811 |
| **MINPP1** | Q9UNW1 | 15.2 | (0.7) | [6] | 14.5 | (1.2) | [36] | 0.007 | 1 | 0.61 | -0.713 |
| **MMP2** | P08253 | 14.8 | (0.7) | [10] | 15.3 | (0.3) | [62] | 0.073 | 1 | 1.45 | 0.536 |
| **MOG** | A0A0G2JHA9 | 14.0 | (0.7) | [8] | 14.4 | (0.7) | [62] | 0.282 | 1 | 1.34 | 0.422 |
| **MRC2** | Q9UBG0 | 13.0 | (0.7) | [5] | 12.6 | (1.2) | [19] | 0.111 | 1 | 0.73 | -0.454 |
| **MSN** | P26038 | 13.1 | (0.7) | [8] | 12.1 | (1.1) | [40] | 0.003 | 1 | 0.5 | -1.000 |
| **MST1** | G3XAK1 | 13.8 | (0.7) | [6] | 12.7 | (0.6) | [45] | 0.013 | 1 | 0.47 | -1.089 |
| **NBL1** | A0A087WTY6 | 17.7 | (0.7) | [10] | 18.3 | (0.6) | [62] | 0.018 | 1 | 1.58 | 0.660 |
| **NCAM1** | P13591 | 15.6 | (0.7) | [10] | 16.1 | (0.3) | [62] | 0.054 | 1 | 1.39 | 0.475 |
| **NCAM2** | H9KV31 | 14.9 | (0.7) | [10] | 15.6 | (0.4) | [62] | 0.088 | 1 | 1.54 | 0.623 |
| **NCAN** | O14594 | 15.0 | (0.7) | [10] | 15.4 | (0.4) | [62] | 0.253 | 1 | 1.27 | 0.345 |
| **NDRG2** | Q9UN36-2 | 13.6 | (0.7) | [8] | 13.7 | (0.8) | [54] | 0.56 | 1 | 1.08 | 0.111 |
| **NECTIN1** | Q15223 | 14.3 | (0.7) | [7] | 14.4 | (0.3) | [62] | 0.514 | 1 | 1.12 | 0.163 |
| **NEGR1** | Q7Z3B1 | 15.6 | (0.7) | [10] | 15.7 | (0.7) | [62] | 0.844 | 1 | 1.05 | 0.070 |
| **NELL2** | F8VVB6 | 15.2 | (0.7) | [10] | 15.7 | (0.4) | [62] | 0.136 | 1 | 1.40 | 0.485 |
| **NEO1** | Q92859 | 14.6 | (0.7) | [10] | 15.1 | (0.5) | [62] | 0.134 | 1 | 1.45 | 0.536 |

| Name | Uniprot ID | Control | | | Communicating HC | | | P value | P adjusted | Fold change | $Log_2$(fold change) |
|---|---|---|---|---|---|---|---|---|---|---|---|
| | | Mean | (SD) | [N] | Mean | (SD) | [N] | | | | |
| **NFASC** | O94856 | 14.6 | (0.7) | [9] | 15.0 | (0.4) | [62] | 0.369 | 1 | 1.24 | 0.310 |
| **NID1** | P14543 | 13.9 | (0.7) | [5] | 13.5 | (0.3) | [61] | 0.111 | 1 | 0.79 | -0.340 |
| **NID2** | Q14112 | 14.5 | (0.7) | [8] | 14.0 | (0.5) | [56] | 0.1 | 1 | 0.72 | -0.474 |
| **NLGN4X** | A0A0A0MTH0 | 11.8 | (0.7) | [5] | 12.0 | (1.5) | [42] | 0.732 | 1 | 1.13 | 0.176 |
| **NPC2** | E7EMS2 | 16.0 | (0.7) | [10] | 16.9 | (0.4) | [62] | 0.058 | 1 | 1.84 | 0.880 |
| **NPDC1** | Q5SPY9 | 13.6 | (0.7) | [10] | 13.8 | (0.5) | [62] | 0.316 | 1 | 1.15 | 0.202 |
| **NPPC** | P23582 | 13.6 | (0.7) | [10] | 14.1 | (0.5) | [62] | 0.045 | 1 | 1.4 | 0.485 |
| **NPTX1** | Q15818 | 15.8 | (0.7) | [10] | 16.0 | (0.7) | [62] | 0.423 | 1 | 1.19 | 0.251 |
| **NPTXR** | A0A1X7SBT7 | 15.4 | (0.7) | [10] | 15.6 | (0.5) | [62] | 0.582 | 1 | 1.15 | 0.202 |
| **NPY** | P01303 | 14.2 | (0.7) | [6] | 14.5 | (1.1) | [59] | 0.687 | 1 | 1.18 | 0.239 |
| **NRCAM** | C9JYY6 | 15.8 | (0.7) | [10] | 16.2 | (0.4) | [62] | 0.154 | 1 | 1.33 | 0.411 |
| **NRN1** | A0A087WWT2 | 15.1 | (0.7) | [8] | 15.5 | (0.3) | [61] | 0.074 | 1 | 1.31 | 0.390 |
| **NRP1** | E7EX60 | 13.0 | (0.7) | [6] | 12.8 | (0.5) | [60] | 0.537 | 1 | 0.88 | -0.184 |
| **NRXN1** | A0A0D9SEP4 | 13.9 | (0.7) | [8] | 14.1 | (0.3) | [61] | 0.501 | 1 | 1.15 | 0.202 |
| **NRXN2** | G5E9G7 | 14.7 | (0.7) | [10] | 15.0 | (0.6) | [62] | 0.296 | 1 | 1.27 | 0.345 |
| **NRXN3** | A0A0U1RQC5 | 14.4 | (0.7) | [10] | 14.5 | (0.6) | [62] | 0.72 | 1 | 1.08 | 0.111 |
| **NSF** | I3L0N3 | 13.1 | (0.7) | [7] | 12.9 | (1.2) | [28] | 0.669 | 1 | 0.90 | -0.152 |
| **NTM** | Q9P121-4 | 14.9 | (0.7) | [10] | 15.2 | (0.7) | [62] | 0.216 | 1 | 1.28 | 0.356 |
| **NTRK2** | Q16620 | 15.2 | (0.7) | [5] | 15.2 | (0.5) | [44] | 0.874 | 1 | 0.97 | -0.044 |
| **NUCB1** | Q02818 | 13.5 | (0.7) | [10] | 14.0 | 0.3) | [62] | 0.184 | 1 | 1.41 | 0.496 |
| **OAF** | Q86UD1 | 14.6 | (0.7) | [10] | 14.9 | (0.3) | [62] | 0.13 | 1 | 1.24 | 0.310 |

| Name | Uniprot ID | Control Mean | (SD) | [N] | Communicating HC Mean | (SD) | [N] | P value | P adjusted | Fold change | $Log_2$(fold change) |
|---|---|---|---|---|---|---|---|---|---|---|---|
| **OGN** | P20774 | 15.8 | (0.7) | [10] | 16.2 | (0.4) | [62] | 0.311 | 1 | 1.28 | 0.356 |
| **OMD** | Q99983 | 13.5 | (0.7) | [6] | 13.8 | (0.7) | [61] | 0.268 | 1 | 1.28 | 0.356 |
| **OMG** | P23515 | 15.4 | (0.7) | [10] | 16.1 | (0.4) | [62] | 0.057 | 1 | 1.70 | 0.766 |
| **OPCML** | Q14982 | 14.8 | (0.7) | [9] | 14.4 | (1.0) | [38] | 0.229 | 1 | 0.76 | -0.396 |
| **ORM1** | P02763 | 18.8 | (0.7) | [10] | 19.0 | (0.3) | [62] | 0.415 | 1 | 1.15 | 0.202 |
| **ORM2** | P19652 | 17.3 | (0.7) | [10] | 17.6 | (0.4) | [62] | 0.191 | 1 | 1.16 | 0.214 |
| **PAM** | P19021 | 15.2 | (0.7) | [10] | 15.4 | (0.3) | [62] | 0.485 | 1 | 1.10 | 0.138 |
| **PARK7** | Q99497 | 14.1 | (0.7) | [7] | 13.0 | (1.0) | [31] | 0.027 | 1 | 0.47 | -1.089 |
| **PCDH1** | Q08174 | 13.3 | (0.7) | [9] | 13.4 | (0.5) | [58] | 0.555 | 1 | 1.07 | 0.098 |
| **PCDH10** | Q9P2E7 | 11.1 | (0.7) | [5] | 10.8 | (0.7) | [24] | 0.327 | 1 | 0.76 | -0.396 |
| **PCDH7** | O60245 | 13.2 | (0.7) | [5] | 12.6 | (0.7) | [44] | 0.12 | 1 | 0.66 | -0.599 |
| **PCMT1** | A0A0A0MRJ6 | 14.1 | (0.7) | [5] | 13.6 | (1.6) | [42] | 0.11 | 1 | 0.69 | -0.535 |
| **PCOLCE** | Q15113 | 15.8 | (0.7) | [10] | 16.3 | (0.3) | [62] | 0.087 | 1 | 1.44 | 0.526 |
| **PCSK1N** | Q9UHG2 | 15.7 | (0.7) | [10] | 15.7 | (0.5) | [62] | 0.992 | 1 | 1.00 | 0.000 |
| **PCSK9** | Q8NBP7 | 12.0 | (0.7) | [5] | 11.1 | (0.5) | [18] | 0.122 | 1 | 0.53 | -0.916 |
| **PDGFA** | A0A0A0MSC4 | 15.4 | (0.7) | [5] | 15.4 | (1.3) | [43] | 0.862 | 1 | 1.05 | 0.070 |
| **PDGFB** | A9UJN9 | 13.8 | (0.7) | [6] | 14.1 | (0.7) | [49] | 0.283 | 1 | 1.24 | 0.310 |
| **PEA15** | Q15121 | 14.5 | (0.7) | [6] | 13.6 | (0.6) | [50] | 0.028 | 1 | 0.54 | -0.889 |
| **PEBP1** | P30086 | 16.2 | (0.7) | [10] | 16.1 | (0.4) | [62] | 0.842 | 1 | 0.97 | -0.044 |
| **PEBP4** | Q96S96 | 15.6 | (0.7) | [9] | 16.4 | (0.5) | [62] | 0.037 | 1 | 1.68 | 0.748 |
| **PENK** | P01210 | 14.9 | (0.7) | [10] | 15.6 | (0.5) | [62] | 0.04 | 1 | 1.57 | 0.651 |

| Name | Uniprot ID | Control Mean | (SD) | [N] | Communicating HC Mean | (SD) | [N] | P value | P adjusted | Fold change | $Log_2$(fold change) |
|---|---|---|---|---|---|---|---|---|---|---|---|
| **PEPD** | P12955 | 12.8 | (0.7) | [9] | 12.5 | (0.4) | [58] | 0.339 | 1 | 0.80 | -0.322 |
| **PFN1** | P07737 | 15.3 | (0.7) | [9] | 15.1 | (0.7) | [44] | 0.579 | 1 | 0.89 | -0.168 |
| **PGAM1** | P18669 | 14.9 | (0.7) | [10] | 14.6 | (0.7) | [62] | 0.088 | 1 | 0.76 | -0.396 |
| **PGLYRP2** | Q96PD5 | 14.8 | (0.7) | [10] | 14.5 | (0.4) | [62] | 0.320 | 1 | 0.86 | -0.218 |
| **PI16** | Q6UXB8 | 13.3 | (0.7) | [10] | 13.8 | (0.6) | [62] | 0.045 | 1 | 1.39 | 0.475 |
| **PIK3IP1** | Q96FE7-4 | 14.9 | (0.7) | [8] | 15.3 | (0.4) | [62] | 0.197 | 1 | 1.32 | 0.401 |
| **PKM** | P14618 | 15.0 | (0.7) | [10] | 14.6 | (0.4) | [62] | 0.252 | 1 | 0.77 | -0.377 |
| **PLD3** | Q8IV08 | 15.1 | (0.7) | [7] | 14.9 | (0.6) | [62] | 0.422 | 1 | 0.87 | -0.201 |
| **PLG** | P00747 | 16.4 | (0.7) | [10] | 15.9 | (0.3) | [62] | 0.067 | 1 | 0.71 | -0.494 |
| **PLOD1** | Q02809 | 12.9 | (0.7) | [5] | 12.9 | (0.5) | [49] | 0.911 | 1 | 1.02 | 0.029 |
| **PLTP** | P55058 | 15.4 | (0.7) | [10] | 16.0 | (0.3) | [62] | 0.056 | 1 | 1.52 | 0.604 |
| **PLXDC2** | Q6UX71 | 14.7 | (0.7) | [10] | 15.3 | (0.4) | [62] | 0.089 | 1 | 1.43 | 0.516 |
| **PLXNB2** | O15031 | 14.1 | (0.7) | [6] | 14.0 | (0.5) | [59] | 0.935 | 1 | 0.98 | -0.029 |
| **PMFBP1** | G3V1Q7 | 16.2 | (0.7) | [7] | 16.8 | (0.3) | [49] | 0.008 | 1 | 1.59 | 0.669 |
| **PNP** | P00491 | 14.9 | (0.7) | [5] | 14.5 | (0.9) | [12] | 0.270 | 1 | 0.78 | -0.358 |
| **PODXL2** | Q9NZ53 | 12.6 | (0.7) | [6] | 12.6 | (0.9) | [54] | 0.879 | 1 | 0.97 | -0.044 |
| **POMGNT1** | Q8WZA1 | 14.3 | (0.7) | [7] | 14.3 | (0.5) | [62] | 0.806 | 1 | 1.03 | 0.043 |
| **PON1** | P27169 | 16.2 | (0.7) | [9] | 15.2 | (0.6) | [62] | 0.013 | 1 | 0.50 | -1.000 |
| **POSTN** | B1ALD9 | 14.0 | (0.7) | [6] | 13.9 | (1.3) | [18] | 0.670 | 1 | 0.91 | -0.136 |
| **PPIA** | P62937 | 16.3 | (0.7) | [10] | 15.2 | (0.6) | [62] | 0.004 | 1 | 0.46 | -1.120 |
| **PPIB** | P23284 | 15.3 | (0.7) | [10] | 15.1 | (0.5) | [62] | 0.755 | 1 | 0.93 | -0.105 |

| Name | Uniprot ID | Control Mean | Control (SD) | Control [N] | Communicating HC Mean | Communicating HC (SD) | Communicating HC [N] | P value | P adjusted | Fold change | $Log_2$(fold change) |
|---|---|---|---|---|---|---|---|---|---|---|---|
| **PPIC** | P45877 | 15.0 | (0.7) | [7] | 14.8 | (0.3) | [60] | 0.320 | 1 | 0.86 | -0.218 |
| **PRCP** | P42785 | 15.2 | (0.7) | [8] | 15.2 | (0.6) | [62] | 0.954 | 1 | 0.99 | -0.014 |
| **PRDX1** | Q06830 | 17.1 | (0.7) | [10] | 15.6 | (0.9) | [60] | 0.015 | 1 | 0.37 | -1.434 |
| **PRDX6** | P30041 | 15.8 | (0.7) | [10] | 14.5 | (1.0) | [60] | 0.022 | 1 | 0.40 | -1.322 |
| **PRELP** | P51888 | 14.3 | (0.7) | [8] | 13.8 | (0.4) | [61] | 0.135 | 1 | 0.72 | -0.474 |
| **PRG4** | A0A0U1RR20 | 12.9 | (0.7) | [7] | 12.6 | (0.8) | [40] | 0.407 | 1 | 0.79 | -0.340 |
| **PRKCSH** | K7ELL7 | 13.7 | (0.7) | [6] | 15.1 | (0.5) | [50] | 0.024 | 1 | 2.61 | 1.384 |
| **PRNP** | A2A2V1 | 13.4 | (0.7) | [10] | 14.1 | (0.5) | [62] | 0.024 | 1 | 1.59 | 0.669 |
| **PROC** | E7END6 | 13.6 | (0.7) | [9] | 13.3 | (0.6) | [58] | 0.075 | 1 | 0.83 | -0.269 |
| **PROCR** | Q9UNN8 | 14.5 | (0.7) | [10] | 14.4 | (0.8) | [62] | 0.801 | 1 | 0.96 | -0.059 |
| **PROS1** | P07225 | 15.3 | (0.7) | [10] | 15.3 | (0.3) | [62] | 0.808 | 1 | 0.97 | -0.044 |
| **PROZ** | P22891 | 14.0 | (0.7) | [8] | 13.1 | (0.7) | [32] | 0.026 | 1 | 0.51 | -0.971 |
| **PRSS1** | E7EQ64 | 15.9 | (0.7) | [7] | 16.1 | (0.6) | [31] | 0.778 | 1 | 1.13 | 0.176 |
| **PRSS3** | B1AN99 | 18.5 | (0.7) | [5] | 20.5 | (1.6) | [20] | 0.033 | 1 | 3.98 | 1.993 |
| **PSAP** | C9JIZ6 | 14.3 | (0.7) | [8] | 14.2 | (0.8) | [62] | 0.880 | 1 | 0.96 | -0.059 |
| **PSAT1** | Q9Y617 | 13.5 | (0.7) | [6] | 13.8 | (0.7) | [52] | 0.560 | 1 | 1.19 | 0.251 |
| **PTGDS** | P41222 | 20.1 | (0.7) | [10] | 20.9 | (0.3) | [62] | 0.006 | 1 | 1.73 | 0.791 |
| **PTPRD** | P23468 | 14.3 | (0.7) | [9] | 14.6 | (0.5) | [62] | 0.299 | 1 | 1.26 | 0.333 |
| **PTPRF** | P10586 | 13.8 | (0.7) | [6] | 13.4 | (1.3) | [46] | 0.306 | 1 | 0.78 | -0.358 |
| **PTPRG** | P23470 | 15.0 | (0.7) | [10] | 15.2 | (0.3) | [62] | 0.377 | 1 | 1.17 | 0.227 |
| **PTPRN** | Q16849 | 12.9 | (0.7) | [5] | 11.6 | (2.2) | [26] | 0.037 | 1 | 0.42 | -1.252 |

| Name | Uniprot ID | Control | | | Communicating HC | | | P value | P adjusted | Fold change | $Log_2$(fold change) |
|---|---|---|---|---|---|---|---|---|---|---|---|
| | | Mean | (SD) | [N] | Mean | (SD) | [N] | | | | |
| **PTPRN2** | Q92932 | 14.8 | (0.7) | [10] | 15.5 | (0.5) | [62] | 0.008 | 1 | 1.56 | 0.642 |
| **PTPRS** | Q13332 | 14.2 | (0.7) | [10] | 14.7 | (0.3) | [62] | 0.123 | 1 | 1.45 | 0.536 |
| **PTPRZ1** | P23471 | 14.7 | (0.7) | [10] | 15.3 | (0.5) | [62] | 0.104 | 1 | 1.52 | 0.604 |
| **PVALB** | B8ZZ19 | 14.1 | (0.7) | [5] | 13.4 | (0.7) | [37] | 0.002 | 1 | 0.60 | -0.737 |
| **QDPR** | P09417 | 14.8 | (0.7) | [8] | 14.6 | (0.8) | [59] | 0.607 | 1 | 0.86 | -0.218 |
| **QPCT** | Q16769 | 13.7 | (0.7) | [10] | 13.9 | (0.6) | [62] | 0.273 | 1 | 1.17 | 0.227 |
| **QSOX1** | O00391 | 14.1 | (0.7) | [10] | 14.4 | (0.3) | [62] | 0.159 | 1 | 1.24 | 0.310 |
| **RARRES2** | Q99969 | 15.0 | (0.7) | [10] | 16.1 | (0.4) | [62] | 0.008 | 1 | 2.23 | 1.157 |
| **RBP4** | P02753 | 15.7 | (0.7) | [10] | 16.3 | (0.4) | [62] | 0.010 | 1 | 1.50 | 0.585 |
| **RELN** | J3KQ66 | 14.8 | (0.7) | [8] | 14.8 | (0.6) | [58] | 0.903 | 1 | 0.97 | -0.044 |
| **RGMB** | J3KNF6 | 14.7 | (0.7) | [10] | 14.9 | (0.6) | [62] | 0.512 | 1 | 1.17 | 0.227 |
| **RNASE1** | P07998 | 11.6 | (0.7) | [9] | 12.5 | (0.6) | [62] | 0.034 | 1 | 1.79 | 0.840 |
| **RNASET2** | A0A087WZM2 | 14.4 | (0.7) | [9] | 15.0 | (0.4) | [62] | 0.269 | 1 | 1.46 | 0.546 |
| **ROBO1** | Q9Y6N7 | 13.8 | (0.7) | [6] | 14.0 | (0.6) | [61] | 0.502 | 1 | 1.14 | 0.189 |
| **RTN4R** | H7C0V4 | 13.9 | (0.7) | [5] | 13.6 | (0.6) | [43] | 0.382 | 1 | 0.83 | -0.269 |
| **RTN4RL2** | Q86UN3 | 14.6 | (0.7) | [8] | 14.5 | (0.5) | [55] | 0.982 | 1 | 0.99 | -0.014 |
| **S100A1** | P23297 | 13.7 | (0.7) | [5] | 12.7 | (0.8) | [34] | 0.093 | 1 | 0.51 | -0.971 |
| **S100A9** | P06702 | 15.6 | (0.7) | [8] | 14.8 | (1.7) | [10] | 0.374 | 1 | 0.60 | -0.737 |
| **S100B** | P04271 | 13.8 | (0.7) | [7] | 15.3 | (1.2) | [61] | 0.002 | 1 | 2.82 | 1.496 |
| **SCG2** | P13521 | 14.3 | (0.7) | [10] | 14.6 | (0.5) | [62] | 0.186 | 1 | 1.29 | 0.367 |
| **SCG3** | Q8WXD2 | 15.9 | (0.7) | [10] | 16.1 | (0.4) | [62] | 0.261 | 1 | 1.19 | 0.251 |

| Name | Uniprot ID | Control | | | Communicating HC | | | P value | P adjusted | Fold change | $Log_2$(fold change) |
|---|---|---|---|---|---|---|---|---|---|---|---|
| | | Mean | (SD) | [N] | Mean | (SD) | [N] | | | | |
| **SCG5** | P05408-2 | 16.0 | (0.7) | [10] | 16.2 | (0.3) | [62] | 0.353 | 1 | 1.17 | 0.227 |
| **SCRG1** | O75711 | 16.8 | (0.7) | [9] | 17.2 | (0.7) | [62] | 0.24 | 1 | 1.29 | 0.367 |
| **SDF4** | Q9BRK5 | 13.1 | (0.7) | [8] | 13.6 | (0.9) | [52] | 0.126 | 1 | 1.34 | 0.422 |
| **SEC23IP** | Q9Y6Y8 | 14.3 | (0.7) | [8] | 14.1 | (0.6) | [51] | 0.574 | 1 | 0.89 | -0.168 |
| **SELENBP1** | Q13228 | 15.8 | (0.7) | [10] | 14.7 | (0.4) | [62] | 0.006 | 1 | 0.46 | -1.120 |
| **SELENOP** | A0A182DWH7 | 15.5 | (0.7) | [9] | 15.6 | (0.5) | [62] | 0.565 | 1 | 1.06 | 0.084 |
| **SELL** | P14151 | 15.4 | (0.7) | [10] | 15.1 | (0.4) | [62] | 0.167 | 1 | 0.78 | -0.358 |
| **SEMA3G** | Q9NS98 | 13.6 | (0.7) | [5] | 13.1 | (0.8) | [46] | 0.025 | 1 | 0.70 | -0.515 |
| **SEMA4B** | J3KNP4 | 13.4 | (0.7) | [5] | 13.8 | (0.4) | [60] | 0.093 | 1 | 1.33 | 0.411 |
| **SEMA7A** | O75326 | 14.5 | (0.7) | [10] | 14.8 | (0.7) | [62] | 0.42 | 1 | 1.20 | 0.263 |
| **SERPINA1** | P01009 | 18.4 | (0.7) | [10] | 18.0 | (0.3) | [62] | 0.014 | 1 | 0.79 | -0.340 |
| **SERPINA10** | G3V2W1 | 12.9 | (0.7) | [5] | 11.8 | (1.0) | [25] | 0.159 | 1 | 0.45 | -1.152 |
| **SERPINA3** | P01011 | 17.0 | (0.7) | [10] | 17.1 | (0.3) | [62] | 0.82 | 1 | 1.03 | 0.043 |
| **SERPINA4** | P29622 | 15.0 | (0.7) | [10] | 14.9 | (0.3) | [62] | 0.471 | 1 | 0.92 | -0.120 |
| **SERPINA5** | P05154 | 14.1 | (0.7) | [10] | 13.5 | (0.8) | [60] | 0.021 | 1 | 0.66 | -0.599 |
| **SERPINA6** | P08185 | 15.3 | (0.7) | [10] | 15.0 | (0.3) | [62] | 0.006 | 1 | 0.82 | -0.286 |
| **SERPINA7** | P05543 | 14.7 | (0.7) | [10] | 14.8 | (0.4) | [62] | 0.269 | 1 | 1.10 | 0.138 |
| **SERPINC1** | P01008 | 16.7 | (0.7) | [10] | 16.6 | (0.3) | [62] | 0.535 | 1 | 0.95 | -0.074 |
| **SERPIND1** | P05546 | 15.5 | (0.7) | [10] | 15.3 | (0.3) | [62] | 0.107 | 1 | 0.89 | -0.168 |
| **SERPINF1** | P36955 | 17.4 | (0.7) | [10] | 17.8 | (0.4) | [62] | 0.347 | 1 | 1.31 | 0.390 |
| **SERPINF2** | P08697 | 16.3 | (0.7) | [10] | 15.9 | (0.3) | [62] | 0.012 | 1 | 0.75 | -0.415 |

| Name | Uniprot ID | Control Mean | Control (SD) | Control [N] | Communicating HC Mean | Communicating HC (SD) | Communicating HC [N] | P value | P adjusted | Fold change | $Log_2$(fold change) |
|---|---|---|---|---|---|---|---|---|---|---|---|
| **SERPING1** | P05155 | 16.5 | (0.7) | [10] | 16.3 | (0.2) | [62] | 0.032 | 1 | 0.87 | -0.201 |
| **SERPINI1** | Q99574 | 14.7 | (0.7) | [9] | 14.9 | (0.6) | [62] | 0.679 | 1 | 1.12 | 0.163 |
| **SEZ6** | Q53EL9 | 13.9 | (0.7) | [8] | 13.9 | (0.6) | [59] | 0.867 | 1 | 0.98 | -0.029 |
| **SEZ6L** | B0QYH4 | 14.5 | (0.7) | [8] | 14.3 | (0.4) | [62] | 0.29 | 1 | 0.88 | -0.184 |
| **SEZ6L2** | A0A087WYL5 | 15.3 | (0.7) | [8] | 15.4 | (0.4) | [62] | 0.379 | 1 | 1.07 | 0.098 |
| **SHBG** | I3L145 | 14.7 | (0.7) | [7] | 13.5 | (0.4) | [61] | 0.02 | 1 | 0.46 | -1.120 |
| **SHISA6** | Q6ZSJ9 | 14.5 | (0.7) | [5] | 14.1 | (0.5) | [58] | 0.161 | 1 | 0.79 | -0.340 |
| **SIAE** | Q9HAT2 | 15.1 | (0.7) | [6] | 14.8 | (0.7) | [60] | 0.619 | 1 | 0.80 | -0.322 |
| **SIRPA** | P78324 | 14.8 | (0.7) | [10] | 15.0 | (0.5) | [62] | 0.526 | 1 | 1.15 | 0.202 |
| **SKP1** | E5RJR5 | 14.1 | (0.7) | [9] | 13.9 | (0.8) | [61] | 0.626 | 1 | 0.92 | -0.120 |
| **SLC3A2** | F5GZS6 | 13.1 | (0.7) | [5] | 13.0 | (0.6) | [58] | 0.526 | 1 | 0.91 | -0.136 |
| **SLITRK1** | Q96PX8 | 14.6 | (0.7) | [7] | 13.2 | (0.6) | [49] | 0.031 | 1 | 0.38 | -1.396 |
| **SLITRK4** | Q8IW52 | 13.1 | (0.7) | [8] | 13.5 | (0.5) | [61] | 0.329 | 1 | 1.27 | 0.345 |
| **SOD1** | P00441 | 16.1 | (0.7) | [10] | 16.2 | (0.3) | [62] | 0.67 | 1 | 1.04 | 0.057 |
| **SOD2** | P04179 | 13.6 | (0.7) | [10] | 13.7 | (0.4) | [61] | 0.394 | 1 | 1.10 | 0.138 |
| **SOD3** | P08294 | 15.3 | (0.7) | [10] | 16.1 | (0.6) | [62] | 0.085 | 1 | 1.80 | 0.848 |
| **SORCS3** | Q9UPU3 | 13.5 | (0.7) | [9] | 13.4 | (0.8) | [60] | 0.873 | 1 | 0.96 | -0.059 |
| **SORT1** | Q99523 | 14.0 | (0.7) | [6] | 14.2 | (0.4) | [61] | 0.707 | 1 | 1.09 | 0.124 |
| **SPARC** | P09486 | 15.1 | (0.7) | [10] | 16.6 | (0.8) | [62] | 0.028 | 1 | 2.83 | 1.501 |
| **SPARCL1** | Q14515 | 14.8 | (0.7) | [10] | 15.3 | (0.3) | [62] | 0.021 | 1 | 1.41 | 0.496 |
| **SPINT2** | K7EM91 | 13.3 | (0.7) | [5] | 12.9 | (0.5) | [50] | 0.161 | 1 | 0.73 | -0.454 |

| Name | Uniprot ID | Control Mean | Control (SD) | Control [N] | Communicating HC Mean | Communicating HC (SD) | Communicating HC [N] | P value | P adjusted | Fold change | $Log_2$(fold change) |
|---|---|---|---|---|---|---|---|---|---|---|---|
| **SPOCK1** | Q08629 | 13.9 | (0.7) | [8] | 14.3 | (0.5) | [62] | 0.271 | 1 | 1.32 | 0.401 |
| **SPOCK2** | Q92563 | 13.9 | (0.7) | [5] | 14.0 | (0.7) | [50] | 0.744 | 1 | 1.04 | 0.057 |
| **SPOCK3** | Q9BQ16 | 14.9 | (0.7) | [8] | 15.2 | (0.6) | [61] | 0.488 | 1 | 1.21 | 0.275 |
| **SPON1** | Q9HCB6 | 14.5 | (0.7) | [8] | 14.4 | (0.6) | [62] | 0.590 | 1 | 0.93 | -0.105 |
| **SPP1** | P10451 | 15.6 | (0.7) | [10] | 16.2 | (0.3) | [62] | 0.014 | 1 | 1.45 | 0.536 |
| **STMN1** | A2A2D0 | 12.7 | (0.7) | [5] | 12.6 | (3.9) | [12] | 0.945 | 1 | 0.90 | -0.152 |
| **SULF2** | Q8IWU5 | 14.3 | (0.7) | [10] | 15.0 | (0.4) | [62] | 0.019 | 1 | 1.55 | 0.632 |
| **SUSD5** | O60279 | 12.6 | (0.7) | [7] | 12.9 | (0.4) | [62] | 0.269 | 1 | 1.29 | 0.367 |
| **SYNE3** | G3V533 | 15.0 | (0.7) | [7] | 14.5 | (0.7) | [47] | 0.107 | 1 | 0.70 | -0.515 |
| **SYT2** | Q8N9I0 | 17.3 | (0.7) | [9] | 17.5 | (0.8) | [56] | 0.641 | 1 | 1.13 | 0.176 |
| **SAA1** | P0DJI8 | 14.4 | (0.7) | [7] | 14.4 | (1.2) | [18] | 0.894 | 1 | 1.03 | 0.043 |
| **SAA4** | P35542 | 15.6 | (0.7) | [10] | 14.7 | (0.6) | [62] | 0.014 | 1 | 0.55 | -0.862 |
| **TAGLN** | Q01995 | 14.7 | (0.7) | [8] | 14.1 | (0.6) | [62] | 0.043 | 1 | 0.64 | -0.644 |
| **TALDO1** | P37837 | 15.1 | (0.7) | [9] | 13.6 | (1.1) | [57] | 0.017 | 1 | 0.36 | -1.474 |
| **TCN2** | B5MBX2 | 15.6 | (0.7) | [8] | 15.5 | (1.2) | [60] | 0.778 | 1 | 0.93 | -0.105 |
| **TF** | P02787 | 17.7 | (0.7) | [10] | 17.9 | (0.2) | [62] | 0.154 | 1 | 1.17 | 0.227 |
| **TGFBI** | Q15582 | 14.5 | (0.7) | [10] | 14.5 | (0.3) | [62] | 0.952 | 1 | 0.99 | -0.014 |
| **TGOLN2** | F8W8W7 | 12.2 | (0.7) | [10] | 12.6 | (1.2) | [61] | 0.183 | 1 | 1.39 | 0.475 |
| **THBS1** | P07996 | 14.5 | (0.7) | [6] | 14.1 | (1.0) | [21] | 0.273 | 1 | 0.76 | -0.396 |
| **THBS2** | P35442 | 14.0 | (0.7) | [6] | 13.8 | (0.5) | [43] | 0.32 | 1 | 0.88 | -0.184 |
| **THY1** | E9PIM6 | 16.9 | (0.7) | [10] | 17.7 | (0.6) | [62] | 0.023 | 1 | 1.73 | 0.791 |

| Name | Uniprot ID | Control Mean | Control (SD) | Control [N] | Communicating HC Mean | Communicating HC (SD) | Communicating HC [N] | P value | P adjusted | Fold change | $Log_2$(fold change) |
|---|---|---|---|---|---|---|---|---|---|---|---|
| **TIMP1** | P01033 | 16.3 | (0.7) | [10] | 16.7 | (0.6) | [58] | 0.496 | 1 | 1.32 | 0.401 |
| **TIMP2** | P16035 | 15.4 | (0.7) | [8] | 15.9 | (0.3) | [60] | 0.162 | 1 | 1.42 | 0.506 |
| **TKT** | P29401 | 14.7 | (0.7) | [7] | 13.8 | (0.8) | [37] | 0.005 | 1 | 0.54 | -0.889 |
| **TMEM132A** | Q24JP5 | 14.7 | (0.7) | [6] | 14.1 | (0.5) | [62] | 0.011 | 1 | 0.67 | -0.578 |
| **TNFRSF21** | O75509 | 14.9 | (0.7) | [5] | 14.7 | (0.4) | [55] | 0.579 | 1 | 0.88 | -0.184 |
| **TNR** | Q92752 | 12.8 | (0.7) | [5] | 13.3 | (0.5) | [61] | 0.363 | 1 | 1.47 | 0.556 |
| **TNXB** | A0A140TA41 | 13.5 | (0.7) | [7] | 13.2 | (0.6) | [62] | 0.258 | 1 | 0.83 | -0.269 |
| **TPI1** | P60174 | 15.2 | (0.7) | [10] | 14.2 | (0.5) | [62] | 0.011 | 1 | 0.48 | -1.059 |
| **TPP1** | O14773 | 15.0 | (0.7) | [8] | 14.9 | (0.7) | [62] | 0.734 | 1 | 0.92 | -0.120 |
| **TPP2** | P29144 | 14.0 | (0.7) | [10] | 14.7 | (0.9) | [62] | 0.052 | 1 | 1.61 | 0.687 |
| **TREM2** | Q9NZC2 | 13.5 | (0.7) | [8] | 13.7 | (0.9) | [60] | 0.546 | 1 | 1.11 | 0.151 |
| **TTR** | P02766 | 18.6 | (0.7) | [10] | 19.9 | (0.8) | [62] | 0.018 | 1 | 2.45 | 1.293 |
| **TUBA1B** | P68363 | 16.0 | (0.7) | [9] | 16.5 | (1.1) | [61] | 0.406 | 1 | 1.35 | 0.433 |
| **TUBB** | P07437 | 16.6 | (0.7) | [7] | 15.8 | (1.0) | [58] | 0.105 | 1 | 0.59 | -0.761 |
| **TUBB4B** | P68371 | 15.9 | (0.7) | [9] | 15.8 | (1.0) | [61] | 0.878 | 1 | 0.96 | -0.059 |
| **TXN** | P10599 | 17.0 | (0.7) | [10] | 16.2 | (0.7) | [60] | 0.019 | 1 | 0.57 | -0.811 |
| **TXNDC17** | Q9BRA2 | 14.1 | (0.7) | [6] | 13.7 | (0.3) | [47] | 0.06 | 1 | 0.74 | -0.434 |
| **UBC** | F5H265 | 15.9 | (0.7) | [10] | 16.2 | (0.3) | [62] | 0.266 | 1 | 1.22 | 0.287 |
| **UBE2N** | P61088 | 14.1 | (0.7) | [7] | 13.1 | (0.7) | [13] | 0.066 | 1 | 0.48 | -1.059 |
| **VASN** | Q6EMK4 | 14.7 | (0.7) | [10] | 14.7 | (0.2) | [62] | 0.926 | 1 | 0.99 | -0.014 |
| **VCAM1** | P19320 | 13.3 | (0.7) | [8] | 13.7 | (0.4) | [60] | 0.092 | 1 | 1.38 | 0.465 |

| Name | Uniprot ID | Control | | | Communicating HC | | | P value | P adjusted | Fold change | $Log_2$(fold change) |
|---|---|---|---|---|---|---|---|---|---|---|---|
| | | Mean | (SD) | [N] | Mean | (SD) | [N] | | | | |
| **VCAN** | P13611 | 14.3 | (0.7) | [8] | 14.0 | (0.4) | [62] | 0.058 | 1 | 0.83 | -0.269 |
| **VGF** | O15240 | 15.4 | (0.7) | [10] | 15.3 | (0.4) | [62] | 0.637 | 1 | 0.88 | -0.184 |
| **VIP** | P01282 | 18.0 | (0.7) | [10] | 17.8 | (0.4) | [62] | 0.064 | 1 | 0.84 | -0.252 |
| **VSIG4** | Q9Y279 | 13.8 | (0.7) | [7] | 13.2 | (0.6) | [62] | 0.25 | 1 | 0.65 | -0.621 |
| **VSTM2A** | B5MCX6 | 14.0 | (0.7) | [9] | 14.7 | (0.5) | [62] | 0.018 | 1 | 1.59 | 0.669 |
| **VSTM2B** | A6NLU5 | 12.7 | (0.7) | [5] | 14.1 | (1.1) | [62] | 0.004 | 1 | 2.62 | 1.390 |
| **VTN** | P04004 | 17.3 | (0.7) | [10] | 16.9 | (0.4) | [62] | 0.159 | 1 | 0.76 | -0.396 |
| **VWF** | P04275 | 13.9 | (0.7) | [8] | 14.0 | (1.1) | [21] | 0.786 | 1 | 1.07 | 0.098 |
| **WDR1** | O75083 | 14.9 | (0.7) | [5] | 13.2 | (0.8) | [13] | 0.008 | 1 | 0.30 | -1.737 |
| **WFIKKN2** | C9J6G4 | 15.2 | (0.7) | [9] | 15.4 | (0.7) | [62] | 0.689 | 1 | 1.18 | 0.239 |
| **YWHAE** | P62258 | 14.7 | (0.7) | [9] | 13.6 | (0.8) | [61] | 0.009 | 1 | 0.47 | -1.089 |
| **YWHAG** | P61981 | 12.4 | (0.7) | [8] | 13.3 | (1.5) | [33] | 0.016 | 1 | 1.88 | 0.911 |
| **YWHAQ** | P27348 | 13.4 | (0.7) | [8] | 13.7 | (1.0) | [48] | 0.507 | 1 | 1.22 | 0.287 |
| **YWHAZ** | P63104 | 14.9 | (0.7) | [10] | 14.0 | (0.6) | [62] | 0.006 | 1 | 0.55 | -0.862 |
| **ZNF511-PRAP1** | H7BY64 | 14.6 | (0.7) | [5] | 14.1 | (0.6) | [59] | 0.397 | 1 | 0.71 | -0.494 |
