# Supplementary material for: Ventricular CSF proteomic profiles and predictors of surgical treatment outcome in chronic hydrocephalus
Source: Acta Neurochir (Wien). 2023 Oct 19;165(12):4059–70. doi: 10.1007/s00701-023-05832-y (PMC10739511; doi:10.1007/s00701-023-05832-y)
Supplement: Supplementary file 3 — Supplementary file3 (PDF 945 KB) [file 701_2023_5832_MOESM3_ESM.pdf]

Supplemental file 3

Control subjects compared to obstructive HC

All proteins identified by mass-spectrometry-based proteomics in at least 5 samples from control subjects and 10 samples from patients with obstructive hydrocephalus (HC). The protein that differed significantly between the two groups is highlighted in green.

		Control			Obstructive HC						
**Name**	**Uniprot ID**	**Mean**	**(SD)**	**[N]**	**Mean**	**(SD)**	**[N]**	**P value**	**P adjusted**	**Fold change**	**Log$_2$(fold change)**
**VIM**	P08670	18.2	(1.8)	[8]	13.5	(1.5)	[17]	<0.001	0.029	0.04	-4.64
**CLSTN3**	Q9BQT9	11.3	(0.8)	[10]	13.1	(1.9)	[28]	<0.001	0.185	3.41	1.77
**APOA2**	P02652	18.0	(0.7)	[10]	17.1	(0.7)	[28]	0.002	0.986	0.52	-0.94
**A1BG**	P04217-2	17.1	(0.5)	[10]	16.8	(0.3)	[28]	0.152	1	0.83	-0.27
**A2M**	P01023	17.2	(0.7)	[10]	16.9	(0.4)	[28]	0.204	1	0.81	-0.30
**ABHD14B**	Q96IU4	14.1	(1.2)	[8]	12.4	(0.8)	[12]	0.006	1	0.31	-1.69
**ACSBG2**	Q5FVE4	20.3	(1.3)	[10]	21.8	(1.2)	[28]	0.007	1	2.74	1.45
**ACTA2**	P62736	16.5	(1.0)	[10]	15.8	(0.9)	[28]	0.053	1	0.60	-0.74
**ACTB**	P60709	17.1	(1.1)	[10]	15.8	(0.9)	[28]	0.004	1	0.40	-1.32
**ACTBL2**	Q562R1	18.9	(1.1)	[10]	17.5	(1.0)	[28]	0.004	1	0.39	-1.36
**ACYP2**	P14621	12.5	(0.7)	[7]	12.5	(0.7)	[27]	0.952	1	0.99	-0.01
**ADAM22**	F8WAD8	12.0	(1.2)	[9]	12.3	(2.0)	[21]	0.689	1	1.18	0.24
**ADAMTS1**	Q9UHI8	14.7	(0.2)	[5]	14.4	(0.3)	[15]	0.043	1	0.83	-0.27
**ADGRB2**	A2A3C1	15.4	(1.0)	[9]	15.2	(1.0)	[26]	0.592	1	0.86	-0.22
**ADGRB3**	O60242	12.2	(1.0)	[5]	11.6	(0.9)	[17]	0.287	1	0.66	-0.60
**ADGRL1**	O94910	14.1	(0.3)	[7]	14.2	(1.0)	[26]	0.703	1	1.06	0.08

Name	Uniprot ID	Control Mean	Control (SD)	Control [N]	Obstructive HC Mean	Obstructive HC (SD)	Obstructive HC [N]	P value	P adjusted	Fold change	Log_2(fold change)
**ADGRL3**	E7EN28	13.8	(0.6)	[6]	13.8	(0.8)	[24]	0.915	1	1.02	0.03
**ADIPOQ**	Q15848	13.0	(1.1)	[8]	12.0	(0.8)	[19]	0.048	1	0.51	-0.97
**AEBP1**	Q8IUX7	13.6	(0.5)	[7]	14.0	(0.6)	[26]	0.105	1	1.30	0.38
**AFM**	P43652	15.2	(0.3)	[10]	15.0	(0.4)	[28]	0.16	1	0.88	-0.18
**AGA**	P20933	13.9	(1.0)	[6]	13.9	(2.1)	[27]	0.997	1	1.00	0.00
**AGRN**	O00468-6	13.9	(0.9)	[9]	14.5	(0.9)	[28]	0.122	1	1.48	0.57
**AGT**	P01019	16.9	(0.7)	[10]	17.1	(0.5)	[28]	0.315	1	1.19	0.25
**AHSG**	P02765	17.0	(0.6)	[10]	16.8	(0.4)	[28]	0.221	1	0.83	-0.27
**AK1**	P00568	15.2	(1.5)	[6]	14.4	(0.9)	[13]	0.249	1	0.56	-0.84
**ALB**	P02768	19.1	(0.3)	[10]	19.2	(0.3)	[28]	0.253	1	1.09	0.12
**ALCAM**	Q13740	14.7	(1.1)	[10]	15.4	(0.6)	[28]	0.064	1	1.66	0.73
**ALDOA**	P04075	15.7	(1.1)	[10]	14.8	(0.7)	[28]	0.033	1	0.55	-0.86
**ALDOC**	P09972	14.4	(0.7)	[10]	14.6	(0.7)	[28]	0.487	1	1.14	0.19
**AMBP**	P02760	16.1	(0.7)	[10]	15.7	(0.5)	[28]	0.084	1	0.74	-0.43
**ANXA5**	P08758	15.7	(1.3)	[9]	14.6	(0.7)	[13]	0.028	1	0.44	-1.18
**APCS**	P02743	16.0	(1.9)	[10]	14.7	(1.0)	[25]	0.071	1	0.41	-1.29
**APLP1**	B7Z4G8	16.4	(0.7)	[10]	16.9	(0.5)	[28]	0.068	1	1.42	0.51
**APLP2**	Q06481	14.9	(0.8)	[10]	14.9	(0.9)	[28]	0.866	1	1.04	0.06
**APOA1**	P02647	18.8	(1.1)	[10]	18.0	(0.8)	[28]	0.062	1	0.58	-0.79
**APOA4**	P06727	16.7	(0.8)	[10]	16.3	(0.5)	[28]	0.146	1	0.75	-0.42
**APOB**	P04114	16.4	(1.9)	[10]	14.6	(1.4)	[24]	0.019	1	0.29	-1.79

Name	Uniprot ID	Control Mean	(SD)	[N]	Obstructive HC Mean	(SD)	[N]	P value	P adjusted	Fold change	Log_2(fold change)
**APOC1**	K7ERI9	16.5	(1.3)	[10]	15.6	(0.8)	[27]	0.047	1	0.51	-0.97
**APOC3**	B0YIW2	18.1	(2.1)	[10]	15.9	(1.4)	[28]	0.011	1	0.23	-2.12
**APOC4-APOC2**	A0A024R0T9	15.9	(1.6)	[10]	14.7	(1.4)	[18]	0.06	1	0.43	-1.22
**APOD**	C9JF17	16.5	(0.7)	[10]	16.4	(0.8)	[28]	0.757	1	0.94	-0.09
**APOE**	P02649	17.6	(0.7)	[10]	17.2	(0.8)	[28]	0.118	1	0.74	-0.43
**APOH**	P02749	15.8	(0.6)	[10]	15.8	(0.5)	[28]	0.843	1	0.97	-0.04
**APOL1**	O14791	14.9	(1.9)	[9]	13.4	(1.1)	[18]	0.051	1	0.35	-1.51
**APOM**	O95445	16.5	(1.3)	[9]	15.6	(0.8)	[25]	0.073	1	0.53	-0.92
**APP**	P05067	15.2	(0.8)	[10]	15.4	(0.5)	[28]	0.292	1	1.22	0.29
**ARPC4-TTLL3**	A0A0A6YYG9	16.3	(0.3)	[10]	16.4	(0.3)	[28]	0.376	1	1.08	0.11
**ART3**	E7ESB3	14.1	(1.2)	[10]	15.2	(0.7)	[28]	0.014	1	2.20	1.14
**ASAH1**	A0A1B0GTM3	14.8	(1.1)	[5]	15.2	(0.7)	[24]	0.507	1	1.30	0.38
**ATP1A1**	P05023	14.2	(0.7)	[5]	13.9	(0.5)	[14]	0.38	1	0.81	-0.30
**ATP6AP1**	Q15904	15.1	(0.8)	[10]	14.7	(0.7)	[28]	0.23	1	0.78	-0.36
**ATRN**	O75882	14.6	(0.4)	[9]	14.1	(0.5)	[26]	0.005	1	0.71	-0.49
**AXL**	P30530	14.7	(1.2)	[7]	15.6	(0.5)	[24]	0.091	1	1.90	0.93
**AZGP1**	P25311	16.4	(0.4)	[10]	16.5	(0.4)	[28]	0.4	1	1.09	0.12
**B2M**	P61769	16.4	(0.9)	[10]	17.2	(0.6)	[28]	0.031	1	1.65	0.72
**B3GALNT1**	O75752	13.2	(0.4)	[7]	13.5	(0.6)	[17]	0.124	1	1.26	0.33
**B4GAT1**	O43505	16.5	(1.0)	[10]	16.9	(0.9)	[28]	0.401	1	1.24	0.31
**BASP1**	P80723	11.4	(0.9)	[10]	12.3	(0.9)	[28]	0.017	1	1.82	0.86

Name	Uniprot ID	Control Mean	(SD)	[N]	Obstructive HC Mean	(SD)	[N]	P value	P adjusted	Fold change	Log_2(fold change)
**BCAN**	Q96GW7	15.3	(0.9)	[10]	16.3	(0.8)	[28]	0.005	1	2.06	1.04
**BGN**	P21810	14.9	(0.9)	[8]	13.6	(1.2)	[27]	0.006	1	0.41	-1.29
**BLVRB**	P30043	15.8	(2.1)	[9]	14.0	(1.4)	[17]	0.037	1	0.29	-1.79
**BTD**	P43251	15.8	(0.7)	[10]	16.5	(0.5)	[28]	0.019	1	1.58	0.66
**C16orf89**	A0A0A0MT71	14.4	(0.9)	[9]	14.4	(0.7)	[24]	0.911	1	1.03	0.04
**C1QA**	P02745	14.8	(0.5)	[9]	14.4	(0.7)	[28]	0.099	1	0.78	-0.36
**C1QB**	D6R934	15.4	(0.7)	[10]	15.4	(0.4)	[28]	0.974	1	0.99	-0.01
**C1QC**	P02747	17.1	(0.6)	[10]	16.9	(0.3)	[28]	0.33	1	0.86	-0.22
**C1QTNF5**	Q9BXJ0	13.6	(1.4)	[5]	13.6	(1.0)	[25]	0.949	1	1.03	0.04
**C1R**	B4DPQ0	15.6	(0.5)	[10]	15.4	(0.4)	[28]	0.185	1	0.85	-0.23
**C1RL**	Q9NZP8	14.3	(0.7)	[9]	13.8	(0.5)	[21]	0.081	1	0.71	-0.49
**C1S**	P09871	16.3	(0.4)	[10]	15.9	(0.6)	[28]	0.075	1	0.8	-0.32
**C2**	P06681	15.0	(0.5)	[10]	14.6	(0.3)	[28]	0.031	1	0.76	-0.40
**C2orf40**	B8ZZE5	15.2	(2.0)	[9]	16.7	(1.7)	[28]	0.06	1	2.86	1.52
**C3**	P01024	17.3	(0.4)	[10]	17.1	(0.2)	[28]	0.146	1	0.87	-0.20
**C4A**	A0A0G2JPR0	15.6	(0.9)	[10]	15.6	(0.7)	[28]	0.972	1	0.99	-0.01
**C4B**	P0C0L5	16.8	(0.4)	[10]	16.8	(0.4)	[28]	0.992	1	1.00	0.00
**C4BPA**	P04003	14.2	(1.8)	[9]	12.8	(1.4)	[15]	0.064	1	0.37	-1.43
**C5**	P01031	14.7	(0.7)	[10]	14.0	(0.5)	[28]	0.01	1	0.60	-0.74
**C6**	P13671	15.5	(0.6)	[10]	15.0	(0.4)	[28]	0.043	1	0.71	-0.49
**C7**	P10643	15.8	(0.6)	[10]	15.6	(0.7)	[28]	0.537	1	0.91	-0.14

Name	Uniprot ID	Control Mean	Control (SD)	Control [N]	Obstructive HC Mean	Obstructive HC (SD)	Obstructive HC [N]	P value	P adjusted	Fold change	Log_2(fold change)
**C8A**	P07357	15.3	(0.5)	[10]	15.4	(0.6)	[28]	0.569	1	1.09	0.12
**C8B**	F5GY80	14.1	(0.4)	[10]	14.1	(0.5)	[28]	0.925	1	0.99	-0.01
**C8G**	P07360	14.5	(0.6)	[6]	14.5	(0.7)	[27]	0.938	1	0.98	-0.03
**C9**	P02748	15.5	(0.4)	[10]	15.1	(0.4)	[28]	0.024	1	0.79	-0.34
**CA1**	P00915	17.5	(2.1)	[10]	15.6	(1.7)	[25]	0.018	1	0.26	-1.94
**CA2**	P00918	15.4	(1.7)	[9]	14.2	(1.1)	[22]	0.074	1	0.44	-1.18
**CACHD1**	A0A0A0MQY7	13.3	(0.6)	[8]	13.4	(0.6)	[27]	0.678	1	1.08	0.11
**CACNA2D1**	P54289	14.6	(0.7)	[10]	14.6	(0.6)	[28]	0.965	1	0.99	-0.01
**CADM1**	A0A087X0T8	15.7	(1.1)	[8]	15.7	(1.0)	[28]	0.96	1	0.99	-0.01
**CADM2**	Q8N3J6	14.6	(0.4)	[6]	14.6	(0.5)	[28]	0.991	1	1.00	0.00
**CADM3**	Q8N126	15.3	(0.6)	[10]	15.4	(0.5)	[28]	0.735	1	1.05	0.07
**CADM4**	Q8NFZ8	15.9	(0.8)	[9]	16.5	(0.6)	[28]	0.053	1	1.57	0.65
**CALR**	P27797	13.5	(1.0)	[10]	14.2	(1.0)	[28]	0.059	1	1.69	0.76
**CAMK2A**	Q9UQM7	14.7	(0.6)	[5]	14.4	(0.9)	[14]	0.536	1	0.85	-0.23
**CANT1**	Q8WVQ1	13.8	(0.4)	[6]	13.6	(0.5)	[22]	0.307	1	0.87	-0.20
**CARTPT**	Q16568	13.7	(0.8)	[9]	13.8	(0.6)	[27]	0.659	1	1.09	0.12
**CASP14**	P31944	14.0	(0.5)	[7]	14.2	(1.1)	[15]	0.688	1	1.10	0.14
**CAT**	P04040	15.5	(1.1)	[7]	14.0	(1.0)	[14]	0.01	1	0.34	-1.56
**CBLN1**	P23435	13.4	(1.4)	[5]	12.9	(1.3)	[11]	0.505	1	0.71	-0.49
**CBR1**	P16152	14.7	(0.9)	[8]	14.2	(0.8)	[28]	0.242	1	0.73	-0.45
**CCDC93**	F8W9X7	14.1	(0.2)	[5]	14.5	(0.6)	[20]	0.036	1	1.30	0.38

Name	Uniprot ID	Control Mean	(SD)	[N]	Obstructive HC Mean	(SD)	[N]	P value	P adjusted	Fold change	Log_2(fold change)
**CCP110**	O43303	21.1	(0.7)	[7]	21.5	(0.8)	[19]	0.252	1	1.31	0.39
**CD14**	P08571	16.0	(1.0)	[10]	16.2	(0.5)	[28]	0.626	1	1.12	0.16
**CD44**	H0YD13	16.5	(0.8)	[10]	17.3	(0.8)	[28]	0.011	1	1.83	0.87
**CD59**	E9PNW4	15.1	(1.1)	[8]	15.8	(0.9)	[28]	0.132	1	1.61	0.69
**CD9**	A6NNI4	15.5	(1.5)	[5]	15.9	(1.0)	[16]	0.603	1	1.31	0.39
**CD99**	P14209	15.4	(0.9)	[8]	16.2	(0.9)	[28]	0.059	1	1.68	0.75
**CD99L2**	Q8TCZ2	16.2	(0.6)	[10]	16.9	(0.5)	[28]	0.003	1	1.66	0.73
**CDH13**	P55290	15.3	(0.8)	[10]	15.5	(0.6)	[28]	0.574	1	1.11	0.15
**CDH2**	P19022	15.7	(0.8)	[10]	16.4	(0.5)	[28]	0.048	1	1.53	0.61
**CDH4**	P55283	13.7	(0.8)	[6]	13.8	(1.0)	[27]	0.856	1	1.05	0.07
**CDH6**	D6RF86	13.2	(0.9)	[6]	13.1	(0.9)	[19]	0.837	1	0.94	-0.09
**CETP**	P11597	15.6	(0.6)	[6]	15.9	(0.8)	[13]	0.298	1	1.29	0.37
**CFD**	K7ERG9	15.7	(0.6)	[10]	16.1	(0.5)	[28]	0.075	1	1.34	0.42
**CFH**	P08603	16.0	(0.5)	[10]	15.4	(0.3)	[28]	0.008	1	0.69	-0.54
**CFHR1**	B1AKG0	15.4	(0.8)	[10]	15.4	(0.9)	[28]	0.84	1	0.96	-0.06
**CFI**	E7ETH0	14.7	(0.2)	[10]	14.7	(0.3)	[28]	0.603	1	0.97	-0.04
**CFL1**	E9PK25	14.8	(1.2)	[10]	13.9	(0.9)	[24]	0.07	1	0.57	-0.81
**CGREF1**	Q99674	13.8	(1.1)	[10]	13.8	(1.0)	[27]	0.871	1	0.96	-0.06
**CHGA**	P10645	16.1	(0.7)	[10]	16.6	(0.6)	[28]	0.046	1	1.44	0.53
**CHGB**	P05060	16.0	(0.6)	[10]	16.1	(0.6)	[28]	0.703	1	1.06	0.08
**CHI3L1**	P36222	15.4	(1.4)	[10]	16.4	(0.7)	[28]	0.054	1	1.96	0.97

Name	Uniprot ID	Control Mean	(SD)	[N]	Obstructive HC Mean	(SD)	[N]	P value	P adjusted	Fold change	Log_2(fold change)
**CHI3L2**	Q15782	12.6	(1.9)	[5]	13.4	(1.0)	[25]	0.397	1	1.75	0.81
**CHL1**	O00533	15.4	(0.8)	[10]	15.6	(0.6)	[28]	0.378	1	1.20	0.26
**CHST10**	O43529	17.7	(1.8)	[5]	18.9	(2.3)	[19]	0.251	1	2.30	1.20
**CKB**	P12277	15.4	(1.3)	[9]	15.4	(1.4)	[26]	0.995	1	1.00	0.00
**CKM**	P06732	17.7	(2.6)	[6]	15.6	(2.0)	[11]	0.123	1	0.23	-2.12
**CLCNKB**	A0A087X136	16.2	(0.9)	[10]	16.4	(0.8)	[28]	0.564	1	1.14	0.19
**CLEC11A**	Q9Y240	13.0	(0.7)	[7]	13.1	(0.7)	[16]	0.697	1	1.09	0.12
**CLEC3B**	E9PHK0	16.4	(0.4)	[10]	16.9	(0.4)	[28]	0.008	1	1.39	0.48
**CLN5**	A0A024R644	14.4	(1.3)	[5]	14.4	(0.8)	[26]	0.905	1	1.05	0.07
**CLSTN1**	O94985	16.4	(0.7)	[10]	16.7	(0.5)	[28]	0.308	1	1.19	0.25
**CLU**	P10909	16.6	(0.5)	[10]	17.2	(0.4)	[28]	0.01	1	1.44	0.53
**CNDP1**	Q96KN2	16.7	(0.6)	[10]	17.3	(0.5)	[28]	0.017	1	1.50	0.58
**CNTFR**	P26992	13.7	(0.4)	[8]	13.0	(1.2)	[17]	0.039	1	0.60	-0.74
**CNTN1**	Q12860	15.5	(0.8)	[10]	16.0	(0.5)	[28]	0.082	1	1.42	0.51
**CNTN2**	A0A1W2PQ11	15.3	(1.2)	[10]	16.5	(0.9)	[28]	0.015	1	2.22	1.15
**CNTNAP4**	A0A087WTA1	14.3	(0.7)	[7]	14.1	(0.9)	[27]	0.528	1	0.86	-0.22
**COL18A1**	P39060	14.8	(0.7)	[9]	15.0	(0.7)	[28]	0.458	1	1.15	0.20
**COL1A1**	P02452	15.6	(1.5)	[9]	14.2	(0.8)	[25]	0.031	1	0.39	-1.36
**COL1A2**	A0A087WTA8	15.3	(1.8)	[10]	14.4	(0.9)	[28]	0.119	1	0.51	-0.97
**COL3A1**	P02461	13.9	(1.5)	[5]	13.4	(0.4)	[12]	0.501	1	0.70	-0.51
**COL6A1**	A0A087X0S5	14.8	(0.8)	[10]	15.2	(0.5)	[28]	0.134	1	1.34	0.42

Name	Uniprot ID	Control Mean	(SD)	[N]	Obstructive HC Mean	(SD)	[N]	P value	P adjusted	Fold change	Log_2(fold change)
**COL6A3**	P12111	13.9	(0.5)	[8]	13.6	(0.5)	[19]	0.233	1	0.82	-0.29
**COLEC12**	Q5KU26	14.2	(0.8)	[9]	14.6	(0.6)	[27]	0.142	1	1.38	0.46
**CP**	P00450	16.3	(0.4)	[10]	16.4	(0.4)	[28]	0.579	1	1.06	0.08
**CPB2**	A0A087WSY5	15.4	(0.4)	[10]	15.4	(0.4)	[28]	0.871	1	0.98	-0.03
**CPE**	P16870	16.5	(1.1)	[10]	17.3	(0.6)	[28]	0.056	1	1.74	0.80
**CPN2**	P22792	15.3	(1.2)	[10]	14.6	(0.8)	[21]	0.105	1	0.59	-0.76
**CPQ**	Q9Y646	15.3	(1.0)	[10]	15.9	(0.6)	[28]	0.116	1	1.51	0.59
**CPVL**	Q9H3G5	14.7	(1.1)	[8]	15.2	(0.8)	[27]	0.241	1	1.45	0.54
**CRP**	P02741	14.0	(1.0)	[5]	14.4	(1.0)	[20]	0.508	1	1.27	0.34
**CRTAC1**	A0A0C4DFP6	15.3	(0.6)	[10]	15.5	(0.6)	[28]	0.29	1	1.19	0.25
**CSF1**	P09603	14.5	(0.7)	[10]	14.9	(0.6)	[27]	0.116	1	1.34	0.42
**CSF1R**	E9PEK4	15.5	(0.6)	[8]	15.7	(0.6)	[28]	0.436	1	1.15	0.20
**CST3**	P01034	17.6	(0.6)	[10]	18.0	(0.5)	[28]	0.043	1	1.41	0.50
**CSTB**	P04080	15.3	(1.1)	[10]	14.4	(0.7)	[22]	0.038	1	0.55	-0.86
**CTBS**	Q01459	14.5	(0.7)	[8]	15.2	(0.7)	[27]	0.039	1	1.60	0.68
**CTSA**	P10619	13.6	(1.0)	[6]	13.6	(0.8)	[27]	0.965	1	1.01	0.01
**CTSB**	P07858	13.6	(1.0)	[9]	14.0	(0.7)	[27]	0.419	1	1.24	0.31
**CTSC**	P53634	14.1	(0.8)	[5]	13.8	(0.9)	[19]	0.458	1	0.79	-0.34
**CTSD**	A0A1B0GV23	16.1	(1.5)	[10]	17.2	(1.1)	[28]	0.054	1	2.14	1.10
**CTSF**	Q9UBX1	14.5	(0.9)	[6]	15.0	(0.3)	[26]	0.23	1	1.42	0.51
**CTSH**	A0A087X0D5	14.6	(1.2)	[9]	15.6	(1.0)	[28]	0.046	1	1.97	0.98

Name	Uniprot ID	Control Mean	(SD)	[N]	Obstructive HC Mean	(SD)	[N]	P value	P adjusted	Fold change	Log_2(fold change)
**CTSL**	P07711	15.3	(0.9)	[8]	15.7	(0.8)	[28]	0.285	1	1.33	0.41
**CTSS**	P25774	14.5	(0.4)	[8]	14.2	(0.4)	[23]	0.219	1	0.86	-0.22
**CTSZ**	Q9UBR2	15.5	(1.1)	[8]	15.9	(1.0)	[27]	0.384	1	1.31	0.39
**CUTA**	O60888	15.2	(0.9)	[9]	15.3	(1.2)	[28]	0.768	1	1.08	0.11
**CYCS**	C9JFR7	13.5	(0.9)	[9]	13.2	(0.7)	[20]	0.391	1	0.82	-0.29
**DAG1**	Q14118	15.3	(0.5)	[10]	15.7	(0.5)	[28]	0.052	1	1.33	0.41
**DBI**	A0A0A0MTI5	14.7	(0.6)	[10]	15.5	(0.6)	[28]	0.004	1	1.69	0.76
**DCN**	P07585	14.1	(0.6)	[7]	14.3	(0.8)	[22]	0.638	1	1.09	0.12
**DDAH1**	O94760	13.5	(0.5)	[7]	13.8	(1.3)	[18]	0.431	1	1.22	0.29
**DKK3**	F6SYF8	16.2	(0.7)	[10]	16.4	(0.6)	[28]	0.426	1	1.16	0.21
**DPP7**	Q9UHL4	13.8	(1.1)	[8]	14.3	(0.8)	[28]	0.237	1	1.42	0.51
**DPYSL2**	A0A1C7CYX9	14.9	(0.8)	[5]	14.6	(1.1)	[22]	0.547	1	0.83	-0.27
**DSC2**	Q02487	13.8	(0.8)	[7]	14.4	(0.6)	[24]	0.092	1	1.54	0.62
**ECM1**	Q16610	15.0	(0.5)	[10]	14.8	(0.5)	[28]	0.5	1	0.92	-0.12
**ECM2**	O94769	14.5	(0.5)	[8]	14.2	(0.8)	[25]	0.259	1	0.82	-0.29
**EEF1A1**	P68104	14.4	(1.2)	[9]	14.7	(1.5)	[18]	0.566	1	1.24	0.31
**EFCAB14**	O75071	14.4	(0.7)	[6]	14.2	(0.8)	[17]	0.656	1	0.90	-0.15
**EFEMP1**	A0A0U1RQV3	16.0	(1.3)	[10]	17.2	(0.7)	[28]	0.016	1	2.29	1.20
**EFNA1**	P20827	14.0	(1.2)	[7]	13.7	(1.3)	[24]	0.591	1	0.81	-0.30
**EIF5A**	I3L397	15.0	(1.1)	[8]	13.1	(1.0)	[11]	0.002	1	0.27	-1.89
**ENDOD1**	O94919	15.1	(1.0)	[10]	15.8	(0.7)	[28]	0.047	1	1.72	0.78

Name	Uniprot ID	Control Mean	(SD)	[N]	Obstructive HC Mean	(SD)	[N]	P value	P adjusted	Fold change	Log_2(fold change)
**ENO1**	P06733	15.8	(0.7)	[10]	15.4	(0.9)	[27]	0.181	1	0.76	-0.40
**ENO2**	P09104	14.7	(0.6)	[9]	14.6	(1.1)	[28]	0.72	1	0.93	-0.10
**ENPP2**	E7EUF1	16.4	(1.6)	[10]	17.8	(1.2)	[28]	0.024	1	2.68	1.42
**ENPP4**	Q9Y6X5	13.1	(1.4)	[8]	13.4	(0.9)	[26]	0.636	1	1.20	0.26
**EPDR1**	Q9UM22	14.1	(1.0)	[6]	14.0	(0.7)	[25]	0.95	1	0.98	-0.03
**EPHA4**	E9PG71	15.2	(0.7)	[9]	15.3	(0.8)	[28]	0.845	1	1.04	0.06
**ERN1**	O75460	16.8	(1.7)	[8]	15.5	(1.3)	[26]	0.081	1	0.41	-1.29
**ESD**	H7BZT7	13.9	(1.1)	[7]	13.4	(0.7)	[11]	0.331	1	0.72	-0.47
**EXTL2**	Q9UBQ6	14.5	(0.8)	[10]	14.8	(0.7)	[24]	0.476	1	1.16	0.21
**F10**	P00742	14.3	(0.6)	[9]	14.5	(0.5)	[17]	0.544	1	1.11	0.15
**F12**	P00748	16.3	(0.9)	[10]	15.8	(0.5)	[28]	0.091	1	0.69	-0.54
**F2**	P00734	16.3	(0.5)	[10]	16.0	(0.3)	[28]	0.106	1	0.82	-0.29
**F5**	A0A0A0MRJ7	14.9	(1.2)	[10]	15.8	(1.0)	[28]	0.066	1	1.83	0.87
**F9**	P00740	13.5	(0.5)	[10]	13.4	(0.7)	[28]	0.779	1	0.96	-0.06
**FAM3C**	Q92520	15.3	(0.9)	[10]	15.7	(0.6)	[28]	0.216	1	1.32	0.40
**FAT2**	Q9NYQ8	13.5	(0.6)	[6]	12.9	(0.9)	[16]	0.131	1	0.70	-0.51
**FBLN1**	P23142	16.2	(0.8)	[10]	16.8	(0.7)	[28]	0.061	1	1.47	0.56
**FBLN5**	G3V4U0	14.3	(0.7)	[8]	14.5	(0.9)	[27]	0.442	1	1.18	0.24
**FBLN7**	Q53RD9	12.7	(0.7)	[5]	12.7	(0.8)	[14]	0.87	1	0.95	-0.07
**FCGBP**	Q9Y6R7	14.6	(1.3)	[8]	14.7	(0.7)	[26]	0.923	1	1.03	0.04
**FCGR3A**	A0A1W2PQB1	14.3	(1.1)	[10]	14.4	(0.9)	[28]	0.724	1	1.10	0.14

Name	Uniprot ID	Control Mean	Control (SD)	Control [N]	Obstructive HC Mean	Obstructive HC (SD)	Obstructive HC [N]	P value	P adjusted	Fold change	Log_2(fold change)
**FETUB**	Q9UGM5	14.6	(0.8)	[9]	14.2	(0.8)	[25]	0.274	1	0.78	-0.36
**FGA**	P02671	15.3	(1.0)	[10]	14.3	(0.6)	[28]	0.012	1	0.50	-1.00
**FGB**	P02675	16.5	(1.2)	[10]	15.6	(0.7)	[28]	0.08	1	0.57	-0.81
**FGFR2**	A0A0A0MR25	16.2	(1.1)	[5]	15.8	(0.7)	[27]	0.518	1	0.78	-0.36
**FGG**	P02679	15.5	(1.3)	[10]	14.9	(0.7)	[28]	0.214	1	0.67	-0.58
**FKBP1A**	P62942	15	(0.9)	[9]	14.6	(0.3)	[14]	0.297	1	0.78	-0.36
**FMOD**	Q06828	13.9	(1.0)	[8]	13.8	(0.7)	[19]	0.718	1	0.90	-0.15
**FN1**	P02751	16.1	(0.4)	[10]	15.6	(0.6)	[28]	0.003	1	0.70	-0.51
**FRRS1L**	Q9P0K9	10.7	(1.1)	[6]	10.8	(1.8)	[12]	0.927	1	1.05	0.07
**FRZB**	Q92765	14.3	(0.7)	[8]	14.3	(1.0)	[26]	0.835	1	1.05	0.07
**FSTL1**	Q12841	14.0	(1.0)	[8]	14.0	(1.0)	[28]	0.985	1	0.99	-0.01
**FSTL4**	Q6MZW2	13.8	(0.9)	[7]	13.2	(0.8)	[15]	0.19	1	0.68	-0.56
**FTH1**	P02794	12.0	(0.7)	[7]	12.4	(1.2)	[18]	0.337	1	1.30	0.38
**FTL**	P02792	15.0	(1.5)	[5]	13.5	(0.7)	[24]	0.085	1	0.34	-1.56
**FUCA1**	P04066	14.1	(0.8)	[8]	14.6	(0.8)	[28]	0.16	1	1.39	0.48
**FUCA2**	Q9BTY2	14.1	(0.9)	[10]	15.0	(0.7)	[27]	0.018	1	1.84	0.88
**FXYD6**	Q9H0Q3	14.8	(1.5)	[5]	15.3	(1.0)	[27]	0.455	1	1.47	0.56
**GALNT2**	Q10471	13.8	(0.4)	[8]	14.0	(0.7)	[26]	0.414	1	1.13	0.18
**GANAB**	Q14697	13.3	(0.6)	[8]	12.5	(0.5)	[24]	0.015	1	0.60	-0.74
**GAPDH**	P04406	15.6	(1.1)	[10]	14.8	(1.1)	[28]	0.073	1	0.59	-0.76
**GC**	P02774	16.7	(0.2)	[10]	16.6	(0.4)	[28]	0.193	1	0.92	-0.12

Name	Uniprot ID	Control			Obstructive HC			P value	P adjusted	Fold change	Log$_2$(fold change)
		Mean	(SD)	[N]	Mean	(SD)	[N]				
**GDA**	Q9Y2T3	14.2	(0.5)	[6]	13.6	(0.9)	[27]	0.07	1	0.69	-0.54
**GDI1**	P31150	14.8	(0.4)	[7]	14.7	(0.8)	[19]	0.772	1	0.95	-0.07
**GDI2**	P50395	14.2	(0.6)	[9]	13.7	(1.1)	[28]	0.119	1	0.72	-0.47
**GGH**	Q92820	14.8	(0.8)	[10]	14.9	(0.6)	[28]	0.704	1	1.08	0.11
**GLOD4**	F6TLX2	15.0	(0.9)	[7]	15.5	(1.5)	[14]	0.379	1	1.38	0.46
**GM2A**	P17900	15.9	(0.9)	[10]	16.5	(0.8)	[28]	0.085	1	1.53	0.61
**GNPTG**	Q9UJJ9	15.6	(0.7)	[8]	15.8	(0.5)	[27]	0.639	1	1.10	0.14
**GOLM1**	Q8NBJ4	13.5	(0.4)	[8]	13.6	(0.7)	[27]	0.58	1	1.07	0.10
**GOT1**	P17174	15.3	(0.8)	[10]	15.0	(0.7)	[28]	0.244	1	0.79	-0.34
**GPC1**	P35052	13.6	(0.6)	[5]	13.3	(1.7)	[22]	0.631	1	0.86	-0.22
**GPI**	A0A0A0MTS2	13.6	(0.6)	[8]	13.1	(0.8)	[15]	0.098	1	0.70	-0.51
**GPLD1**	P80108	15.2	(1.2)	[7]	14.0	(0.7)	[14]	0.035	1	0.44	-1.18
**GPR37**	O15354	15.5	(0.8)	[8]	16.3	(0.6)	[28]	0.024	1	1.72	0.78
**GPR37L1**	O60883	14.9	(1.1)	[10]	14.7	(1.1)	[28]	0.606	1	0.86	-0.22
**GPX3**	A0A087X1J7	16.2	(0.9)	[10]	16.2	(1.0)	[28]	0.934	1	1.02	0.03
**GRIA4**	G3V164	12.5	(1.1)	[6]	12.9	(0.6)	[17]	0.44	1	1.31	0.39
**GSN**	P06396	16.4	(0.4)	[10]	16.7	(0.3)	[28]	0.178	1	1.16	0.21
**GSS**	P48637	15.6	(0.4)	[5]	13.8	(1.3)	[10]	0.002	1	0.29	-1.79
**GSTO1**	P78417	15.2	(0.8)	[7]	14.2	(0.8)	[28]	0.013	1	0.50	-1.00
**GSTP1**	P09211	16.1	(0.9)	[10]	15.3	(0.8)	[28]	0.045	1	0.60	-0.74
**HARS**	B3KWE1	20.9	(0.9)	[5]	21.3	(1.1)	[17]	0.414	1	1.33	0.41

Name	Uniprot ID	Control Mean	Control (SD)	Control [N]	Obstructive HC Mean	Obstructive HC (SD)	Obstructive HC [N]	P value	P adjusted	Fold change	Log_2(fold change)
**HBA1**	P69905	21.1	(2.1)	[10]	19.2	(2.2)	[28]	0.021	1	0.25	-2.00
**HBB**	P68871	21.2	(2.1)	[10]	19.1	(2.2)	[28]	0.014	1	0.22	-2.18
**HBD**	P02042	18.2	(2.3)	[10]	16.6	(1.9)	[26]	0.068	1	0.33	-1.60
**HBG2**	P69892	16.3	(1.5)	[9]	15.0	(2.0)	[18]	0.075	1	0.40	-1.32
**HEXA**	H3BP20	14.5	(0.9)	[8]	14.8	(0.8)	[28]	0.411	1	1.22	0.29
**HEXB**	P07686	14.4	(1.3)	[8]	15.1	(0.7)	[28]	0.19	1	1.59	0.67
**HGFAC**	D6RAR4	14.4	(0.6)	[10]	14.1	(0.5)	[19]	0.213	1	0.82	-0.29
**HIST1H2BK**	O60814	14.7	(1.0)	[6]	15.1	(1.0)	[22]	0.37	1	1.34	0.42
**HIST1H4A**	P62805	14.8	(1.3)	[9]	14.2	(1.3)	[28]	0.245	1	0.66	-0.60
**HLA-C**	A0A140T921	14.0	(0.5)	[6]	13.6	(0.9)	[10]	0.323	1	0.78	-0.36
**HP**	P00738	17.3	(1.7)	[10]	17.1	(1.1)	[28]	0.75	1	0.88	-0.18
**HPR**	P00739	16.1	(1.4)	[10]	15.0	(1.2)	[28]	0.041	1	0.47	-1.09
**HPRT1**	P00492	14.1	(0.9)	[6]	13.5	(0.9)	[11]	0.174	1	0.63	-0.67
**HPX**	P02790	17.9	(0.7)	[10]	18.4	(0.5)	[28]	0.057	1	1.40	0.49
**HRG**	P04196	16.0	(0.7)	[10]	15.5	(0.5)	[28]	0.08	1	0.72	-0.47
**HSP90B1**	P14625	14.5	(0.3)	[6]	14.0	(0.7)	[20]	0.024	1	0.71	-0.49
**HSP90AA1**	P07900	14.4	(0.7)	[8]	14.7	(0.8)	[12]	0.46	1	1.19	0.25
**HSPA13**	P48723	13.6	(1.0)	[6]	13.2	(0.8)	[23]	0.422	1	0.77	-0.38
**HSPA1B**	A0A0G2JIW1	15.5	(1.0)	[9]	14.5	(1.2)	[15]	0.029	1	0.48	-1.06
**HSPA5**	P11021	14.5	(0.4)	[9]	14.3	(0.4)	[27]	0.352	1	0.90	-0.15
**HSPA8**	P11142	15.3	(1.1)	[10]	14.5	(1.2)	[27]	0.084	1	0.59	-0.76

Name	Uniprot ID	Control			Obstructive HC			P value	P adjusted	Fold change	Log_2(fold change)
		Mean	(SD)	[N]	Mean	(SD)	[N]				
**HSPG2**	P98160	13.6	(0.9)	[10]	14.1	(0.7)	[28]	0.146	1	1.41	0.50
**HTRA1**	Q92743	14.1	(1.0)	[9]	15	(0.8)	[28]	0.036	1	1.86	0.90
**HYOU1**	A0A087X054	14.1	(0.5)	[6]	14.2	(0.5)	[19]	0.623	1	1.09	0.12
**ICAM5**	Q9UMF0	13.3	(0.6)	[6]	12.9	(1.0)	[22]	0.17	1	0.73	-0.45
**ICOSLG**	K4DIA0	15.4	(1.1)	[10]	16.1	(1.0)	[28]	0.096	1	1.62	0.70
**IDS**	P22304	14.3	(1.2)	[7]	14.8	(0.7)	[28]	0.362	1	1.36	0.44
**IGF2**	P01344	14.6	(0.3)	[5]	14.7	(0.5)	[20]	0.452	1	1.11	0.15
**IGFALS**	P35858	15.2	(0.7)	[10]	14.5	(0.6)	[28]	0.01	1	0.62	-0.69
**IGFBP2**	P18065	14.0	(0.8)	[7]	14.2	(0.9)	[28]	0.497	1	1.19	0.25
**IGFBP5**	P24593	12.1	(1.0)	[8]	13.0	(0.8)	[24]	0.038	1	1.88	0.91
**IGFBP6**	P24592	17.1	(0.8)	[10]	17.2	(1.0)	[28]	0.689	1	1.09	0.12
**IGFBP7**	Q16270	15.9	(2.0)	[10]	17.7	(1.5)	[28]	0.02	1	3.58	1.84
**IGHA1**	A0A286YEY1	17.6	(1.1)	[10]	17.1	(0.9)	[28]	0.235	1	0.71	-0.49
**IGHA2**	A0A286YEY5	17.7	(0.9)	[10]	17.2	(0.8)	[28]	0.157	1	0.72	-0.47
**IGHD**	A0A0A0MS09	15.8	(1.1)	[8]	14.3	(1.4)	[16]	0.011	1	0.36	-1.47
**IGHG1**	P01857	19.5	(0.7)	[10]	19.3	(0.5)	[28]	0.369	1	0.86	-0.22
**IGHG2**	P01859	19.4	(0.7)	[10]	19.4	(0.5)	[28]	0.747	1	0.95	-0.07
**IGHG3**	P01860	16.6	(1.3)	[10]	16.2	(0.7)	[28]	0.292	1	0.72	-0.47
**IGHG4**	A0A286YFJ8	15.9	(0.7)	[10]	15.7	(0.8)	[27]	0.654	1	0.92	-0.12
**IGHM**	A0A1B0GUU9	17.4	(2.3)	[10]	15.2	(1.9)	[27]	0.016	1	0.22	-2.18
**IGHV1-18**	A0A0C4DH31	14.2	(0.7)	[6]	14.3	(0.5)	[15]	0.788	1	1.06	0.08

Name	Uniprot ID	Control Mean	(SD)	[N]	Obstructive HC Mean	(SD)	[N]	P value	P adjusted	Fold change	Log_2(fold change)
**IGHV1-2**	P23083	15.1	(0.5)	[9]	15.0	(0.6)	[11]	0.703	1	0.94	-0.09
**IGHV1OR15-1**	A0A075B7D0	17.0	(1.5)	[9]	16.7	(1.5)	[28]	0.573	1	0.79	-0.34
**IGHV2-26**	A0A0B4J1V2	13.1	(0.6)	[8]	13.1	(0.7)	[21]	0.808	1	0.95	-0.07
**IGHV2-5**	P01817	12.7	(0.7)	[5]	12.7	(1.6)	[11]	0.99	1	0.99	-0.01
**IGHV3-15**	A0A0B4J1V0	16.0	(0.4)	[10]	15.8	(0.6)	[28]	0.416	1	0.90	-0.15
**IGHV3-30**	P01768	16.9	(0.2)	[10]	16.8	(0.5)	[28]	0.34	1	0.92	-0.12
**IGHV3-38**	A0A0C4DH36	15.4	(0.7)	[10]	15.1	(0.6)	[27]	0.246	1	0.82	-0.29
**IGHV3-49**	A0A0A0MS15	16.9	(0.8)	[10]	16.3	(1.1)	[28]	0.092	1	0.67	-0.58
**IGHV3-64D**	A0A0J9YX35	15.9	(0.4)	[10]	16.1	(0.7)	[27]	0.295	1	1.15	0.20
**IGHV3-7**	P01780	17.0	(0.2)	[10]	17.1	(0.6)	[28]	0.722	1	1.03	0.04
**IGHV3-72**	A0A0B4J1Y9	16.3	(0.6)	[10]	16.1	(0.7)	[28]	0.322	1	0.85	-0.23
**IGHV3OR16-12**	A0A075B7B8	13.1	(0.4)	[7]	12.8	(0.6)	[13]	0.105	1	0.77	-0.38
**IGHV3OR16-9**	A0A0B4J2B5	19.8	(0.8)	[10]	19.4	(0.7)	[28]	0.194	1	0.77	-0.38
**IGHV4-34**	P06331	15.1	(0.6)	[10]	14.7	(0.8)	[28]	0.089	1	0.73	-0.45
**IGHV5-51**	A0A0C4DH38	15.8	(0.4)	[10]	16.0	(0.6)	[28]	0.226	1	1.16	0.21
**IGKC**	P01834	20.1	(0.8)	[10]	19.6	(0.5)	[28]	0.114	1	0.72	-0.47
**IGKV1-12**	A0A0C4DH73	16.9	(1.0)	[10]	16.5	(0.6)	[28]	0.185	1	0.72	-0.47
**IGKV1-16**	P04430	14.4	(0.6)	[7]	14.6	(0.6)	[11]	0.501	1	1.15	0.20
**IGKV1-17**	P01599	15.6	(0.6)	[10]	15.6	(0.6)	[28]	0.874	1	0.98	-0.03
**IGKV1-5**	P01602	16.4	(0.6)	[10]	16.2	(0.6)	[28]	0.337	1	0.87	-0.20
**IGKV1-8**	A0A0C4DH67	16.3	(1.2)	[10]	15.9	(0.6)	[28]	0.282	1	0.73	-0.45

Name	Uniprot ID	Control Mean	Control (SD)	Control [N]	Obstructive HC Mean	Obstructive HC (SD)	Obstructive HC [N]	P value	P adjusted	Fold change	Log_2(fold change)
**IGKV1D-33**	P01593	17.7	(1.1)	[10]	16.9	(0.7)	[28]	0.042	1	0.55	-0.86
**IGKV1D-37**	A0A075B6S9	16.7	(1.1)	[8]	15.9	(1.3)	[10]	0.145	1	0.56	-0.84
**IGKV2-28**	A0A075B6P5	16.0	(0.7)	[10]	16.0	(0.9)	[28]	0.972	1	0.99	-0.01
**IGKV2-29**	A2NJV5	16.3	(0.7)	[10]	16.0	(0.7)	[28]	0.313	1	0.83	-0.27
**IGKV2-40**	A0A087WW87	13.5	(0.6)	[8]	13.8	(1.5)	[15]	0.38	1	1.32	0.40
**IGKV2D-24**	A0A075B6R9	16.9	(1.2)	[10]	16.3	(0.8)	[28]	0.178	1	0.66	-0.60
**IGKV2D-29**	A0A075B6S2	13.9	(0.6)	[6]	14.6	(0.7)	[17]	0.06	1	1.57	0.65
**IGKV3-15**	P01624	17.4	(0.7)	[10]	17.4	(0.9)	[28]	0.944	1	1.01	0.01
**IGKV3-20**	P01619	18.3	(0.8)	[10]	17.9	(0.7)	[28]	0.195	1	0.77	-0.38
**IGKV3-7**	A0A075B6H7	17.3	(1.4)	[9]	19.0	(0.9)	[26]	0.007	1	3.23	1.69
**IGKV3D-11**	A0A0A0MRZ8	17.6	(0.5)	[10]	17.2	(0.6)	[28]	0.056	1	0.76	-0.40
**IGKV3D-15**	A0A087WSY6	17.5	(0.9)	[9]	17.0	(1.1)	[20]	0.229	1	0.72	-0.47
**IGKV3D-20**	A0A0C4DH25	18.4	(0.5)	[10]	17.7	(0.7)	[28]	0.003	1	0.64	-0.64
**IGKV4-1**	P06312	17.4	(0.5)	[10]	16.9	(0.6)	[28]	0.037	1	0.74	-0.43
**IGLC3**	P0DOY3	19.9	(0.5)	[10]	20.0	(0.8)	[28]	0.586	1	1.08	0.11
**IGLL5**	A0A0B4J231	18.0	(0.8)	[10]	17.6	(0.6)	[28]	0.145	1	0.75	-0.42
**IGLV1-47**	P01700	16.2	(0.4)	[10]	16.4	(0.9)	[28]	0.371	1	1.15	0.20
**IGLV1-51**	P01701	16.0	(0.9)	[8]	14.8	(2.3)	[15]	0.094	1	0.45	-1.15
**IGLV3-10**	A0A075B6K4	15.5	(0.7)	[8]	15.1	(0.6)	[22]	0.267	1	0.80	-0.32
**IGLV3-19**	P01714	14.3	(0.4)	[9]	14.5	(1.1)	[19]	0.567	1	1.12	0.16
**IGLV3-21**	P80748	15.2	(1.0)	[9]	14.8	(1.1)	[24]	0.349	1	0.76	-0.40

Name	Uniprot ID	Control Mean	(SD)	[N]	Obstructive HC Mean	(SD)	[N]	P value	P adjusted	Fold change	Log_2(fold change)
**IGLV3-25**	P01717	14.3	(1.0)	[10]	14.7	(0.6)	[26]	0.366	1	1.25	0.32
**IGLV3-9**	A0A075B6K5	15.2	(1.4)	[10]	14.5	(0.7)	[28]	0.133	1	0.60	-0.74
**IGLV6-57**	P01721	14.6	(0.8)	[8]	13.8	(0.9)	[20]	0.026	1	0.57	-0.81
**IGLV7-46**	A0A075B6I9	15.5	(0.5)	[7]	15.7	(1.4)	[12]	0.53	1	1.21	0.28
**IGLV8-61**	A0A075B6I0	15.8	(0.8)	[9]	14.9	(0.9)	[22]	0.024	1	0.56	-0.84
**IGSF21**	Q96ID5	14.9	(0.5)	[6]	14.8	(0.7)	[20]	0.592	1	0.91	-0.14
**IGSF8**	Q969P0	15.4	(1.2)	[10]	16.3	(0.7)	[28]	0.05	1	1.84	0.88
**IL31RA**	Q8NI17	22.5	(1.1)	[7]	24.3	(1.3)	[23]	0.003	1	3.59	1.84
**IL6ST**	P40189	14.5	(0.7)	[8]	14.6	(0.5)	[27]	0.802	1	1.05	0.07
**IMPAD1**	Q9NX62	14.1	(0.7)	[7]	14.0	(0.7)	[26]	0.699	1	0.92	-0.12
**ISLR**	O14498	14.9	(0.7)	[9]	14.6	(0.7)	[22]	0.272	1	0.80	-0.32
**ISLR2**	Q6UXK2	13.4	(2.5)	[5]	16.1	(2.7)	[10]	0.088	1	6.50	2.70
**ITIH1**	P19827	16.0	(0.9)	[10]	15.1	(0.6)	[28]	0.012	1	0.52	-0.94
**ITIH2**	P19823	16.0	(1.0)	[10]	15.1	(0.6)	[28]	0.023	1	0.55	-0.86
**ITIH3**	Q06033	14.8	(0.9)	[9]	13.7	(0.7)	[17]	0.007	1	0.45	-1.15
**ITIH4**	Q14624	16.6	(0.8)	[10]	15.7	(0.4)	[28]	0.005	1	0.54	-0.89
**ITIH5**	C9J2H1	13.3	(0.5)	[8]	14.0	(0.6)	[26]	0.008	1	1.61	0.69
**ITM2B**	Q9Y287	14.1	(1.3)	[8]	14.1	(2.1)	[26]	0.955	1	1.03	0.04
**ITPR2**	Q14571	18.1	(1.1)	[10]	18.9	(0.9)	[28]	0.06	1	1.71	0.77
**JCHAIN**	D6RD17	16.6	(1.8)	[9]	14.9	(1.5)	[25]	0.023	1	0.30	-1.74
**KIAA1549L**	H0YDE5	14.4	(0.9)	[10]	14.2	(1.0)	[28]	0.629	1	0.89	-0.17

Name	Uniprot ID	Control Mean	Control (SD)	Control [N]	Obstructive HC Mean	Obstructive HC (SD)	Obstructive HC [N]	P value	P adjusted	Fold change	Log_2(fold change)
**KLK6**	Q92876	16.4	(1.4)	[10]	17.9	(0.9)	[28]	0.011	1	2.76	1.46
**KLKB1**	H0YAC1	14.6	(1.1)	[10]	13.8	(0.6)	[28]	0.052	1	0.59	-0.76
**KNG1**	P01042	17.1	(0.9)	[10]	16.8	(0.6)	[28]	0.251	1	0.78	-0.36
**KRT1**	P04264	14.9	(1.5)	[8]	15.7	(1.6)	[28]	0.203	1	1.76	0.82
**KRT10**	P13645	14.1	(0.5)	[5]	14.5	(1.5)	[23]	0.367	1	1.30	0.38
**KRT2**	P35908	13.7	(1.9)	[9]	14.9	(1.8)	[27]	0.115	1	2.29	1.20
**KRT6A**	P02538	13.8	(1.2)	[5]	13.5	(2.0)	[23]	0.752	1	0.86	-0.22
**KRT77**	Q7Z794	14.7	(1.0)	[6]	15.4	(1.8)	[26]	0.200	1	1.66	0.73
**KRT9**	P35527	14.4	(1.1)	[7]	14.5	(1.4)	[23]	0.736	1	1.13	0.18
**L1CAM**	P32004	13.5	(0.6)	[6]	12.9	(0.9)	[24]	0.068	1	0.65	-0.62
**LAMA2**	A0A087WX80	11.5	(1.0)	[6]	11.6	(1.3)	[10]	0.805	1	1.11	0.15
**LAMC1**	P11047	14.7	(0.3)	[5]	15.3	(1.6)	[12]	0.236	1	1.52	0.60
**LAMP1**	P11279	13.8	(0.5)	[5]	13.4	(0.5)	[22]	0.232	1	0.78	-0.36
**LAMP2**	P13473	15.5	(1.1)	[10]	16.2	(0.8)	[28]	0.078	1	1.66	0.73
**LBP**	P18428	14.0	(0.5)	[7]	13.9	(0.6)	[21]	0.393	1	0.87	-0.20
**LCAT**	P04180	14.9	(0.4)	[8]	14.9	(0.6)	[19]	0.968	1	1.01	0.01
**LCP1**	P13796	14.6	(0.7)	[7]	13.7	(0.7)	[18]	0.012	1	0.53	-0.92
**LDHA**	P00338	15.0	(1.4)	[10]	13.7	(0.7)	[28]	0.015	1	0.40	-1.32
**LDHB**	P07195	15.4	(0.8)	[10]	14.9	(0.6)	[28]	0.06	1	0.68	-0.56
**LGALS1**	P09382	14.8	(0.6)	[10]	14.3	(0.5)	[23]	0.063	1	0.74	-0.43
**LGALS3**	P17931	13.2	(0.9)	[5]	12.4	(0.5)	[10]	0.108	1	0.55	-0.86

Name	Uniprot ID	Control Mean	Control (SD)	Control [N]	Obstructive HC Mean	Obstructive HC (SD)	Obstructive HC [N]	P value	P adjusted	Fold change	Log_2(fold change)
**LGALS3BP**	Q08380	16.3	(1.1)	[10]	17.3	(0.7)	[28]	0.033	1	1.89	0.92
**LIAS**	A0A1W2PNQ5	14.5	(1.3)	[7]	14.8	(0.7)	[12]	0.651	1	1.19	0.25
**LMAN2**	D6RBV2	14.9	(0.6)	[9]	15.3	(0.6)	[28]	0.069	1	1.37	0.45
**LRG1**	P02750	16.6	(0.3)	[10]	16.3	(0.5)	[28]	0.009	1	0.78	-0.36
**LRP1**	Q07954	13.8	(0.5)	[7]	13.7	(0.6)	[23]	0.768	1	0.95	-0.07
**LRRC4B**	Q9NT99	14.6	(0.8)	[8]	14.7	(0.7)	[28]	0.905	1	1.03	0.04
**LSAMP**	H3BLU2	15.1	(0.9)	[10]	15.2	(0.6)	[28]	0.729	1	1.08	0.11
**LTBP2**	G3V3X5	12.8	(0.3)	[6]	12.2	(0.4)	[13]	0.005	1	0.66	-0.60
**LTF**	E7EQB2	14.6	(2.4)	[8]	16.4	(1.9)	[25]	0.084	1	3.45	1.79
**LUM**	P51884	15.8	(0.6)	[10]	15.4	(0.5)	[28]	0.034	1	0.73	-0.45
**LY6H**	O94772	11.0	(0.8)	[6]	11.9	(1.2)	[16]	0.067	1	1.89	0.92
**LYVE1**	Q9Y5Y7	14.8	(0.8)	[10]	14.5	(0.7)	[28]	0.415	1	0.85	-0.23
**LYZ**	A0A0B4J259	16.3	(1.2)	[10]	16.8	(0.6)	[28]	0.270	1	1.38	0.46
**MAN1A1**	P33908	14.3	(0.4)	[10]	14.3	(0.4)	[26]	0.687	1	1.05	0.07
**MAN1C1**	Q9NR34	13.4	(0.8)	[7]	14.0	(0.7)	[24]	0.095	1	1.53	0.61
**MAN2A2**	P49641	14.1	(0.7)	[8]	14.0	(0.6)	[24]	0.952	1	0.99	-0.01
**MANBA**	O00462	14.0	(0.9)	[5]	13.8	(0.6)	[24]	0.754	1	0.90	-0.15
**MARCKS**	P29966	12.3	(0.6)	[8]	11.9	(0.7)	[24]	0.181	1	0.78	-0.36
**MASP1**	P48740	13.4	(0.5)	[10]	14.1	(0.9)	[21]	0.014	1	1.60	0.68
**MCAM**	P43121	14.7	(0.7)	[10]	14.5	(0.9)	[28]	0.419	1	0.85	-0.23
**MDH1**	P40925	15.9	(0.7)	[10]	15.7	(0.7)	[28]	0.267	1	0.82	-0.29

Name	Uniprot ID	Control Mean	Control (SD)	Control [N]	Obstructive HC Mean	Obstructive HC (SD)	Obstructive HC [N]	P value	P adjusted	Fold change	Log_2(fold change)
**MDH2**	P40926	14.6	(1.0)	[5]	14.3	(1.2)	[11]	0.649	1	0.82	-0.29
**MEGF8**	Q7Z7M0	14.3	(0.7)	[10]	14.3	(0.8)	[26]	0.918	1	1.02	0.03
**MFAP4**	K7ES70	14.5	(0.9)	[7]	14.4	(0.9)	[27]	0.688	1	0.89	-0.17
**MGP**	P08493	15.0	(1.1)	[10]	14.8	(1.0)	[26]	0.687	1	0.89	-0.17
**MIF**	P14174	16.1	(0.7)	[9]	15.6	(0.7)	[24]	0.108	1	0.71	-0.49
**MINPP1**	Q9UNW1	15.2	(0.3)	[6]	14.5	(0.8)	[11]	0.026	1	0.62	-0.69
**MMP2**	P08253	14.8	(0.8)	[10]	15.1	(0.5)	[28]	0.206	1	1.30	0.38
**MOG**	A0A0G2JHA9	14.0	(1.0)	[8]	14.8	(0.8)	[27]	0.061	1	1.76	0.82
**MSN**	P26038	13.1	(0.6)	[8]	13.0	(1.1)	[16]	0.694	1	0.91	-0.14
**MST1**	G3XAK1	13.8	(0.7)	[6]	12.8	(0.6)	[14]	0.023	1	0.52	-0.94
**NBL1**	A0A087WTY6	17.7	(0.7)	[10]	18.1	(0.6)	[28]	0.118	1	1.34	0.42
**NCAM1**	P13591	15.6	(0.7)	[10]	16.2	(0.5)	[28]	0.037	1	1.45	0.54
**NCAM2**	H9KV31	14.9	(1.0)	[10]	15.7	(0.7)	[28]	0.039	1	1.75	0.81
**NCAN**	O14594	15.0	(0.9)	[10]	15.4	(0.6)	[28]	0.183	1	1.34	0.42
**NDRG2**	Q9UN36-2	13.6	(0.4)	[8]	13.5	(1.1)	[16]	0.751	1	0.93	-0.10
**NECTIN1**	Q15223	14.3	(0.6)	[7]	14.5	(0.6)	[26]	0.414	1	1.16	0.21
**NEGR1**	Q7Z3B1	15.6	(1.0)	[10]	15.3	(1.0)	[28]	0.449	1	0.82	-0.29
**NELL2**	F8VVB6	15.2	(0.9)	[10]	15.4	(0.8)	[28]	0.471	1	1.18	0.24
**NEO1**	Q92859	14.6	(1.0)	[10]	15.2	(0.5)	[28]	0.129	1	1.47	0.56
**NFASC**	O94856	14.6	(1.0)	[9]	15.0	(0.5)	[28]	0.276	1	1.31	0.39
**NID1**	P14543	13.9	(0.4)	[5]	13.6	(0.4)	[15]	0.296	1	0.86	-0.22

Name	Uniprot ID	Control Mean	Control (SD)	Control [N]	Obstructive HC Mean	Obstructive HC (SD)	Obstructive HC [N]	P value	P adjusted	Fold change	Log_2(fold change)
**NID2**	Q14112	14.5	(0.7)	[8]	14.4	(0.7)	[26]	0.748	1	0.94	-0.09
**NLGN4X**	A0A0A0MTH0	11.8	(1.0)	[5]	12.3	(1.5)	[17]	0.402	1	1.42	0.51
**NPC2**	E7EMS2	16.0	(1.3)	[10]	17.1	(0.8)	[28]	0.020	1	2.23	1.16
**NPDC1**	Q5SPY9	13.6	(0.6)	[10]	13.8	(1.0)	[26]	0.384	1	1.17	0.23
**NPPC**	P23582	13.6	(0.7)	[10]	14.0	(0.6)	[23]	0.156	1	1.29	0.37
**NPTX1**	Q15818	15.8	(0.9)	[10]	15.6	(1.0)	[28]	0.677	1	0.91	-0.14
**NPTXR**	A0A1X7SBT7	15.4	(1.1)	[10]	15.9	(0.7)	[28]	0.23	1	1.39	0.48
**NPY**	P01303	14.2	(1.3)	[6]	14.4	(1.8)	[26]	0.867	1	1.08	0.11
**NRCAM**	C9JYY6	15.8	(0.8)	[10]	16.0	(0.7)	[28]	0.358	1	1.21	0.28
**NRN1**	A0A087WWT2	15.1	(0.5)	[8]	15.2	(0.6)	[28]	0.624	1	1.08	0.11
**NRP1**	E7EX60	13.0	(0.7)	[6]	12.9	(0.4)	[15]	0.787	1	0.95	-0.07
**NRXN1**	A0A0D9SEP4	13.9	(0.8)	[8]	14.3	(0.7)	[27]	0.214	1	1.34	0.42
**NRXN2**	G5E9G7	14.7	(1.0)	[10]	15.1	(0.6)	[28]	0.228	1	1.33	0.41
**NRXN3**	A0A0U1RQC5	14.4	(1.0)	[10]	14.5	(0.8)	[28]	0.834	1	1.05	0.07
**NTM**	Q9P121-4	14.9	(0.8)	[10]	15.4	(0.9)	[28]	0.094	1	1.46	0.55
**NTRK2**	Q16620	15.2	(0.5)	[5]	15.1	(0.6)	[16]	0.739	1	0.93	-0.10
**NUCB1**	Q02818	13.5	(1.1)	[10]	14.4	(0.7)	[28]	0.04	1	1.80	0.85
**OAF**	Q86UD1	14.6	(0.6)	[10]	14.8	(0.6)	[26]	0.368	1	1.15	0.20
**OGN**	P20774	15.8	(1.0)	[10]	15.3	(1.1)	[28]	0.233	1	0.71	-0.49
**OMD**	Q99983	13.5	(0.7)	[6]	14.1	(0.6)	[16]	0.093	1	1.52	0.60
**OMG**	P23515	15.4	(1.1)	[10]	16.2	(0.6)	[28]	0.04	1	1.81	0.86

Name	Uniprot ID	Control Mean	(SD)	[N]	Obstructive HC Mean	(SD)	[N]	P value	P adjusted	Fold change	Log_2(fold change)
**ORM1**	P02763	18.8	(0.7)	[10]	18.7	(0.5)	[28]	0.758	1	0.95	-0.07
**ORM2**	P19652	17.3	(0.5)	[10]	17.3	(0.5)	[28]	0.995	1	1.00	0.00
**PAM**	P19021	15.2	(0.6)	[10]	15.3	(0.6)	[28]	0.63	1	1.08	0.11
**PARK7**	Q99497	14.1	(1.0)	[7]	13.5	(1.0)	[15]	0.245	1	0.68	-0.56
**PCDH1**	Q08174	13.3	(0.5)	[9]	13.5	(0.7)	[17]	0.588	1	1.09	0.12
**PCDH7**	O60245	13.2	(0.7)	[5]	13.1	(1.3)	[12]	0.922	1	0.97	-0.04
**PCDHAC2**	Q9Y5I4	14.6	(0.3)	[6]	14.1	(1.5)	[12]	0.352	1	0.74	-0.43
**PCMT1**	A0A0A0MRJ6	14.1	(0.5)	[5]	14.0	(1.4)	[14]	0.816	1	0.93	-0.10
**PCOLCE**	Q15113	15.8	(0.9)	[10]	15.8	(0.7)	[28]	0.996	1	1.00	0.00
**PCSK1N**	Q9UHG2	15.7	(0.7)	[10]	15.6	(0.6)	[28]	0.84	1	0.97	-0.04
**PDGFA**	A0A0A0MSC4	15.4	(0.7)	[5]	15.6	(1.8)	[22]	0.652	1	1.17	0.23
**PDGFB**	A9UJN9	13.8	(0.6)	[6]	14.4	(1.0)	[17]	0.151	1	1.45	0.54
**PDIA3**	P30101	14.2	(0.3)	[7]	13.8	(0.5)	[21]	0.007	1	0.72	-0.47
**PEA15**	Q15121	14.5	(0.7)	[6]	14.0	(0.7)	[17]	0.158	1	0.70	-0.51
**PEBP1**	P30086	16.2	(0.7)	[10]	16.3	(0.6)	[28]	0.518	1	1.13	0.18
**PEBP4**	Q96S96	15.6	(0.9)	[9]	16.2	(0.5)	[28]	0.097	1	1.49	0.58
**PENK**	P01210	14.9	(0.9)	[10]	15.7	(0.8)	[28]	0.029	1	1.67	0.74
**PEPD**	P12955	12.8	(0.9)	[9]	12.5	(0.5)	[25]	0.437	1	0.83	-0.27
**PFN1**	P07737	15.3	(0.8)	[9]	15.1	(0.7)	[19]	0.508	1	0.86	-0.22
**PGAM1**	P18669	14.9	(0.6)	[10]	14.9	(0.8)	[28]	0.802	1	0.96	-0.06
**PGK1**	P00558	15.2	(1.0)	[10]	14.1	(1.0)	[24]	0.005	1	0.44	-1.18

Name	Uniprot ID	Control Mean	(SD)	[N]	Obstructive HC Mean	(SD)	[N]	P value	P adjusted	Fold change	Log_2(fold change)
**PGLYRP2**	Q96PD5	14.8	(0.7)	[10]	14.5	(0.5)	[25]	0.212	1	0.81	-0.30
**PI16**	Q6UXB8	13.3	(0.6)	[10]	13.8	(1.0)	[28]	0.113	1	1.36	0.44
**PIK3IP1**	Q96FE7-4	14.9	(0.8)	[8]	15.4	(0.4)	[27]	0.116	1	1.43	0.52
**PKM**	P14618	15.0	(1.0)	[10]	14.6	(0.6)	[28]	0.199	1	0.73	-0.45
**PLD3**	Q8IV08	15.1	(0.6)	[7]	15	(0.4)	[27]	0.726	1	0.94	-0.09
**PLG**	P00747	16.4	(0.8)	[10]	16.1	(0.4)	[28]	0.221	1	0.80	-0.32
**PLOD1**	Q02809	12.9	(0.6)	[5]	13.1	(0.7)	[24]	0.387	1	1.20	0.26
**PLTP**	P55058	15.4	(0.9)	[10]	15.8	(0.4)	[28]	0.153	1	1.36	0.44
**PLXDC2**	Q6UX71	14.7	(0.9)	[10]	15.4	(0.6)	[28]	0.038	1	1.60	0.68
**PLXNB2**	O15031	14.1	(0.8)	[6]	14.3	(0.7)	[24]	0.461	1	1.22	0.29
**PMFBP1**	G3V1Q7	16.2	(0.5)	[7]	16.9	(0.5)	[18]	0.003	1	1.71	0.77
**PODXL2**	Q9NZ53	12.6	(0.7)	[6]	12.1	(0.9)	[14]	0.174	1	0.70	-0.51
**POMGNT1**	Q8WZA1	14.3	(0.4)	[7]	14.3	(0.6)	[24]	0.909	1	0.99	-0.01
**PON1**	P27169	16.2	(0.9)	[9]	15.4	(0.8)	[28]	0.051	1	0.59	-0.76
**PPBP**	P02775	17.0	(1.5)	[7]	15.8	(1.3)	[12]	0.091	1	0.42	-1.25
**PPIA**	P62937	16.3	(0.9)	[10]	15.5	(0.9)	[28]	0.03	1	0.57	-0.81
**PPIB**	P23284	15.3	(1.1)	[10]	15.4	(0.7)	[28]	0.754	1	1.08	0.11
**PPIC**	P45877	15.0	(0.5)	[7]	14.8	(0.5)	[27]	0.29	1	0.84	-0.25
**PRCP**	P42785	15.2	(0.8)	[8]	15.5	(0.8)	[28]	0.339	1	1.24	0.31
**PRDX1**	Q06830	17.1	(1.5)	[10]	16.1	(1.1)	[27]	0.108	1	0.53	-0.92
**PRDX2**	P32119	17.6	(1.9)	[10]	15.7	(1.5)	[28]	0.017	1	0.28	-1.84

Name	Uniprot ID	Control			Obstructive HC			P value	P adjusted	Fold change	Log_2(fold change)
		Mean	(SD)	[N]	Mean	(SD)	[N]				
**PRDX6**	P30041	15.8	(1.5)	[10]	14.7	(1.3)	[27]	0.067	1	0.48	-1.06
**PRELP**	P51888	14.3	(0.8)	[8]	13.6	(0.9)	[21]	0.067	1	0.63	-0.67
**PRG4**	A0A0U1RR20	12.9	(1.0)	[7]	13.2	(0.6)	[11]	0.495	1	1.23	0.30
**PRKCSH**	K7ELL7	13.7	(1.1)	[6]	14.6	(0.8)	[17]	0.113	1	1.82	0.86
**PRNP**	A2A2V1	13.4	(0.8)	[10]	14.3	(0.7)	[28]	0.006	1	1.86	0.90
**PROC**	E7END6	13.6	(0.4)	[9]	13.8	(0.6)	[26]	0.231	1	1.15	0.20
**PROCR**	Q9UNN8	14.5	(0.7)	[10]	14.6	(0.7)	[28]	0.64	1	1.09	0.12
**PROS1**	P07225	15.3	(0.5)	[10]	15.6	(0.5)	[28]	0.197	1	1.20	0.26
**PROZ**	P22891	14.0	(1.0)	[8]	13.5	(0.6)	[12]	0.17	1	0.68	-0.56
**PRSS1**	E7EQ64	15.9	(1.6)	[7]	16.4	(0.9)	[18]	0.473	1	1.39	0.48
**PRSS3**	B1AN99	18.5	(1.5)	[5]	21.3	(2.3)	[16]	0.008	1	6.94	2.79
**PSAP**	C9JIZ6	14.3	(1.0)	[8]	14.7	(1.0)	[26]	0.377	1	1.29	0.37
**PSAT1**	Q9Y617	13.5	(1.0)	[6]	14.3	(0.8)	[23]	0.122	1	1.68	0.75
**PTGDS**	P41222	20.1	(0.7)	[10]	20.8	(0.5)	[28]	0.018	1	1.57	0.65
**PTPRD**	P23468	14.3	(0.9)	[9]	14.9	(0.6)	[28]	0.097	1	1.49	0.58
**PTPRF**	P10586	13.8	(0.7)	[6]	14.1	(1.5)	[15]	0.456	1	1.29	0.37
**PTPRG**	P23470	15.0	(0.8)	[10]	15.0	(0.6)	[28]	0.993	1	1.00	0.00
**PTPRN**	Q16849	12.9	(0.8)	[5]	12.4	(2.1)	[12]	0.484	1	0.70	-0.51
**PTPRN2**	Q92932	14.8	(0.6)	[10]	15.6	(0.7)	[28]	0.004	1	1.71	0.77
**PTPRS**	Q13332	14.2	(1.0)	[10]	15	(0.6)	[28]	0.029	1	1.78	0.83
**PTPRZ1**	P23471	14.7	(1.0)	[10]	15.6	(0.8)	[28]	0.025	1	1.89	0.92

Name	Uniprot ID	Control Mean	Control (SD)	Control [N]	Obstructive HC Mean	Obstructive HC (SD)	Obstructive HC [N]	P value	P adjusted	Fold change	Log_2(fold change)
**PVALB**	B8ZZ19	14.1	(0.3)	[5]	13.5	(0.9)	[10]	0.059	1	0.64	-0.64
**QDPR**	P09417	14.8	(1.1)	[8]	15	(1.0)	[26]	0.652	1	1.15	0.20
**QPCT**	Q16769	13.7	(0.6)	[10]	14.1	(0.7)	[28]	0.06	1	1.36	0.44
**QSOX1**	O00391	14.1	(0.6)	[10]	14.6	(0.5)	[28]	0.045	1	1.40	0.49
**RARRES2**	Q99969	15.0	(1.1)	[10]	16.0	(1.0)	[28]	0.016	1	2.09	1.06
**RBP4**	P02753	15.7	(0.6)	[10]	16.4	(0.6)	[28]	0.005	1	1.62	0.70
**RELN**	J3KQ66	14.8	(1.0)	[8]	14.5	(0.8)	[18]	0.507	1	0.82	-0.29
**RGMB**	J3KNF6	14.7	(1.0)	[10]	14.8	(0.9)	[24]	0.838	1	1.05	0.07
**RNASE1**	P07998	11.6	(1.0)	[9]	12.5	(1.4)	[28]	0.044	1	1.87	0.90
**RNASET2**	A0A087WZM2	14.4	(1.4)	[9]	15.4	(0.7)	[28]	0.079	1	1.92	0.94
**ROBO1**	Q9Y6N7	13.8	(0.6)	[6]	13.7	(0.8)	[20]	0.747	1	0.93	-0.10
**RTN4R**	H7C0V4	13.9	(0.6)	[5]	13.1	(1.7)	[14]	0.139	1	0.57	-0.81
**RTN4RL2**	Q86UN3	14.6	(1.3)	[8]	14.5	(0.7)	[26]	0.953	1	0.98	-0.03
**S100A1**	P23297	13.7	(1.0)	[5]	13.5	(1.0)	[15]	0.801	1	0.91	-0.14
**S100A8**	P05109	15.9	(1.9)	[8]	14.7	(1.2)	[10]	0.138	1	0.42	-1.25
**S100A9**	P06702	15.6	(1.7)	[8]	14.6	(1.1)	[11]	0.203	1	0.53	-0.92
**S100B**	P04271	13.8	(0.8)	[7]	15.1	(1.5)	[27]	0.008	1	2.44	1.29
**SCG2**	P13521	14.3	(0.8)	[10]	14.9	(0.7)	[28]	0.036	1	1.58	0.66
**SCG3**	Q8WXD2	15.9	(0.6)	[10]	16.1	(0.6)	[28]	0.491	1	1.12	0.16
**SCG5**	P05408-2	16.0	(0.7)	[10]	16.5	(0.6)	[28]	0.054	1	1.46	0.55
**SCRG1**	O75711	16.8	(0.9)	[9]	17.3	(1.0)	[28]	0.198	1	1.38	0.46

Name	Uniprot ID	Control Mean	Control (SD)	Control [N]	Obstructive HC Mean	Obstructive HC (SD)	Obstructive HC [N]	P value	P adjusted	Fold change	Log_2(fold change)
**SDF4**	Q9BRK5	13.1	(0.6)	[8]	14.1	(0.9)	[22]	0.005	1	1.94	0.96
**SEC23IP**	Q9Y6Y8	14.3	(0.8)	[8]	14.7	(0.7)	[26]	0.264	1	1.28	0.36
**SELENBP1**	Q13228	15.8	(1.0)	[10]	15.0	(0.6)	[28]	0.031	1	0.57	-0.81
**SELENOP**	A0A182DWH7	15.5	(0.4)	[9]	15.8	(0.6)	[25]	0.128	1	1.20	0.26
**SELL**	P14151	15.4	(0.7)	[10]	15.1	(0.4)	[26]	0.235	1	0.81	-0.30
**SEMA3G**	Q9NS98	13.6	(0.4)	[5]	12.8	(1.0)	[12]	0.032	1	0.59	-0.76
**SEMA4B**	J3KNP4	13.4	(0.4)	[5]	13.7	(0.7)	[21]	0.228	1	1.24	0.31
**SEMA7A**	O75326	14.5	(1.0)	[10]	15.0	(0.8)	[28]	0.125	1	1.46	0.55
**SERPINA1**	P01009	18.4	(0.3)	[10]	18.0	(0.4)	[28]	0.011	1	0.77	-0.38
**SERPINA10**	G3V2W1	12.9	(1.5)	[5]	12.1	(1.1)	[10]	0.329	1	0.58	-0.79
**SERPINA3**	P01011	17.0	(0.6)	[10]	17.1	(0.5)	[28]	0.659	1	1.07	0.10
**SERPINA4**	P29622	15.0	(0.5)	[10]	14.9	(0.3)	[28]	0.675	1	0.95	-0.07
**SERPINA5**	P05154	14.1	(0.6)	[10]	14.1	(1.1)	[28]	0.972	1	0.99	-0.01
**SERPINA6**	P08185	15.3	(0.2)	[10]	15.2	(0.5)	[28]	0.473	1	0.94	-0.09
**SERPINA7**	P05543	14.7	(0.3)	[10]	14.9	(0.4)	[28]	0.208	1	1.12	0.16
**SERPINC1**	P01008	16.7	(0.4)	[10]	16.7	(0.3)	[28]	0.775	1	0.97	-0.04
**SERPIND1**	P05546	15.5	(0.3)	[10]	15.4	(0.3)	[28]	0.36	1	0.93	-0.10
**SERPINF1**	P36955	17.4	(1.2)	[10]	18.1	(0.9)	[28]	0.106	1	1.67	0.74
**SERPINF2**	P08697	16.3	(0.4)	[10]	16.1	(0.3)	[28]	0.188	1	0.87	-0.20
**SERPING1**	P05155	16.5	(0.2)	[10]	16.3	(0.3)	[28]	0.075	1	0.89	-0.17
**SERPINI1**	Q99574	14.7	(1.2)	[9]	15.1	(0.7)	[28]	0.333	1	1.33	0.41

Name	Uniprot ID	Control Mean	Control (SD)	Control [N]	Obstructive HC Mean	Obstructive HC (SD)	Obstructive HC [N]	P value	P adjusted	Fold change	Log_2(fold change)
**SEZ6**	Q53EL9	13.9	(0.5)	[8]	13.9	(0.6)	[16]	0.957	1	0.99	-0.01
**SEZ6L**	B0QYH4	14.5	(0.4)	[8]	14.3	(0.6)	[27]	0.334	1	0.87	-0.20
**SEZ6L2**	A0A087WYL5	15.3	(0.2)	[8]	15.3	(0.6)	[28]	0.898	1	1.01	0.01
**SH3BGRL**	O75368	13.8	(0.7)	[7]	13.0	(0.6)	[16]	0.023	1	0.59	-0.76
**SH3BGRL3**	Q5T123	14.0	(0.6)	[7]	13.5	(0.9)	[15]	0.142	1	0.71	-0.49
**SHBG**	I3L145	14.7	(1.0)	[7]	13.8	(0.6)	[25]	0.059	1	0.56	-0.84
**SHISA6**	Q6ZSJ9	14.5	(0.4)	[5]	14.0	(0.7)	[22]	0.101	1	0.73	-0.45
**SIAE**	Q9HAT2	15.1	(1.5)	[6]	15.6	(1.2)	[27]	0.489	1	1.39	0.48
**SIRPA**	P78324	14.8	(1.0)	[10]	15.2	(0.6)	[28]	0.207	1	1.35	0.43
**SKP1**	E5RJR5	14.1	(0.7)	[9]	14.2	(0.6)	[27]	0.531	1	1.12	0.16
**SLC39A10**	Q9ULF5	13.9	(0.6)	[6]	13.1	(1.0)	[12]	0.054	1	0.59	-0.76
**SLC3A2**	F5GZS6	13.1	(0.4)	[5]	13.0	(0.6)	[21]	0.543	1	0.90	-0.15
**SLITRK1**	Q96PX8	14.6	(1.3)	[7]	13.7	(1.0)	[13]	0.133	1	0.52	-0.94
**SLITRK4**	Q8IW52	13.1	(0.9)	[8]	13.3	(0.9)	[20]	0.649	1	1.13	0.18
**SOD1**	P00441	16.1	(0.4)	[10]	16.2	(0.5)	[28]	0.506	1	1.08	0.11
**SOD2**	P04179	13.6	(0.5)	[10]	13.7	(0.5)	[28]	0.515	1	1.08	0.11
**SOD3**	P08294	15.3	(1.4)	[10]	16.4	(1.2)	[28]	0.044	1	2.11	1.08
**SORCS3**	Q9UPU3	13.5	(1.0)	[9]	13.8	(1.0)	[25]	0.465	1	1.22	0.29
**SORT1**	Q99523	14.0	(0.8)	[6]	14.3	(0.7)	[26]	0.417	1	1.23	0.30
**SPARC**	P09486	15.1	(1.8)	[10]	17.1	(1.5)	[28]	0.007	1	4.05	2.02
**SPARCL1**	Q14515	14.8	(0.6)	[10]	15.5	(0.4)	[28]	0.004	1	1.58	0.66

Name	Uniprot ID	Control Mean	Control (SD)	Control [N]	Obstructive HC Mean	Obstructive HC (SD)	Obstructive HC [N]	P value	P adjusted	Fold change	Log_2(fold change)
**SPINT2**	K7EM91	13.3	(0.6)	[5]	12.9	(0.8)	[18]	0.175	1	0.72	-0.47
**SPOCK1**	Q08629	13.9	(0.9)	[8]	14.5	(0.7)	[27]	0.115	1	1.54	0.62
**SPOCK2**	Q92563	13.9	(0.3)	[5]	14.0	(0.6)	[22]	0.635	1	1.07	0.10
**SPOCK3**	Q9BQ16	14.9	(1.0)	[8]	15.6	(0.9)	[28]	0.116	1	1.62	0.70
**SPON1**	Q9HCB6	14.5	(0.5)	[8]	14.5	(0.9)	[24]	0.901	1	1.02	0.03
**SPP1**	P10451	15.6	(0.6)	[10]	16.3	(0.6)	[28]	0.005	1	1.60	0.68
**SULF2**	Q8IWU5	14.3	(0.7)	[10]	14.9	(0.9)	[28]	0.042	1	1.53	0.61
**SUSD5**	O60279	12.6	(0.8)	[7]	13.1	(0.7)	[22]	0.154	1	1.43	0.52
**SYNE3**	G3V533	15.0	(0.7)	[7]	14.6	(0.7)	[18]	0.197	1	0.74	-0.43
**SYT2**	Q8N9I0	17.3	(1.1)	[9]	17.7	(1.0)	[22]	0.336	1	1.33	0.41
**SAA4**	P35542	15.6	(0.9)	[10]	15.0	(0.6)	[28]	0.065	1	0.66	-0.60
**TAGLN**	Q01995	14.7	(0.7)	[8]	13.9	(0.6)	[16]	0.015	1	0.55	-0.86
**TALDO1**	P37837	15.1	(1.5)	[9]	14.0	(1.7)	[15]	0.141	1	0.49	-1.03
**TCN2**	B5MBX2	15.6	(0.9)	[8]	15.8	(1.9)	[24]	0.665	1	1.16	0.21
**TF**	P02787	17.7	(0.5)	[10]	17.9	(0.3)	[28]	0.241	1	1.14	0.19
**TGFBI**	Q15582	14.5	(0.8)	[10]	14.7	(0.4)	[28]	0.548	1	1.12	0.16
**TGOLN2**	F8W8W7	12.2	(0.9)	[10]	12.7	(1.2)	[28]	0.178	1	1.44	0.53
**THBS2**	P35442	14.0	(0.4)	[6]	13.9	(0.6)	[21]	0.569	1	0.92	-0.12
**THY1**	E9PIM6	16.9	(0.9)	[10]	17.8	(0.6)	[28]	0.021	1	1.77	0.82
**TIMP1**	P01033	16.3	(1.8)	[10]	16.5	(0.7)	[23]	0.757	1	1.14	0.19
**TIMP2**	P16035	15.4	(0.9)	[8]	16.2	(0.9)	[24]	0.047	1	1.76	0.82

Name	Uniprot ID	Control Mean	Control (SD)	Control [N]	Obstructive HC Mean	Obstructive HC (SD)	Obstructive HC [N]	P value	P adjusted	Fold change	Log_2(fold change)
**TKT**	P29401	14.7	(0.6)	[7]	14.1	(1.0)	[15]	0.099	1	0.67	-0.58
**TMEM132A**	Q24JP5	14.7	(0.4)	[6]	14.1	(0.8)	[21]	0.027	1	0.67	-0.58
**TNFRSF21**	O75509	14.9	(0.7)	[5]	14.8	(0.5)	[26]	0.672	1	0.90	-0.15
**TNR**	Q92752	12.8	(1.2)	[5]	13.8	(0.5)	[23]	0.141	1	2.00	1.00
**TNXB**	A0A140TA41	13.5	(0.6)	[7]	13.2	(0.8)	[18]	0.375	1	0.84	-0.25
**TPI1**	P60174	15.2	(1.1)	[10]	14.7	(0.8)	[28]	0.175	1	0.69	-0.54
**TPP1**	O14773	15.0	(1.0)	[8]	15.3	(1.0)	[28]	0.43	1	1.26	0.33
**TPP2**	P29144	14.0	(0.9)	[10]	14.0	(0.9)	[28]	0.944	1	1.02	0.03
**TREM2**	Q9NZC2	13.5	(0.6)	[8]	13.9	(1.1)	[23]	0.248	1	1.28	0.36
**TTR**	P02766	18.6	(1.4)	[10]	20.1	(1.4)	[28]	0.01	1	2.83	1.50
**TUBA1B**	P68363	16.0	(1.5)	[9]	16.1	(1.7)	[27]	0.883	1	1.06	0.08
**TUBB**	P07437	16.6	(1.0)	[7]	16.1	(0.8)	[21]	0.311	1	0.72	-0.47
**TUBB4B**	P68371	15.9	(1.1)	[9]	15.7	(1.3)	[26]	0.659	1	0.87	-0.20
**TXN**	P10599	17.0	(0.9)	[10]	16.7	(0.7)	[27]	0.314	1	0.80	-0.32
**TXNDC17**	Q9BRA2	14.1	(0.4)	[6]	14	(0.4)	[14]	0.661	1	0.94	-0.09
**UBC**	F5H265	15.9	(0.8)	[10]	16.4	(0.7)	[28]	0.061	1	1.47	0.56
**UBE2N**	P61088	14.1	(1.2)	[7]	13.3	(0.8)	[12]	0.13	1	0.55	-0.86
**VASN**	Q6EMK4	14.7	(0.4)	[10]	14.8	(0.5)	[28]	0.589	1	1.06	0.08
**VCAM1**	P19320	13.3	(0.7)	[8]	13.8	(0.5)	[19]	0.088	1	1.41	0.50
**VCAN**	P13611	14.3	(0.3)	[8]	14.1	(0.6)	[28]	0.331	1	0.90	-0.15
**VGF**	O15240	15.4	(1.2)	[10]	15.6	(0.8)	[28]	0.715	1	1.11	0.15

Name	Uniprot ID	Control Mean	(SD)	[N]	Obstructive HC Mean	(SD)	[N]	P value	P adjusted	Fold change	Log_2(fold change)
**VIP**	P01282	18.0	(0.3)	[10]	17.7	(0.5)	[28]	0.082	1	0.84	-0.25
**VSIG4**	Q9Y279	13.8	(1.3)	[7]	13.0	(0.7)	[27]	0.155	1	0.57	-0.81
**VSTM2A**	B5MCX6	14.0	(0.7)	[9]	14.5	(1.4)	[28]	0.151	1	1.43	0.52
**VSTM2B**	A6NLU5	12.7	(0.6)	[5]	14.1	(0.9)	[27]	0.004	1	2.59	1.37
**VTN**	P04004	17.3	(0.8)	[10]	17.0	(0.4)	[28]	0.43	1	0.86	-0.22
**VWF**	P04275	13.9	(0.7)	[8]	14.4	(2.1)	[10]	0.557	1	1.34	0.42
**WFIKKN2**	C9J6G4	15.2	(1.7)	[9]	15.7	(0.9)	[28]	0.418	1	1.42	0.51
**YWHAE**	P62258	14.7	(1.0)	[9]	14.1	(1.3)	[27]	0.167	1	0.67	-0.58
**YWHAG**	P61981	12.4	(0.6)	[8]	13.8	(1.7)	[18]	0.006	1	2.60	1.38
**YWHAQ**	P27348	13.4	(1.1)	[8]	13.9	(1.1)	[23]	0.313	1	1.39	0.48
**YWHAZ**	P63104	14.9	(0.8)	[10]	14.3	(1.0)	[28]	0.093	1	0.69	-0.54
**ZNF511-PRAP1**	H7BY64	14.6	(1.2)	[5]	14.5	(1.1)	[26]	0.826	1	0.91	-0.14
