# Supplementary material for: Ventricular CSF proteomic profiles and predictors of surgical treatment outcome in chronic hydrocephalus
Source: Acta Neurochir (Wien). 2023 Oct 19;165(12):4059–70. doi: 10.1007/s00701-023-05832-y (PMC10739511; doi:10.1007/s00701-023-05832-y)
Supplement: Supplementary file 4 — Supplementary file4 (PDF 1102 KB) [file 701_2023_5832_MOESM4_ESM.pdf]

Supplemental file 4

Communicating HC compared to obstructive HC

All proteins identified by mass-spectrometry-based proteomics in at least 10 samples from patients with communicating hydrocephalus (HC) and 10 samples from patients with obstructive HC. The protein that differed significantly between the two groups is highlighted in green.

		Communicating HC			Obstructive HC						
**Name**	**Uniprot ID**	**Mean**	**(SD)**	**[N]**	**Mean**	**(SD)**	**[N]**	**P value**	**P adjusted**	**Fold change**	**Log$_2$(fold change)**
**SDCBP**	B4DHN5	13.8	(0.7)	[16]	15.3	(0.8)	[14]	<0.001	0.009	2.82	1.50
**IGHV5-51**	A0A0C4DH38	15.5	(0.5)	[62]	16.0	(0.6)	[28]	<0.001	0.052	1.45	0.54
**PCOLCE**	Q15113	16.3	(0.3)	[62]	15.8	(0.7)	[28]	<0.001	0.252	0.70	-0.51
**OGN**	P20774	16.2	(0.4)	[62]	15.3	(1.1)	[28]	<0.001	0.294	0.56	-0.84
**APOD**	C9JF17	17	(0.3)	[62]	16.4	(0.8)	[28]	<0.001	0.295	0.66	-0.60
**MANBA**	O00462	13.3	(0.5)	[53]	13.8	(0.6)	[24]	<0.001	0.329	1.48	0.57
**HBB**	P68871	17.3	(1.6)	[62]	19.1	(2.2)	[28]	<0.001	0.342	3.42	1.77
**MGP**	P08493	15.6	(0.5)	[62]	14.8	(1.0)	[26]	0.001	0.359	0.58	-0.79
**NAGLU**	P54802	13.4	(0.9)	[58]	14.0	(0.6)	[25]	0.001	0.456	1.52	0.60
**MGAT1**	P26572	13.9	(0.4)	[57]	14.2	(0.3)	[18]	0.001	0.467	1.24	0.31
**TNR**	Q92752	13.3	(0.5)	[61]	13.8	(0.5)	[23]	0.001	0.574	1.35	0.43
**FN1**	P02751	16.0	(0.3)	[62]	15.6	(0.6)	[28]	0.001	0.634	0.75	-0.42
**HBA1**	P69905	17.6	(1.5)	[62]	19.2	(2.2)	[28]	0.001	0.784	2.99	1.58
**TPI1**	P60174	14.2	(0.5)	[62]	14.7	(0.8)	[28]	0.001	0.823	1.46	0.55
**SEC23IP**	Q9Y6Y8	14.1	(0.6)	[51]	14.7	(0.7)	[26]	0.001	0.834	1.44	0.53
**C7**	P10643	16.1	(0.4)	[62]	15.6	(0.7)	[28]	0.001	0.874	0.72	-0.47

Name	Uniprot ID	Communicating HC Mean	(SD)	[N]	Obstructive HC Mean	(SD)	[N]	P value	P adjusted	Fold change	Log_2(fold change)
**SERPINF2**	P08697	15.9	(0.3)	[62]	16.1	(0.3)	[28]	0.001	0.89	1.16	0.21
**GLDN**	Q6ZMI3	14.1	(0.7)	[57]	14.8	(0.8)	[25]	0.001	0.905	1.57	0.65
**PROC**	E7END6	13.3	(0.6)	[58]	13.8	(0.6)	[26]	0.001	0.998	1.39	0.48
**A1BG**	P04217-2	16.9	(0.3)	[62]	16.8	(0.3)	[28]	0.274	1	0.95	-0.07
**A2M**	P01023	16.7	(0.3)	[62]	16.9	(0.4)	[28]	0.002	1	1.19	0.25
**ABHD14B**	Q96IU4	12.3	(0.5)	[36]	12.4	(0.8)	[12]	0.835	1	1.04	0.06
**ACSBG2**	Q5FVE4	21.8	(0.8)	[62]	21.8	(1.2)	[28]	0.909	1	1.02	0.03
**ACTA2**	P62736	15.4	(0.8)	[61]	15.8	(0.9)	[28]	0.036	1	1.34	0.42
**ACTB**	P60709	15.4	(0.6)	[62]	15.8	(0.9)	[28]	0.04	1	1.33	0.41
**ACTBL2**	Q562R1	17.2	(0.7)	[62]	17.5	(1.0)	[28]	0.077	1	1.30	0.38
**ACYP2**	P14621	12.0	(0.4)	[56]	12.5	(0.7)	[27]	0.002	1	1.40	0.49
**ADAM22**	F8WAD8	12.8	(1.7)	[62]	12.3	(2.0)	[21]	0.314	1	0.71	-0.49
**ADAM29**	Q9UKF5	15.1	(0.7)	[53]	14.8	(1.0)	[19]	0.406	1	0.86	-0.22
**ADAMTS1**	Q9UHI8	14.5	(0.3)	[48]	14.4	(0.3)	[15]	0.222	1	0.93	-0.10
**ADAMTS4**	O75173	14.6	(0.4)	[55]	14.9	(0.5)	[24]	0.036	1	1.21	0.28
**ADGRB2**	A2A3C1	15.2	(0.6)	[62]	15.2	(1.0)	[26]	0.912	1	0.98	-0.03
**ADGRB3**	O60242	11.4	(0.7)	[55]	11.6	(0.9)	[17]	0.589	1	1.10	0.14
**ADGRL1**	O94910	13.8	(0.6)	[60]	14.2	(1.0)	[26]	0.074	1	1.30	0.38
**ADGRL3**	E7EN28	13.8	(0.9)	[57]	13.8	(0.8)	[24]	0.997	1	1.00	0.00
**ADIPOQ**	Q15848	11.8	(0.4)	[50]	12.0	(0.8)	[19]	0.202	1	1.18	0.24
**AEBP1**	Q8IUX7	13.8	(0.5)	[61]	14.0	(0.6)	[26]	0.341	1	1.09	0.12

		Communicating HC			Obstructive HC						
**Name**	**Uniprot ID**	**Mean**	**(SD)**	**[N]**	**Mean**	**(SD)**	**[N]**	**P value**	**P adjusted**	**Fold change**	**Log_2(fold change)**
**AFM**	P43652	15.1	(0.5)	[62]	15.0	(0.4)	[28]	0.312	1	0.93	-0.10
**AGA**	P20933	13.9	(2.0)	[61]	13.9	(2.1)	[27]	0.900	1	1.04	0.06
**AGRN**	O00468-6	14.2	(0.6)	[62]	14.5	(0.9)	[28]	0.085	1	1.25	0.32
**AGT**	P01019	17.0	(0.4)	[62]	17.1	(0.5)	[28]	0.436	1	1.06	0.08
**AHSG**	P02765	16.7	(0.4)	[62]	16.8	(0.4)	[28]	0.746	1	1.02	0.03
**AK1**	P00568	14.3	(0.9)	[25]	14.4	(0.9)	[13]	0.74	1	1.07	0.10
**ALB**	P02768	19.4	(0.3)	[62]	19.2	(0.3)	[28]	0.042	1	0.90	-0.15
**ALCAM**	Q13740	15.3	(0.3)	[62]	15.4	(0.6)	[28]	0.361	1	1.08	0.11
**ALDOA**	P04075	14.7	(0.4)	[62]	14.8	(0.7)	[28]	0.608	1	1.05	0.07
**ALDOC**	P09972	14.7	(0.4)	[62]	14.6	(0.7)	[28]	0.22	1	0.89	-0.17
**AMBP**	P02760	15.7	(0.5)	[62]	15.7	(0.5)	[28]	0.771	1	1.02	0.03
**ANG**	P03950	13.8	(0.5)	[45]	14.1	(0.7)	[22]	0.104	1	1.22	0.29
**ANXA5**	P08758	14.1	(0.7)	[30]	14.6	(0.7)	[13]	0.043	1	1.42	0.51
**APCS**	P02743	14.3	(0.6)	[62]	14.7	(1.0)	[25]	0.144	1	1.26	0.33
**APLP1**	B7Z4G8	17.0	(0.3)	[62]	16.9	(0.5)	[28]	0.452	1	0.95	-0.07
**APLP2**	Q06481	14.9	(0.6)	[62]	14.9	(0.9)	[28]	0.824	1	1.03	0.04
**APOA1**	P02647	18.2	(0.4)	[62]	18	(0.8)	[28]	0.189	1	0.87	-0.20
**APOA2**	P02652	17.2	(0.5)	[62]	17.1	(0.7)	[28]	0.376	1	0.91	-0.14
**APOA4**	P06727	16.6	(0.5)	[62]	16.3	(0.5)	[28]	0.012	1	0.81	-0.30
**APOB**	P04114	13.7	(1.0)	[61]	14.6	(1.4)	[24]	0.01	1	1.82	0.86
**APOC1**	K7ERI9	15.4	(0.6)	[62]	15.6	(0.8)	[27]	0.333	1	1.13	0.18

Name	Uniprot ID	Communicating HC Mean	(SD)	[N]	Obstructive HC Mean	(SD)	[N]	P value	P adjusted	Fold change	Log_2(fold change)
**APOC3**	B0YIW2	16.2	(0.7)	[62]	15.9	(1.4)	[28]	0.345	1	0.83	-0.27
**APOC4-APOC2**	A0A024R0T9	14.4	(0.9)	[53]	14.7	(1.4)	[18]	0.364	1	1.25	0.32
**APOE**	P02649	17.7	(0.5)	[62]	17.2	(0.8)	[28]	0.003	1	0.69	-0.54
**APOH**	P02749	15.6	(0.4)	[62]	15.8	(0.5)	[28]	0.039	1	1.16	0.21
**APOL1**	O14791	12.9	(0.8)	[52]	13.4	(1.1)	[18]	0.128	1	1.37	0.45
**APOM**	O95445	15.3	(0.5)	[62]	15.6	(0.8)	[25]	0.127	1	1.21	0.28
**APP**	P05067	15.2	(0.4)	[62]	15.4	(0.5)	[28]	0.056	1	1.17	0.23
**ARPC4-TTLL3**	A0A0A6YYG9	16.5	(0.4)	[62]	16.4	(0.3)	[28]	0.445	1	0.96	-0.06
**ART3**	E7ESB3	15.1	(0.4)	[62]	15.2	(0.7)	[28]	0.256	1	1.12	0.16
**ASAH1**	A0A1B0GTM3	14.8	(0.6)	[58]	15.2	(0.7)	[24]	0.016	1	1.31	0.39
**ATP1A1**	P05023	13.8	(0.9)	[24]	13.9	(0.5)	[14]	0.742	1	1.05	0.07
**ATP1B1**	P05026	13.1	(1.0)	[53]	13.6	(0.9)	[20]	0.045	1	1.42	0.51
**ATP6AP1**	Q15904	15.1	(0.4)	[62]	14.7	(0.7)	[28]	0.012	1	0.77	-0.38
**ATRN**	O75882	13.9	(0.3)	[56]	14.1	(0.5)	[26]	0.103	1	1.13	0.18
**AXL**	P30530	15.1	(1.0)	[45]	15.6	(0.5)	[24]	0.01	1	1.39	0.48
**AZGP1**	P25311	16.8	(0.3)	[62]	16.5	(0.4)	[28]	0.008	1	0.85	-0.23
**B2M**	P61769	17.1	(0.4)	[62]	17.2	(0.6)	[28]	0.795	1	1.02	0.03
**B3GALNT1**	O75752	13.4	(0.6)	[58]	13.5	(0.6)	[17]	0.5	1	1.09	0.12
**B3GNT2**	Q9NY97	12.5	(0.9)	[49]	12.3	(1.1)	[11]	0.606	1	0.88	-0.18
**B4GALT1**	P15291	13.8	(0.5)	[53]	14.2	(0.6)	[15]	0.072	1	1.23	0.30
**B4GAT1**	O43505	17.0	0.7)	[62]	16.9	(0.9)	[28]	0.492	1	0.91	-0.14

Name	Uniprot ID	Communicating HC Mean	(SD)	[N]	Obstructive HC Mean	(SD)	[N]	P value	P adjusted	Fold change	Log$_2$(fold change)
**BASP1**	P80723	11.9	(0.7)	[61]	12.3	(0.9)	[28]	0.079	1	1.28	0.36
**BCAN**	Q96GW7	16.1	(0.6)	[62]	16.3	(0.8)	[28]	0.133	1	1.20	0.26
**BCHE**	P06276	12.5	(1.2)	[26]	12.9	(1.2)	[11]	0.305	1	1.37	0.45
**BGN**	P21810	13.5	(1.0)	[62]	13.6	(1.2)	[27]	0.759	1	1.06	0.08
**BLVRB**	P30043	12.7	(1.1)	[23]	14.0	(1.4)	[17]	0.004	1	2.46	1.30
**BTD**	P43251	16.3	(0.3)	[62]	16.5	(0.5)	[28]	0.191	1	1.10	0.14
**C16orf89**	A0A0A0MT71	14.6	(0.4)	[62]	14.4	(0.7)	[24]	0.254	1	0.88	-0.18
**C1QA**	P02745	14.5	(0.5)	[62]	14.4	(0.7)	[28]	0.621	1	0.95	-0.07
**C1QB**	D6R934	15.4	(0.4)	[62]	15.4	(0.4)	[28]	0.693	1	1.03	0.04
**C1QC**	P02747	16.8	(0.4)	[62]	16.9	(0.3)	[28]	0.387	1	1.04	0.06
**C1QTNF3-AMACR**	E9PGA6	12.7	(1.5)	[42]	12.9	(0.7)	[10]	0.471	1	1.18	0.24
**C1QTNF5**	Q9BXJ0	13.2	(0.7)	[59]	13.6	(1.0)	[25]	0.071	1	1.33	0.41
**C1R**	B4DPQ0	15.6	(0.3)	[62]	15.4	(0.4)	[28]	0.019	1	0.85	-0.23
**C1RL**	Q9NZP8	13.7	(0.5)	[61]	13.8	(0.5)	[21]	0.64	1	1.04	0.06
**C1S**	P09871	16.3	(0.3)	[62]	15.9	(0.6)	[28]	0.012	1	0.81	-0.30
**C2**	P06681	14.6	(0.3)	[62]	14.6	(0.3)	[28]	0.514	1	0.96	-0.06
**C2orf40**	B8ZZE5	16.1	(0.9)	[62]	16.7	(1.7)	[28]	0.083	1	1.51	0.59
**C3**	P01024	17.0	(0.2)	[62]	17.1	(0.2)	[28]	0.078	1	1.06	0.08
**C4A**	A0A0G2JPR0	15.5	(0.7)	[62]	15.6	(0.7)	[28]	0.388	1	1.10	0.14
**C4B**	P0C0L5	16.8	(0.3)	[62]	16.8	(0.4)	[28]	0.698	1	1.03	0.04
**C4BPA**	P04003	12.5	(1.3)	[37]	12.8	(1.4)	[15]	0.47	1	1.23	0.30

Name	Uniprot ID	Communicating HC Mean	(SD)	[N]	Obstructive HC Mean	(SD)	[N]	P value	P adjusted	Fold change	Log$_2$(fold change)
**C5**	P01031	13.9	(0.4)	[62]	14.0	(0.5)	[28]	0.212	1	1.10	0.14
**C6**	P13671	14.8	(0.4)	[62]	15.0	(0.4)	[28]	0.084	1	1.12	0.16
**C8A**	P07357	15.3	(0.4)	[62]	15.4	(0.6)	[28]	0.14	1	1.13	0.18
**C8B**	F5GY80	14.0	(0.3)	[62]	14.1	(0.5)	[28]	0.232	1	1.09	0.12
**C8G**	P07360	14.2	(0.8)	[56]	14.5	(0.7)	[27]	0.177	1	1.19	0.25
**C9**	P02748	15.2	(0.4)	[62]	15.1	(0.4)	[28]	0.197	1	0.92	-0.12
**CA1**	P00915	14.6	(1.3)	[45]	15.6	(1.7)	[25]	0.019	1	1.92	0.94
**CA2**	P00918	13.4	(0.7)	[39]	14.2	(1.1)	[22]	0.006	1	1.73	0.79
**CACHD1**	A0A0A0MQY7	13.3	(0.5)	[61]	13.4	(0.6)	[27]	0.721	1	1.04	0.06
**CACNA2D1**	P54289	14.8	(0.3)	[62]	14.6	(0.6)	[28]	0.051	1	0.83	-0.27
**CADM1**	A0A087X0T8	16.1	(0.5)	[61]	15.7	(1.0)	[28]	0.088	1	0.79	-0.34
**CADM2**	Q8N3J6	14.8	(0.5)	[62]	14.6	(0.5)	[28]	0.139	1	0.89	-0.17
**CADM3**	Q8N126	15.8	(0.4)	[62]	15.4	(0.5)	[28]	0.007	1	0.80	-0.32
**CADM4**	Q8NFZ8	16.4	(0.4)	[62]	16.5	(0.6)	[28]	0.204	1	1.12	0.16
**CALR**	P27797	14.0	(0.3)	[62]	14.2	(1.0)	[28]	0.245	1	1.17	0.23
**CALY**	Q9NYX4	13.6	(0.8)	[53]	14.0	(0.7)	[21]	0.063	1	1.29	0.37
**CAMK2A**	Q9UQM7	14.2	(0.7)	[45]	14.4	(0.9)	[14]	0.426	1	1.15	0.20
**CAMK2B**	Q13554	12.7	(0.7)	[52]	12.9	(1.3)	[13]	0.61	1	1.14	0.19
**CANT1**	Q8WVQ1	13.3	(0.5)	[48]	13.6	(0.5)	[22]	0.012	1	1.26	0.33
**CARTPT**	Q16568	13.7	(0.6)	[61]	13.8	(0.6)	[27]	0.246	1	1.12	0.16
**CASP14**	P31944	14.3	(0.9)	[34]	14.2	(1.1)	[15]	0.679	1	0.91	-0.14

Name	Uniprot ID	Communicating HC			Obstructive HC			P value	P adjusted	Fold change	Log_2(fold change)
		Mean	(SD)	[N]	Mean	(SD)	[N]				
**CAT**	P04040	14.8	(1.5)	[29]	14.0	(1.0)	[14]	0.045	1	0.57	-0.81
**CBLN1**	P23435	12.6	(0.8)	[47]	12.9	(1.3)	[11]	0.514	1	1.20	0.26
**CBR1**	P16152	14.2	(0.7)	[60]	14.2	(0.8)	[28]	0.982	1	1.00	0.00
**CCDC93**	F8W9X7	14.4	(0.5)	[55]	14.5	(0.6)	[20]	0.894	1	1.01	0.01
**CCHCR1**	A0A0G2JHN4	13.5	(2.1)	[45]	14.0	(2.3)	[19]	0.339	1	1.51	0.59
**CCL14**	A0A087X089	13.6	(0.5)	[32]	13.6	(0.6)	[12]	0.785	1	0.96	-0.06
**CCP110**	O43303	21.6	(0.6)	[41]	21.5	(0.8)	[19]	0.519	1	0.91	-0.14
**CD109**	Q6YHK3	12.2	(1.0)	[41]	12.9	(0.9)	[15]	0.023	1	1.61	0.69
**CD14**	P08571	16.4	(0.4)	[62]	16.2	(0.5)	[28]	0.036	1	0.85	-0.23
**CD163**	C9JHR8	13.6	(0.7)	[45]	13.6	(0.5)	[13]	0.87	1	0.98	-0.03
**CD44**	H0YD13	17.1	(0.5)	[62]	17.3	(0.8)	[28]	0.238	1	1.15	0.20
**CD59**	E9PNW4	15.2	(0.9)	[62]	15.8	(0.9)	[28]	0.002	1	1.55	0.63
**CD9**	A6NNI4	15.2	(0.5)	[26]	15.9	(1.0)	[16]	0.027	1	1.57	0.65
**CD99**	P14209	16.4	(0.8)	[62]	16.2	(0.9)	[28]	0.355	1	0.88	-0.18
**CD99L2**	Q8TCZ2	16.9	(0.5)	[62]	16.9	(0.5)	[28]	0.851	1	1.02	0.03
**CDH13**	P55290	15.5	(0.4)	[62]	15.5	(0.6)	[28]	0.686	1	0.96	-0.06
**CDH2**	P19022	16.3	(0.3)	[62]	16.4	(0.5)	[28]	0.392	1	1.06	0.08
**CDH4**	P55283	13.3	(0.7)	[62]	13.8	(1.0)	[27]	0.042	1	1.36	0.44
**CDH6**	D6RF86	13.2	(0.5)	[61]	13.1	(0.9)	[19]	0.641	1	0.93	-0.10
**CELSR2**	Q9HCU4	14.0	(0.5)	[41]	13.9	(0.7)	[16]	0.583	1	0.93	-0.10
**CETP**	P11597	15.3	(0.4)	[46]	15.9	(0.8)	[13]	0.015	1	1.60	0.68

		Communicating HC			Obstructive HC						
**Name**	**Uniprot ID**	**Mean**	**(SD)**	**[N]**	**Mean**	**(SD)**	**[N]**	**P value**	**P adjusted**	**Fold change**	**Log$_2$(fold change)**
**CFD**	K7ERG9	16.4	(0.4)	[62]	16.1	(0.5)	[28]	0.014	1	0.81	-0.30
**CFH**	P08603	15.5	(0.3)	[62]	15.4	(0.3)	[28]	0.463	1	0.96	-0.06
**CFHR1**	B1AKG0	14.8	(0.7)	[62]	15.4	(0.9)	[28]	0.014	1	1.42	0.51
**CFI**	E7ETH0	14.6	(0.3)	[62]	14.7	(0.3)	[28]	0.114	1	1.07	0.10
**CFL1**	E9PK25	13.6	(0.6)	[61]	13.9	(0.9)	[24]	0.13	1	1.25	0.32
**CGREF1**	Q99674	13.9	(0.5)	[62]	13.8	(1.0)	[27]	0.461	1	0.90	-0.15
**CHGA**	P10645	16.6	(0.4)	[62]	16.6	(0.6)	[28]	0.967	1	1.00	0.00
**CHGB**	P05060	16.3	(0.5)	[62]	16.1	(0.6)	[28]	0.111	1	0.87	-0.20
**CHI3L1**	P36222	16.4	(0.5)	[62]	16.4	(0.7)	[28]	0.947	1	0.99	-0.01
**CHI3L2**	Q15782	13.2	(0.7)	[60]	13.4	(1.0)	[25]	0.322	1	1.16	0.21
**CHL1**	O00533	15.7	(0.4)	[62]	15.6	(0.6)	[28]	0.803	1	0.98	-0.03
**CHST10**	O43529	18.0	(2.2)	[47]	18.9	(2.3)	[19]	0.135	1	1.91	0.93
**CKB**	P12277	15.4	(1.1)	[60]	15.4	(1.4)	[26]	0.941	1	1.02	0.03
**CKM**	P06732	14.7	(1.1)	[52]	15.6	(2.0)	[11]	0.207	1	1.79	0.84
**CLCNKB**	A0A087X136	16.5	(0.6)	[62]	16.4	(0.8)	[28]	0.437	1	0.91	-0.14
**CLEC11A**	Q9Y240	13.1	(0.7)	[51]	13.1	(0.7)	[16]	0.742	1	1.05	0.07
**CLEC3B**	E9PHK0	16.9	(0.3)	[62]	16.9	(0.4)	[28]	0.778	1	0.98	-0.03
**CLN5**	A0A024R644	13.9	(0.6)	[53]	14.4	(0.8)	[26]	0.003	1	1.48	0.57
**CLSTN1**	O94985	16.7	(0.4)	[62]	16.7	(0.5)	[28]	0.891	1	1.01	0.01
**CLSTN2**	Q9H4D0	11.7	(1.2)	[14]	12.1	(1.1)	[12]	0.311	1	1.37	0.45
**CLSTN3**	Q9BQT9	12.8	(1.7)	[62]	13.1	(1.9)	[28]	0.616	1	1.16	0.21

Name	Uniprot ID	Communicating HC Mean	(SD)	[N]	Obstructive HC Mean	(SD)	[N]	P value	P adjusted	Fold change	Log_2(fold change)
**CLU**	P10909	17.3	(0.3)	[62]	17.2	(0.4)	[28]	0.163	1	0.92	-0.12
**CNDP1**	Q96KN2	17.2	(0.4)	[62]	17.3	(0.5)	[28]	0.564	1	1.05	0.07
**CNTFR**	P26992	13.0	(0.8)	[59]	13.0	(1.2)	[17]	0.923	1	0.98	-0.03
**CNTN1**	Q12860	16.1	(0.4)	[62]	16.0	(0.5)	[28]	0.715	1	0.97	-0.04
**CNTN2**	A0A1W2PQ11	16.2	(0.5)	[62]	16.5	(0.9)	[28]	0.087	1	1.25	0.32
**CNTNAP4**	A0A087WTA1	14.2	(0.7)	[61]	14.1	(0.9)	[27]	0.611	1	0.93	-0.10
**COCH**	O43405	13.9	(0.6)	[46]	14.1	(0.5)	[18]	0.153	1	1.16	0.21
**COL15A1**	A0A087X0K0	13.8	(0.5)	[49]	13.9	(0.6)	[16]	0.764	1	1.03	0.04
**COL18A1**	P39060	15.3	(0.3)	[62]	15.0	(0.7)	[28]	0.070	1	0.84	-0.25
**COL1A1**	P02452	14.3	(0.5)	[62]	14.2	(0.8)	[25]	0.673	1	0.95	-0.07
**COL1A2**	A0A087WTA8	14.2	(0.3)	[62]	14.4	(0.9)	[28]	0.288	1	1.14	0.19
**COL3A1**	P02461	13.2	(0.8)	[45]	13.4	(0.4)	[12]	0.310	1	1.13	0.18
**COL6A1**	A0A087X0S5	15.0	(0.4)	[62]	15.2	(0.5)	[28]	0.109	1	1.12	0.16
**COL6A3**	P12111	13.7	(0.4)	[62]	13.6	(0.5)	[19]	0.637	1	0.96	-0.06
**COLEC12**	Q5KU26	14.5	(0.5)	[60]	14.6	(0.6)	[27]	0.264	1	1.11	0.15
**COTL1**	Q14019	12.6	(0.7)	[38]	12.7	(0.8)	[14]	0.649	1	1.08	0.11
**CP**	P00450	16.2	(0.3)	[62]	16.4	(0.4)	[28]	0.014	1	1.18	0.24
**CPB2**	A0A087WSY5	15.1	(0.4)	[62]	15.4	(0.4)	[28]	0.003	1	1.20	0.26
**CPE**	P16870	17.0	(0.5)	[62]	17.3	(0.6)	[28]	0.044	1	1.21	0.28
**CPN2**	P22792	14.0	(0.6)	[57]	14.6	(0.8)	[21]	0.002	1	1.55	0.63
**CPQ**	Q9Y646	15.7	(0.6)	[62]	15.9	(0.6)	[28]	0.357	1	1.09	0.12

Name	Uniprot ID	Communicating HC Mean	(SD)	[N]	Obstructive HC Mean	(SD)	[N]	P value	P adjusted	Fold change	Log_2(fold change)
**CPVL**	Q9H3G5	14.8	(0.6)	[62]	15.2	(0.8)	[27]	0.006	1	1.41	0.50
**CPXM2**	Q8N436	13.0	(0.7)	[23]	13.6	(0.7)	[14]	0.016	1	1.54	0.62
**CRISPLD1**	E5RJS4	14.8	(0.6)	[15]	15.4	(0.4)	[15]	0.003	1	1.56	0.64
**CRP**	P02741	14.0	(1.0)	[37]	14.4	(1.0)	[20]	0.221	1	1.28	0.36
**CRTAC1**	A0A0C4DFP6	15.6	(0.3)	[62]	15.5	(0.6)	[28]	0.603	1	0.96	-0.06
**CRYAB**	A0A024R3B9	13.4	(0.9)	[19]	13.9	(1.0)	[10]	0.147	1	1.49	0.58
**CSF1**	P09603	14.6	(0.4)	[62]	14.9	(0.6)	[27]	0.017	1	1.23	0.30
**CSF1R**	E9PEK4	15.5	(0.4)	[62]	15.7	(0.6)	[28]	0.092	1	1.15	0.20
**CSPG5**	A0A087WUT8	14.7	(0.4)	[54]	15.0	(0.6)	[18]	0.099	1	1.21	0.28
**CST3**	P01034	18.1	(0.3)	[62]	18.0	(0.5)	[28]	0.448	1	0.94	-0.09
**CSTB**	P04080	13.9	(0.6)	[49]	14.4	(0.7)	[22]	0.014	1	1.38	0.46
**CTBS**	Q01459	14.8	(0.4)	[62]	15.2	(0.7)	[27]	0.020	1	1.28	0.36
**CTSA**	P10619	13.4	(0.5)	[53]	13.6	(0.8)	[27]	0.306	1	1.13	0.18
**CTSB**	P07858	13.6	(0.6)	[59]	14.0	(0.7)	[27]	0.024	1	1.29	0.37
**CTSC**	P53634	13.2	(0.5)	[37]	13.8	(0.9)	[19]	0.014	1	1.51	0.59
**CTSD**	A0A1B0GV23	16.9	(0.6)	[62]	17.2	(1.1)	[28]	0.127	1	1.28	0.36
**CTSF**	Q9UBX1	14.8	(0.3)	[60]	15.0	(0.3)	[26]	0.008	1	1.16	0.21
**CTSH**	A0A087X0D5	15.4	(0.5)	[60]	15.6	(1.0)	[28]	0.337	1	1.15	0.20
**CTSL**	P07711	15.2	(0.5)	[62]	15.7	(0.8)	[28]	0.014	1	1.35	0.43
**CTSS**	P25774	14.1	(0.5)	[57]	14.2	(0.4)	[23]	0.078	1	1.14	0.19
**CTSZ**	Q9UBR2	15.5	(0.6)	[62]	15.9	(1.0)	[27]	0.029	1	1.37	0.45

Name	Uniprot ID	Communicating HC Mean	(SD)	[N]	Obstructive HC Mean	(SD)	[N]	P value	P adjusted	Fold change	Log_2(fold change)
**CUTA**	O60888	15.2	(0.8)	[61]	15.3	(1.2)	[28]	0.851	1	1.03	0.04
**CXCL16**	Q9H2A7	14.6	(0.3)	[33]	14.1	(0.7)	[16]	0.023	1	0.74	-0.43
**CYCS**	C9JFR7	13.3	(0.5)	[58]	13.2	(0.7)	[20]	0.511	1	0.92	-0.12
**DAG1**	Q14118	15.8	(0.3)	[62]	15.7	(0.5)	[28]	0.467	1	0.95	-0.07
**DBI**	A0A0A0MTI5	15.2	(0.5)	[62]	15.5	(0.6)	[28]	0.075	1	1.19	0.25
**DCC**	E7EQM8	13.5	(0.6)	[45]	13.7	(1.2)	[14]	0.607	1	1.13	0.18
**DCD**	P81605	14.1	(1.9)	[40]	14.4	(1.4)	[22]	0.485	1	1.23	0.30
**DCN**	P07585	14.0	(0.8)	[62]	14.3	(0.8)	[22]	0.134	1	1.22	0.29
**DDAH1**	O94760	12.6	(1.6)	[44]	13.8	(1.3)	[18]	0.003	1	2.29	1.20
**DDR1**	A0A0A0MSX3	13.9	(0.6)	[51]	14.3	(0.8)	[23]	0.038	1	1.33	0.41
**DKK3**	F6SYF8	16.5	(0.4)	[62]	16.4	(0.6)	[28]	0.604	1	0.95	-0.07
**DNER**	Q8NFT8	12.1	(0.5)	[55]	12.1	(0.5)	[13]	0.669	1	0.95	-0.07
**DPP7**	Q9UHL4	14.1	(0.6)	[61]	14.3	(0.8)	[28]	0.271	1	1.15	0.20
**DPYSL2**	A0A1C7CYX9	14.3	(1.0)	[55]	14.6	(1.1)	[22]	0.282	1	1.22	0.29
**DSC2**	Q02487	14.3	(0.4)	[62]	14.4	(0.6)	[24]	0.415	1	1.08	0.11
**ECM1**	Q16610	14.9	(0.4)	[62]	14.8	(0.5)	[28]	0.429	1	0.94	-0.09
**ECM2**	O94769	14.7	(0.5)	[62]	14.2	(0.8)	[25]	0.012	1	0.71	-0.49
**EEF1A1**	P68104	13.6	(1.1)	[21]	14.7	(1.5)	[18]	0.013	1	2.18	1.12
**EFCAB14**	O75071	14.0	(0.6)	[56]	14.2	(0.8)	[17]	0.300	1	1.17	0.23
**EFEMP1**	A0A0U1RQV3	16.8	(0.4)	[62]	17.2	(0.7)	[28]	0.024	1	1.27	0.34
**EFNA1**	P20827	13.5	(1.2)	[56]	13.7	(1.3)	[24]	0.582	1	1.12	0.16

		Communicating HC			Obstructive HC						
**Name**	**Uniprot ID**	**Mean**	**(SD)**	**[N]**	**Mean**	**(SD)**	**[N]**	**P value**	**P adjusted**	**Fold change**	**Log_2(fold change)**
**EIF5A**	I3L397	12.6	(0.8)	[37]	13.1	(1.0)	[11]	0.198	1	1.37	0.45
**ENDOD1**	O94919	15.7	(0.3)	[62]	15.8	(0.7)	[28]	0.235	1	1.12	0.16
**ENO1**	P06733	15.1	(0.7)	[61]	15.4	(0.9)	[27]	0.092	1	1.27	0.34
**ENO2**	P09104	14.6	(0.7)	[61]	14.6	(1.1)	[28]	0.949	1	1.01	0.01
**ENPP2**	E7EUF1	17.4	(0.6)	[62]	17.8	(1.2)	[28]	0.086	1	1.33	0.41
**ENPP4**	Q9Y6X5	13.0	(0.5)	[60]	13.4	(0.9)	[26]	0.069	1	1.28	0.36
**ENPP6**	Q6UWR7	13.5	(0.4)	[29]	14.0	(0.7)	[14]	0.027	1	1.39	0.48
**EPDR1**	Q9UM22	13.8	(0.6)	[58]	14.0	(0.7)	[25]	0.130	1	1.19	0.25
**EPHA4**	E9PG71	15.4	(0.8)	[62]	15.3	(0.8)	[28]	0.407	1	0.90	-0.15
**ERAP1**	Q9NZ08	12.3	(0.9)	[27]	13.0	(0.7)	[12]	0.010	1	1.70	0.77
**ERN1**	O75460	15.4	(1.2)	[61]	15.5	(1.3)	[26]	0.623	1	1.11	0.15
**ESD**	H7BZT7	12.7	(0.7)	[25]	13.4	(0.7)	[11]	0.010	1	1.63	0.70
**EXTL2**	Q9UBQ6	15.0	(0.4)	[62]	14.8	(0.7)	[24]	0.091	1	0.83	-0.27
**F10**	P00742	14.3	(0.5)	[43]	14.5	(0.5)	[17]	0.368	1	1.10	0.14
**F12**	P00748	15.7	(0.5)	[62]	15.8	(0.5)	[28]	0.733	1	1.03	0.04
**F2**	P00734	15.9	(0.3)	[62]	16.0	(0.3)	[28]	0.145	1	1.08	0.11
**F5**	A0A0A0MRJ7	15.5	(0.5)	[62]	15.8	(1.0)	[28]	0.140	1	1.23	0.30
**F9**	P00740	13.1	(0.6)	[62]	13.4	(0.7)	[28]	0.063	1	1.23	0.30
**FAM3C**	Q92520	15.5	(0.3)	[62]	15.7	(0.6)	[28]	0.353	1	1.08	0.11
**FAT2**	Q9NYQ8	12.9	(0.7)	[52]	12.9	(0.9)	[16]	0.977	1	1.00	0.00
**FBLN1**	P23142	16.6	(0.4)	[62]	16.8	(0.7)	[28]	0.331	1	1.10	0.14

Name	Uniprot ID	Communicating HC Mean	(SD)	[N]	Obstructive HC Mean	(SD)	[N]	P value	P adjusted	Fold change	Log_2(fold change)
**FBLN5**	G3V4U0	15.1	(0.8)	[62]	14.5	(0.9)	[27]	0.009	1	0.67	-0.58
**FBLN7**	Q53RD9	12.6	(0.4)	[56]	12.7	(0.8)	[14]	0.745	1	1.05	0.07
**FCGBP**	Q9Y6R7	14.3	(0.7)	[62]	14.7	(0.7)	[26]	0.058	1	1.26	0.33
**FCGR2A**	P12318	14.3	(0.3)	[39]	14.1	(0.3)	[11]	0.127	1	0.90	-0.15
**FCGR3A**	A0A1W2PQB1	14.5	(0.8)	[62]	14.4	(0.9)	[28]	0.583	1	0.93	-0.10
**FETUB**	Q9UGM5	14	(0.7)	[62]	14.2	(0.8)	[25]	0.309	1	1.14	0.19
**FGA**	P02671	14.2	(0.7)	[62]	14.3	(0.6)	[28]	0.509	1	1.07	0.10
**FGB**	P02675	15.4	(0.9)	[62]	15.6	(0.7)	[28]	0.176	1	1.19	0.25
**FGFR1**	P11362	15.5	(0.6)	[38]	16.1	(1.6)	[14]	0.197	1	1.52	0.60
**FGFR2**	A0A0A0MR25	15.7	(0.6)	[62]	15.8	(0.7)	[27]	0.412	1	1.10	0.14
**FGG**	P02679	14.8	(0.8)	[62]	14.9	(0.7)	[28]	0.445	1	1.10	0.14
**FKBP1A**	P62942	14.2	(0.6)	[15]	14.6	(0.3)	[14]	0.026	1	1.35	0.43
**FMOD**	Q06828	13.8	(0.5)	[61]	13.8	(0.7)	[19]	0.973	1	1.00	0.00
**FRRS1L**	Q9P0K9	10.2	(2.0)	[50]	10.8	(1.8)	[12]	0.324	1	1.50	0.58
**FRZB**	Q92765	14.4	(0.8)	[60]	14.3	(1.0)	[26]	0.825	1	0.97	-0.04
**FSTL1**	Q12841	13.7	(0.6)	[62]	14	(1.0)	[28]	0.192	1	1.20	0.26
**FSTL4**	Q6MZW2	13.1	(0.7)	[58]	13.2	(0.8)	[15]	0.758	1	1.05	0.07
**FTH1**	P02794	11.7	(0.9)	[33]	12.4	(1.2)	[18]	0.058	1	1.54	0.62
**FTL**	P02792	13.1	(0.7)	[55]	13.5	(0.7)	[24]	0.057	1	1.25	0.32
**FUCA1**	P04066	14.6	(0.7)	[62]	14.6	(0.8)	[28]	0.691	1	1.05	0.07
**FUCA2**	Q9BTY2	14.7	(0.8)	[56]	15	(0.7)	[27]	0.057	1	1.26	0.33

Name	Uniprot ID	Communicating HC Mean	(SD)	[N]	Obstructive HC Mean	(SD)	[N]	P value	P adjusted	Fold change	Log2(fold change)
**FXYD6**	Q9H0Q3	15.0	(0.6)	[61]	15.3	(1.0)	[27]	0.193	1	1.20	0.26
**GALNT2**	Q10471	13.9	(0.3)	[59]	14	(0.7)	[26]	0.456	1	1.08	0.11
**GALNT7**	Q86SF2	13.7	(0.4)	[41]	13.8	(0.4)	[17]	0.538	1	1.06	0.08
**GANAB**	Q14697	12.7	(0.6)	[59]	12.5	(0.5)	[24]	0.303	1	0.91	-0.14
**GAP43**	P17677	11.4	(0.8)	[50]	11.5	(1.2)	[20]	0.717	1	1.08	0.11
**GAPDH**	P04406	14.7	(0.7)	[62]	14.8	(1.1)	[28]	0.631	1	1.08	0.11
**GC**	P02774	16.6	(0.3)	[62]	16.6	(0.4)	[28]	0.89	1	0.99	-0.01
**GDA**	Q9Y2T3	13.7	(0.7)	[60]	13.6	(0.9)	[27]	0.567	1	0.93	-0.10
**GDI1**	P31150	14.5	(0.8)	[40]	14.7	(0.8)	[19]	0.455	1	1.12	0.16
**GDI2**	P50395	13.5	(0.6)	[61]	13.7	(1.1)	[28]	0.348	1	1.15	0.20
**GFAP**	A0A1W2PR46	14.7	(1.5)	[19]	14.8	(0.7)	[18]	0.681	1	1.12	0.16
**GGH**	Q92820	14.6	(0.5)	[61]	14.9	(0.6)	[28]	0.049	1	1.21	0.28
**GLOD4**	F6TLX2	15.4	(1.4)	[38]	15.5	(1.5)	[14]	0.818	1	1.08	0.11
**GM2A**	P17900	16.4	(0.4)	[62]	16.5	(0.8)	[28]	0.447	1	1.09	0.12
**GNPTG**	Q9UJJ9	15.5	(0.4)	[62]	15.8	(0.5)	[27]	0.011	1	1.21	0.28
**GNS**	F6S8M0	14.2	(0.6)	[35]	14.6	(0.7)	[19]	0.047	1	1.31	0.39
**GOLIM4**	F8W785	10.3	(1.3)	[40]	10.6	(1.4)	[17]	0.507	1	1.20	0.26
**GOLM1**	Q8NBJ4	13.5	(0.6)	[61]	13.6	(0.7)	[27]	0.586	1	1.06	0.08
**GOT1**	P17174	14.9	(0.3)	[62]	15.0	(0.7)	[28]	0.694	1	1.04	0.06
**GPC1**	P35052	13.2	(1.6)	[45]	13.3	(1.7)	[22]	0.747	1	1.10	0.14
**GPI**	A0A0A0MTS2	12.5	(0.6)	[37]	13.1	(0.8)	[15]	0.013	1	1.54	0.62

		Communicating HC			Obstructive HC						
**Name**	**Uniprot ID**	**Mean**	**(SD)**	**[N]**	**Mean**	**(SD)**	**[N]**	**P value**	**P adjusted**	**Fold change**	**Log_2(fold change)**
**GPLD1**	P80108	13.5	(0.6)	[38]	14.0	(0.7)	[14]	0.048	1	1.37	0.45
**GPR37**	O15354	16.0	(0.3)	[62]	16.3	(0.6)	[28]	0.009	1	1.28	0.36
**GPR37L1**	O60883	14.6	(0.6)	[62]	14.7	(1.1)	[28]	0.433	1	1.13	0.18
**GPX3**	A0A087X1J7	15.9	(0.4)	[62]	16.2	(1.0)	[28]	0.107	1	1.24	0.31
**GRIA4**	G3V164	12.8	(0.5)	[57]	12.9	(0.6)	[17]	0.794	1	1.03	0.04
**GSN**	P06396	16.9	(0.2)	[62]	16.7	(0.3)	[28]	0.004	1	0.87	-0.20
**GSS**	P48637	13.6	(0.9)	[20]	13.8	(1.3)	[10]	0.573	1	1.21	0.28
**GSTO1**	P78417	13.8	(0.6)	[62]	14.2	(0.8)	[28]	0.041	1	1.29	0.37
**GSTP1**	P09211	15.1	(0.6)	[62]	15.3	(0.8)	[28]	0.110	1	1.21	0.28
**GAA**	P10253	13.0	(1.2)	[41]	13.7	(0.7)	[18]	0.008	1	1.62	0.70
**HARS**	B3KWE1	21.3	(0.4)	[24]	21.3	(1.1)	[17]	0.994	1	1.00	0.00
**HBD**	P02042	15.7	(0.8)	[51]	16.6	(1.9)	[26]	0.022	1	1.91	0.93
**HBG2**	P69892	13.9	(1.9)	[31]	15.0	(2.0)	[18]	0.087	1	2.04	1.03
**hCG_2039566**	A0A0U1RR32	13.5	(1.7)	[12]	15.4	(1.5)	[11]	0.01	1	3.74	1.90
**HEXA**	H3BP20	14.6	(0.6)	[62]	14.8	(0.8)	[28]	0.213	1	1.15	0.20
**HEXB**	P07686	14.7	(0.5)	[62]	15.1	(0.7)	[28]	0.021	1	1.29	0.37
**HGFAC**	D6RAR4	14.0	(0.5)	[48]	14.1	(0.5)	[19]	0.594	1	1.05	0.07
**HIST1H2BK**	O60814	14.3	(1.0)	[40]	15.1	(1.0)	[22]	0.003	1	1.75	0.81
**HIST1H4A**	P62805	13.5	(1.1)	[60]	14.2	(1.3)	[28]	0.013	1	1.67	0.74
**HLA-C**	A0A140T921	13.7	(0.9)	[21]	13.6	(0.9)	[10]	0.829	1	0.95	-0.07
**HP**	P00738	17.0	(1.0)	[62]	17.1	(1.1)	[28]	0.602	1	1.09	0.12

Name	Uniprot ID	Communicating HC Mean	(SD)	[N]	Obstructive HC Mean	(SD)	[N]	P value	P adjusted	Fold change	Log_2(fold change)
**HPR**	P00739	14.5	(0.8)	[62]	15.0	(1.2)	[28]	0.044	1	1.43	0.52
**HPRT1**	P00492	13.2	(1.1)	[25]	13.5	(0.9)	[11]	0.374	1	1.24	0.31
**HPX**	P02790	18.1	(0.3)	[62]	18.4	(0.5)	[28]	0.022	1	1.20	0.26
**HRG**	P04196	15.7	(0.4)	[62]	15.5	(0.5)	[28]	0.092	1	0.89	-0.17
**HS6ST3**	Q8IZP7	14.2	(1.2)	[47]	14.5	(0.6)	[11]	0.205	1	1.24	0.31
**HSP90B1**	P14625	13.8	(0.4)	[49]	14.0	(0.7)	[20]	0.334	1	1.12	0.16
**HSP90AA1**	P07900	13.8	(0.7)	[24]	14.7	(0.8)	[12]	0.003	1	1.87	0.90
**HSPA13**	P48723	13.1	(0.7)	[40]	13.2	(0.8)	[23]	0.514	1	1.10	0.14
**HSPA1B**	A0A0G2JIW1	13.9	(0.9)	[41]	14.5	(1.2)	[15]	0.078	1	1.53	0.61
**HSPA5**	P11021	14.2	(0.3)	[62]	14.3	(0.4)	[27]	0.425	1	1.05	0.07
**HSPA8**	P11142	14.1	(0.8)	[61]	14.5	(1.2)	[27]	0.091	1	1.36	0.44
**HSPG2**	P98160	14.3	(0.3)	[62]	14.1	(0.7)	[28]	0.148	1	0.86	-0.22
**HTRA1**	Q92743	14.7	(0.4)	[62]	15.0	(0.8)	[28]	0.048	1	1.24	0.31
**HYAL1**	Q12794	15.9	(0.6)	[30]	15.3	(0.7)	[14]	0.016	1	0.68	-0.56
**HYOU1**	A0A087X054	14.3	(0.3)	[60]	14.2	(0.5)	[19]	0.762	1	0.97	-0.04
**ICAM5**	Q9UMF0	13.1	(0.6)	[59]	12.9	(1.0)	[22]	0.269	1	0.84	-0.25
**ICOSLG**	K4DIA0	16.0	(0.7)	[62]	16.1	(1.0)	[28]	0.659	1	1.07	0.10
**IDS**	P22304	14.7	(0.7)	[61]	14.8	(0.7)	[28]	0.789	1	1.03	0.04
**IGF2**	P01344	14.6	(0.6)	[58]	14.7	(0.5)	[20]	0.181	1	1.13	0.18
**IGFALS**	P35858	14.1	(0.5)	[62]	14.5	(0.6)	[28]	0.003	1	1.32	0.40
**IGFBP2**	P18065	14.0	(0.7)	[62]	14.2	(0.9)	[28]	0.278	1	1.16	0.21

		Communicating HC			Obstructive HC						
**Name**	**Uniprot ID**	**Mean**	**(SD)**	**[N]**	**Mean**	**(SD)**	**[N]**	**P value**	**P adjusted**	**Fold change**	**Log_2(fold change)**
**IGFBP5**	P24593	12.5	(0.6)	[60]	13.0	(0.8)	[24]	0.015	1	1.35	0.43
**IGFBP6**	P24592	17.9	(0.4)	[62]	17.2	(1.0)	[28]	0.003	1	0.64	-0.64
**IGFBP7**	Q16270	17.3	(0.7)	[62]	17.7	(1.5)	[28]	0.15	1	1.36	0.44
**IGHA1**	A0A286YEY1	17.1	(0.9)	[62]	17.1	(0.9)	[28]	0.919	1	1.01	0.01
**IGHA2**	A0A286YEY5	17.1	(0.8)	[62]	17.2	(0.8)	[28]	0.433	1	1.10	0.14
**IGHD**	A0A0A0MS09	14.1	(0.9)	[35]	14.3	(1.4)	[16]	0.676	1	1.11	0.15
**IGHG1**	P01857	19.3	(0.4)	[62]	19.3	(0.5)	[28]	0.874	1	1.01	0.01
**IGHG2**	P01859	19.4	(0.5)	[62]	19.4	(0.5)	[28]	0.999	1	1.00	0.00
**IGHG3**	P01860	16.0	(0.7)	[62]	16.2	(0.7)	[28]	0.206	1	1.16	0.21
**IGHG4**	A0A286YFJ8	15.9	(1.0)	[61]	15.7	(0.8)	[27]	0.52	1	0.91	-0.14
**IGHM**	A0A1B0GUU9	14.7	(1.3)	[62]	15.2	(1.9)	[27]	0.221	1	1.41	0.50
**IGHV1-18**	A0A0C4DH31	14.3	(0.5)	[28]	14.3	(0.5)	[15]	0.688	1	0.96	-0.06
**IGHV1-2**	P23083	15.2	(0.5)	[40]	15.0	(0.6)	[11]	0.624	1	0.93	-0.10
**IGHV1OR15-1**	A0A075B7D0	16.6	(1.3)	[61]	16.7	(1.5)	[28]	0.785	1	1.06	0.08
**IGHV2-26**	A0A0B4J1V2	13.0	(0.8)	[61]	13.1	(0.7)	[21]	0.644	1	1.06	0.08
**IGHV2-5**	P01817	12.5	(1.3)	[23]	12.7	(1.6)	[11]	0.789	1	1.11	0.15
**IGHV3-15**	A0A0B4J1V0	15.4	(0.6)	[62]	15.8	(0.6)	[28]	0.007	1	1.30	0.38
**IGHV3-30**	P01768	16.4	(0.5)	[62]	16.8	(0.5)	[28]	0.007	1	1.26	0.33
**IGHV3-38**	A0A0C4DH36	15.0	(0.6)	[58]	15.1	(0.6)	[27]	0.532	1	1.06	0.08
**IGHV3-49**	A0A0A0MS15	16.3	(0.8)	[62]	16.3	(1.1)	[28]	0.873	1	1.03	0.04
**IGHV3-64D**	A0A0J9YX35	15.8	(0.6)	[60]	16.1	(0.7)	[27]	0.105	1	1.19	0.25

Name	Uniprot ID	Communicating HC Mean	(SD)	[N]	Obstructive HC Mean	(SD)	[N]	P value	P adjusted	Fold change	Log_2(fold change)
**IGHV3-7**	P01780	16.8	(0.4)	[62]	17.1	(0.6)	[28]	0.025	1	1.21	0.28
**IGHV3-72**	A0A0B4J1Y9	16.0	(0.5)	[62]	16.1	(0.7)	[28]	0.609	1	1.06	0.08
**IGHV3OR16-12**	A0A075B7B8	13.0	(0.5)	[38]	12.8	(0.6)	[13]	0.176	1	0.84	-0.25
**IGHV3OR16-9**	A0A0B4J2B5	19.1	(0.8)	[62]	19.4	(0.7)	[28]	0.082	1	1.22	0.29
**IGHV4-34**	P06331	14.5	(0.6)	[62]	14.7	(0.8)	[28]	0.303	1	1.13	0.18
**IGKC**	P01834	19.6	(0.5)	[62]	19.6	(0.5)	[28]	0.944	1	1.01	0.01
**IGKV1-12**	A0A0C4DH73	16.4	(0.7)	[62]	16.5	(0.6)	[28]	0.471	1	1.08	0.11
**IGKV1-16**	P04430	14.4	(0.7)	[30]	14.6	(0.6)	[11]	0.289	1	1.18	0.24
**IGKV1-17**	P01599	15.1	(0.7)	[59]	15.6	(0.6)	[28]	0.006	1	1.35	0.43
**IGKV1-27**	A0A075B6S5	15.3	(0.7)	[39]	15.7	(0.6)	[16]	0.045	1	1.31	0.39
**IGKV1-5**	P01602	15.9	(0.6)	[62]	16.2	(0.6)	[28]	0.018	1	1.26	0.33
**IGKV1-8**	A0A0C4DH67	15.8	(0.5)	[62]	15.9	(0.6)	[28]	0.645	1	1.04	0.06
**IGKV1D-33**	P01593	16.8	(0.8)	[62]	16.9	(0.7)	[28]	0.726	1	1.04	0.06
**IGKV1D-37**	A0A075B6S9	15.8	(1.0)	[35]	15.9	(1.3)	[10]	0.837	1	1.07	0.10
**IGKV2-28**	A0A075B6P5	15.7	(0.6)	[62]	16.0	(0.9)	[28]	0.188	1	1.19	0.25
**IGKV2-29**	A2NJV5	15.6	(0.7)	[62]	16.0	(0.7)	[28]	0.008	1	1.34	0.42
**IGKV2-40**	A0A087WW87	13.5	(0.6)	[28]	13.8	(1.5)	[15]	0.446	1	1.25	0.32
**IGKV2D-24**	A0A075B6R9	16.0	(0.8)	[62]	16.3	(0.8)	[28]	0.044	1	1.30	0.38
**IGKV2D-29**	A0A075B6S2	14.1	(0.7)	[43]	14.6	(0.7)	[17]	0.021	1	1.41	0.50
**IGKV3-15**	P01624	17.5	(0.6)	[62]	17.4	(0.9)	[28]	0.635	1	0.94	-0.09
**IGKV3-20**	P01619	17.8	(0.6)	[62]	17.9	(0.7)	[28]	0.335	1	1.10	0.14

Name	Uniprot ID	Communicating HC Mean	(SD)	[N]	Obstructive HC Mean	(SD)	[N]	P value	P adjusted	Fold change	Log_2(fold change)
**IGKV3-7**	A0A075B6H7	18.4	(1.1)	[57]	19.0	(0.9)	[26]	0.007	1	1.55	0.63
**IGKV3D-11**	A0A0A0MRZ8	17.1	(0.6)	[62]	17.2	(0.6)	[28]	0.766	1	1.03	0.04
**IGKV3D-15**	A0A087WSY6	16.7	(0.8)	[54]	17.0	(1.1)	[20]	0.343	1	1.20	0.26
**IGKV3D-20**	A0A0C4DH25	17.6	(0.8)	[62]	17.7	(0.7)	[28]	0.319	1	1.12	0.16
**IGKV4-1**	P06312	17.0	(0.5)	[62]	16.9	(0.6)	[28]	0.751	1	0.97	-0.04
**IGLC3**	P0DOY3	19.7	(0.6)	[62]	20	(0.8)	[28]	0.045	1	1.28	0.36
**IGLL5**	A0A0B4J231	17.5	(0.5)	[62]	17.6	(0.6)	[28]	0.288	1	1.10	0.14
**IGLV1-47**	P01700	15.9	(0.7)	[62]	16.4	(0.9)	[28]	0.015	1	1.42	0.51
**IGLV1-51**	P01701	14.5	(1.7)	[28]	14.8	(2.3)	[15]	0.625	1	1.26	0.33
**IGLV3-10**	A0A075B6K4	14.7	(0.6)	[47]	15.1	(0.6)	[22]	0.006	1	1.39	0.48
**IGLV3-19**	P01714	14.3	(0.7)	[46]	14.5	(1.1)	[19]	0.589	1	1.11	0.15
**IGLV3-21**	P80748	14.7	(1.1)	[55]	14.8	(1.1)	[24]	0.804	1	1.05	0.07
**IGLV3-25**	P01717	14.3	(0.6)	[61]	14.7	(0.6)	[26]	0.021	1	1.25	0.32
**IGLV3-9**	A0A075B6K5	14.4	(0.6)	[62]	14.5	(0.7)	[28]	0.45	1	1.08	0.11
**IGLV6-57**	P01721	13.6	(0.7)	[46]	13.8	(0.9)	[20]	0.512	1	1.11	0.15
**IGLV7-46**	A0A075B6I9	15.2	(1.1)	[36]	15.7	(1.4)	[12]	0.228	1	1.45	0.54
**IGLV8-61**	A0A075B6I0	14.7	(0.8)	[45]	14.9	(0.9)	[22]	0.402	1	1.15	0.20
**IGSF21**	Q96ID5	14.2	(1.5)	[55]	14.8	(0.7)	[20]	0.029	1	1.48	0.57
**IGSF8**	Q969P0	16.1	(0.4)	[62]	16.3	(0.7)	[28]	0.181	1	1.15	0.20
**IL31RA**	Q8NI17	24.4	(0.6)	[54]	24.3	(1.3)	[23]	0.828	1	0.96	-0.06
**IL6ST**	P40189	14.6	(0.4)	[61]	14.6	(0.5)	[27]	0.746	1	0.97	-0.04

		Communicating HC			Obstructive HC						
**Name**	**Uniprot ID**	**Mean**	**(SD)**	**[N]**	**Mean**	**(SD)**	**[N]**	**P value**	**P adjusted**	**Fold change**	**Log$_2$(fold change)**
**IMPAD1**	Q9NX62	13.9	(0.5)	[62]	14.0	(0.7)	[26]	0.556	1	1.06	0.08
**ISLR**	O14498	15.0	(0.3)	[62]	14.6	(0.7)	[22]	0.029	1	0.77	-0.38
**ISLR2**	Q6UXK2	14.7	(2.3)	[37]	16.1	(2.7)	[10]	0.141	1	2.74	1.45
**ITIH1**	P19827	14.8	(0.5)	[62]	15.1	(0.6)	[28]	0.064	1	1.17	0.23
**ITIH2**	P19823	15.0	(0.4)	[62]	15.1	(0.6)	[28]	0.568	1	1.05	0.07
**ITIH3**	Q06033	13.6	(0.7)	[53]	13.7	(0.7)	[17]	0.811	1	1.03	0.04
**ITIH4**	Q14624	15.4	(0.4)	[62]	15.7	(0.4)	[28]	0.032	1	1.16	0.21
**ITIH5**	C9J2H1	13.8	(0.3)	[61]	14.0	(0.6)	[26]	0.131	1	1.15	0.20
**ITM2B**	Q9Y287	13.6	(1.8)	[62]	14.1	(2.1)	[26]	0.334	1	1.38	0.46
**ITPR2**	Q14571	18.9	(0.8)	[62]	18.9	(0.9)	[28]	0.900	1	0.98	-0.03
**JAM3**	Q9BX67	15.3	(0.6)	[11]	15.7	(0.8)	[10]	0.168	1	1.35	0.43
**JCHAIN**	D6RD17	14.4	(1.2)	[57]	14.9	(1.5)	[25]	0.134	1	1.44	0.53
**KIAA1549L**	H0YDE5	14.4	(0.5)	[62]	14.2	(1.0)	[28]	0.475	1	0.91	-0.14
**KLK6**	Q92876	17.6	(0.5)	[62]	17.9	(0.9)	[28]	0.105	1	1.23	0.30
**KLKB1**	H0YAC1	13.5	(0.5)	[62]	13.8	(0.6)	[28]	0.032	1	1.23	0.30
**KNG1**	P01042	16.6	(0.4)	[62]	16.8	(0.6)	[28]	0.269	1	1.10	0.14
**KRT1**	P04264	15.4	(1.9)	[61]	15.7	(1.6)	[28]	0.508	1	1.20	0.26
**KRT10**	P13645	13.9	(1.5)	[47]	14.5	(1.5)	[23]	0.111	1	1.56	0.64
**KRT14**	P02533	12.1	(2.8)	[31]	12.5	(2.7)	[17]	0.594	1	1.36	0.44
**KRT2**	P35908	14.9	(1.7)	[51]	14.9	(1.8)	[27]	0.885	1	1.04	0.06
**KRT6A**	P02538	13.4	(1.8)	[45]	13.5	(2.0)	[23]	0.814	1	1.09	0.12

Name	Uniprot ID	Communicating HC Mean	(SD)	[N]	Obstructive HC Mean	(SD)	[N]	P value	P adjusted	Fold change	Log_2(fold change)
**KRT77**	Q7Z794	15.4	(1.8)	[45]	15.4	(1.8)	[26]	0.962	1	1.01	0.01
**KRT9**	P35527	14.3	(1.6)	[49]	14.5	(1.4)	[23]	0.587	1	1.15	0.20
**L1CAM**	P32004	12.6	(0.7)	[61]	12.9	(0.9)	[24]	0.296	1	1.16	0.21
**LAMA2**	A0A087WX80	11.1	(1.3)	[44]	11.6	(1.3)	[10]	0.292	1	1.41	0.50
**LAMB2**	P55268	12.4	(0.5)	[44]	12.3	(0.7)	[11]	0.906	1	0.98	-0.03
**LAMC1**	P11047	14.3	(1.7)	[45]	15.3	(1.6)	[12]	0.072	1	2.02	1.01
**LAMP1**	P11279	13.0	(0.5)	[53]	13.4	(0.5)	[22]	0.005	1	1.30	0.38
**LAMP2**	P13473	16.2	(0.6)	[62]	16.2	(0.8)	[28]	0.600	1	1.07	0.10
**LBP**	P18428	13.4	(0.6)	[54]	13.9	(0.6)	[21]	0.008	1	1.33	0.41
**LCAT**	P04180	15.0	(0.6)	[62]	14.9	(0.6)	[19]	0.701	1	0.96	-0.06
**LCP1**	P13796	13.6	(0.6)	[42]	13.7	(0.7)	[18]	0.542	1	1.09	0.12
**LDHA**	P00338	13.5	(0.6)	[62]	13.7	(0.7)	[28]	0.314	1	1.12	0.16
**LDHB**	P07195	14.8	(0.4)	[62]	14.9	(0.6)	[28]	0.422	1	1.07	0.10
**LGALS1**	P09382	14.3	(0.5)	[51]	14.3	(0.5)	[23]	0.895	1	1.01	0.01
**LGALS3**	P17931	12.0	(0.6)	[18]	12.4	(0.5)	[10]	0.131	1	1.26	0.33
**LGALS3BP**	Q08380	17.0	(0.3)	[62]	17.3	(0.7)	[28]	0.027	1	1.24	0.31
**LIAS**	A0A1W2PNQ5	14.4	(0.7)	[41]	14.8	(0.7)	[12]	0.212	1	1.24	0.31
**LINGO1**	Q96FE5	14.6	(0.5)	[32]	14.8	(0.4)	[13]	0.150	1	1.16	0.21
**LMAN2**	D6RBV2	15.4	(0.4)	[62]	15.3	(0.6)	[28]	0.513	1	0.95	-0.07
**LRG1**	P02750	16.3	(0.4)	[62]	16.3	(0.5)	[28]	0.709	1	0.97	-0.04
**LRP1**	Q07954	13.7	(0.5)	[62]	13.7	(0.6)	[23]	0.888	1	1.01	0.01

Name	Uniprot ID	Communicating HC Mean	(SD)	[N]	Obstructive HC Mean	(SD)	[N]	P value	P adjusted	Fold change	Log_2(fold change)
**LRP11**	Q5VYB9	13.6	(0.4)	[21]	13.4	(0.3)	[10]	0.221	1	0.89	-0.17
**LRRC4B**	Q9NT99	14.9	(0.4)	[62]	14.7	(0.7)	[28]	0.098	1	0.85	-0.23
**LSAMP**	H3BLU2	15.4	(0.4)	[62]	15.2	(0.6)	[28]	0.242	1	0.90	-0.15
**LTBP2**	G3V3X5	12.6	(0.5)	[59]	12.2	(0.4)	[13]	0.008	1	0.76	-0.40
**LTF**	E7EQB2	15.2	(1.2)	[58]	16.4	(1.9)	[25]	0.006	1	2.33	1.22
**LUM**	P51884	15.6	(0.3)	[62]	15.4	(0.5)	[28]	0.020	1	0.85	-0.23
**LY6H**	O94772	11.7	(0.8)	[57]	11.9	(1.2)	[16]	0.463	1	1.18	0.24
**LYVE1**	Q9Y5Y7	14.6	(0.5)	[62]	14.5	(0.7)	[28]	0.758	1	0.97	-0.04
**LYZ**	A0A0B4J259	16.9	(0.6)	[62]	16.8	(0.6)	[28]	0.567	1	0.95	-0.07
**MAG**	P20916	13.5	(0.5)	[59]	13.8	(0.7)	[26]	0.046	1	1.24	0.31
**MAN1A1**	P33908	14.2	(0.3)	[62]	14.3	(0.4)	[26]	0.204	1	1.09	0.12
**MAN1C1**	Q9NR34	13.7	(0.5)	[62]	14.0	(0.7)	[24]	0.069	1	1.21	0.28
**MAN2A2**	P49641	14.1	(0.4)	[62]	14.0	(0.6)	[24]	0.725	1	0.97	-0.04
**MAN2B1**	O00754	12.6	(0.3)	[18]	12.7	(0.4)	[13]	0.222	1	1.12	0.16
**MAN2B2**	E9PCD7	14.6	(1.5)	[22]	14.9	(1.5)	[10]	0.612	1	1.22	0.29
**MARCKS**	P29966	11.7	(0.6)	[61]	11.9	(0.7)	[24]	0.228	1	1.16	0.21
**MASP1**	P48740	13.9	(0.8)	[42]	14.1	(0.9)	[21]	0.416	1	1.15	0.20
**MATN2**	O00339	12.8	(0.4)	[32]	13.2	(0.7)	[15]	0.035	1	1.37	0.45
**MCAM**	P43121	14.9	(0.6)	[62]	14.5	(0.9)	[28]	0.027	1	0.74	-0.43
**MDH1**	P40925	15.4	(0.4)	[62]	15.7	(0.7)	[28]	0.072	1	1.19	0.25
**MDH2**	P40926	13.8	(0.8)	[26]	14.3	(1.2)	[11]	0.221	1	1.43	0.52

Name	Uniprot ID	Communicating HC			Obstructive HC			P value	P adjusted	Fold change	Log_2(fold change)
		Mean	(SD)	[N]	Mean	(SD)	[N]				
**MEGF8**	Q7Z7M0	14.4	(0.4)	[61]	14.3	(0.8)	[26]	0.449	1	0.92	-0.12
**MFAP4**	K7ES70	14.5	(0.5)	[61]	14.4	(0.9)	[27]	0.473	1	0.91	-0.14
**MFGE8**	F5GZN3	13.2	(0.6)	[52]	13.5	(0.7)	[12]	0.226	1	1.21	0.28
**MIA**	Q16674	15.2	(0.4)	[36]	15.3	(0.8)	[10]	0.665	1	1.08	0.11
**MIF**	P14174	15.3	(0.5)	[55]	15.6	(0.7)	[24]	0.058	1	1.25	0.32
**MINPP1**	Q9UNW1	14.5	(1.2)	[36]	14.5	(0.8)	[11]	0.942	1	1.02	0.03
**MMP2**	P08253	15.3	(0.3)	[62]	15.1	(0.5)	[28]	0.108	1	0.89	-0.17
**MMRN2**	Q9H8L6	14.4	(0.3)	[47]	14.6	(0.7)	[16]	0.412	1	1.10	0.14
**MOG**	A0A0G2JHA9	14.4	(0.7)	[62]	14.8	(0.8)	[27]	0.025	1	1.31	0.39
**MRC1**	P22897	13.7	(0.7)	[55]	13.6	(0.7)	[16]	0.867	1	0.98	-0.03
**MSN**	P26038	12.1	(1.1)	[40]	13.0	(1.1)	[16]	0.011	1	1.82	0.86
**MST1**	G3XAK1	12.7	(0.6)	[45]	12.8	(0.6)	[14]	0.488	1	1.10	0.14
**MT3**	H3BPK2	10.2	(1.8)	[27]	10.4	(1.4)	[11]	0.711	1	1.15	0.20
**MYH11**	P35749	15.9	(1.1)	[57]	15.4	(1.3)	[25]	0.062	1	0.68	-0.56
**MAATS1**	Q7Z4T9	17.0	(0.5)	[16]	17.0	(0.6)	[11]	0.745	1	0.95	-0.07
**NBL1**	A0A087WTY6	18.3	(0.6)	[62]	18.1	(0.6)	[28]	0.087	1	0.85	-0.23
**NCAM1**	P13591	16.1	(0.3)	[62]	16.2	(0.5)	[28]	0.515	1	1.05	0.07
**NCAM2**	H9KV31	15.6	(0.4)	[62]	15.7	(0.7)	[28]	0.192	1	1.14	0.19
**NCAN**	O14594	15.4	(0.4)	[62]	15.4	(0.6)	[28]	0.526	1	1.05	0.07
**NCSTN**	Q92542	12.0	(0.8)	[37]	12.5	(0.8)	[15]	0.024	1	1.47	0.56
**NDRG2**	Q9UN36-2	13.7	(0.8)	[54]	13.5	(1.1)	[16]	0.485	1	0.86	-0.22

Name	Uniprot ID	Communicating HC Mean	(SD)	[N]	Obstructive HC Mean	(SD)	[N]	P value	P adjusted	Fold change	Log_2(fold change)
**NECTIN1**	Q15223	14.4	(0.3)	[62]	14.5	(0.6)	[26]	0.640	1	1.04	0.06
**NEGR1**	Q7Z3B1	15.7	(0.7)	[62]	15.3	(1.0)	[28]	0.079	1	0.78	-0.36
**NELL2**	F8VVB6	15.7	(0.4)	[62]	15.4	(0.8)	[28]	0.144	1	0.85	-0.23
**NEO1**	Q92859	15.1	(0.5)	[62]	15.2	(0.5)	[28]	0.874	1	1.01	0.01
**NFASC**	O94856	15.0	(0.4)	[62]	15.0	(0.5)	[28]	0.462	1	1.06	0.08
**NID1**	P14543	13.5	(0.3)	[61]	13.6	(0.4)	[15]	0.342	1	1.08	0.11
**NID2**	Q14112	14.0	(0.5)	[56]	14.4	(0.7)	[26]	0.021	1	1.30	0.38
**NLGN4X**	A0A0A0MTH0	12.0	(1.5)	[42]	12.3	(1.5)	[17]	0.465	1	1.25	0.32
**NOMO1**	A0A087X117	14.1	(1.7)	[34]	14.7	(2.4)	[11]	0.483	1	1.48	0.57
**NPC2**	E7EMS2	16.9	(0.4)	[62]	17.1	(0.8)	[28]	0.079	1	1.22	0.29
**NPDC1**	Q5SPY9	13.8	(0.5)	[62]	13.8	(1.0)	[26]	0.866	1	1.02	0.03
**NPPC**	P23582	14.1	(0.5)	[62]	14.0	(0.6)	[23]	0.413	1	0.92	-0.12
**NPTX1**	Q15818	16.0	(0.7)	[62]	15.6	(1.0)	[28]	0.060	1	0.76	-0.40
**NPTX2**	P47972	13.5	(0.7)	[36]	13.8	(0.7)	[11]	0.239	1	1.24	0.31
**NPTXR**	A0A1X7SBT7	15.6	(0.5)	[62]	15.9	(0.7)	[28]	0.077	1	1.21	0.28
**NPY**	P01303	14.5	(1.1)	[59]	14.4	(1.8)	[26]	0.745	1	0.92	-0.12
**NRCAM**	C9JYY6	16.2	(0.4)	[62]	16.0	(0.7)	[28]	0.329	1	0.91	-0.14
**NRN1**	A0A087WWT2	15.5	(0.3)	[61]	15.2	(0.6)	[28]	0.021	1	0.82	-0.29
**NRP1**	E7EX60	12.8	(0.5)	[60]	12.9	(0.4)	[15]	0.386	1	1.07	0.10
**NRP2**	A0A024R3W6	12.6	(0.3)	[40]	12.6	(0.4)	[11]	0.933	1	1.01	0.01
**NRXN1**	A0A0D9SEP4	14.1	(0.3)	[61]	14.3	(0.7)	[27]	0.160	1	1.16	0.21

Name	Uniprot ID	Communicating HC Mean	(SD)	[N]	Obstructive HC Mean	(SD)	[N]	P value	P adjusted	Fold change	Log_2(fold change)
**NRXN2**	G5E9G7	15.0	(0.6)	[62]	15.1	(0.6)	[28]	0.640	1	1.05	0.07
**NRXN3**	A0A0U1RQC5	14.5	(0.6)	[62]	14.5	(0.8)	[28]	0.791	1	0.97	-0.04
**NSG1**	A0A0A6YYJ2	11.5	(0.8)	[55]	11.4	(0.8)	[19]	0.693	1	0.94	-0.09
**NTM**	Q9P121-4	15.2	(0.7)	[62]	15.4	(0.9)	[28]	0.317	1	1.14	0.19
**NTNG1**	Q9Y2I2	12.3	(0.8)	[45]	12.1	(0.8)	[14]	0.435	1	0.87	-0.20
**NTRK2**	Q16620	15.2	(0.5)	[44]	15.1	(0.6)	[16]	0.744	1	0.96	-0.06
**NUCB1**	Q02818	14.0	(0.3)	[62]	14.4	(0.7)	[28]	0.019	1	1.28	0.36
**OAF**	Q86UD1	14.9	(0.3)	[62]	14.8	(0.6)	[26]	0.333	1	0.92	-0.12
**OLA1**	J3KQ32	15.4	(1.5)	[22]	14.9	(1.3)	[10]	0.318	1	0.70	-0.51
**OLFM1**	Q99784	14.1	(0.4)	[57]	14.2	(0.5)	[22]	0.212	1	1.11	0.15
**OLFML3**	B4DNG0	12.6	(0.8)	[39]	13.1	(0.8)	[13]	0.101	1	1.36	0.44
**OMD**	Q99983	13.8	(0.7)	[61]	14.1	(0.6)	[16]	0.182	1	1.19	0.25
**OMG**	P23515	16.1	(0.4)	[62]	16.2	(0.6)	[28]	0.491	1	1.07	0.10
**ORM1**	P02763	19.0	(0.3)	[62]	18.7	(0.5)	[28]	0.013	1	0.83	-0.27
**ORM2**	P19652	17.6	(0.4)	[62]	17.3	(0.5)	[28]	0.035	1	0.86	-0.22
**PAM**	P19021	15.4	(0.3)	[62]	15.3	(0.6)	[28]	0.794	1	0.98	-0.03
**PAPLN**	O95428	13.3	(0.3)	[45]	13.2	(0.6)	[14]	0.636	1	0.95	-0.07
**PARK7**	Q99497	13.0	(1.0)	[31]	13.5	(1.0)	[15]	0.086	1	1.46	0.55
**PCDH1**	Q08174	13.4	(0.5)	[58]	13.5	(0.7)	[17]	0.901	1	1.02	0.03
**PCDH7**	O60245	12.6	(0.7)	[44]	13.1	(1.3)	[12]	0.178	1	1.46	0.55
**PCDH9**	B7ZM79	12.9	(0.9)	[38]	13.2	(0.5)	[13]	0.114	1	1.25	0.32

		Communicating HC			Obstructive HC						
**Name**	**Uniprot ID**	**Mean**	**(SD)**	**[N]**	**Mean**	**(SD)**	**[N]**	**P value**	**P adjusted**	**Fold change**	**Log$_2$(fold change)**
**PCDHAC2**	Q9Y5I4	13.4	(0.9)	[50]	14.1	(1.5)	[12]	0.143	1	1.63	0.70
**PCMT1**	A0A0A0MRJ6	13.6	(1.6)	[42]	14.0	(1.4)	[14]	0.337	1	1.36	0.44
**PCSK1**	P29120	13.2	(2.3)	[44]	14.7	(1.8)	[15]	0.015	1	2.82	1.50
**PCSK1N**	Q9UHG2	15.7	(0.5)	[62]	15.6	(0.6)	[28]	0.69	1	0.96	-0.06
**PDGFA**	A0A0A0MSC4	15.4	(1.3)	[43]	15.6	(1.8)	[22]	0.709	1	1.12	0.16
**PDGFB**	A9UJN9	14.1	(0.7)	[49]	14.4	(1.0)	[17]	0.427	1	1.17	0.23
**PDIA3**	P30101	13.5	(0.5)	[53]	13.8	(0.5)	[21]	0.038	1	1.22	0.29
**PEA15**	Q15121	13.6	(0.6)	[50]	14.0	(0.7)	[17]	0.061	1	1.28	0.36
**PEBP1**	P30086	16.1	(0.4)	[62]	16.3	(0.6)	[28]	0.09	1	1.17	0.23
**PEBP4**	Q96S96	16.4	(0.5)	[62]	16.2	(0.5)	[28]	0.164	1	0.89	-0.17
**PENK**	P01210	15.6	(0.5)	[62]	15.7	(0.8)	[28]	0.581	1	1.06	0.08
**PEPD**	P12955	12.5	(0.4)	[58]	12.5	(0.5)	[25]	0.653	1	1.04	0.06
**PFN1**	P07737	15.1	(0.7)	[44]	15.1	(0.7)	[19]	0.805	1	0.97	-0.04
**PGAM1**	P18669	14.6	(0.7)	[62]	14.9	(0.8)	[28]	0.075	1	1.25	0.32
**PGK1**	P00558	13.8	(0.7)	[58]	14.1	(1.0)	[24]	0.24	1	1.20	0.26
**PGLYRP2**	Q96PD5	14.5	(0.4)	[62]	14.5	(0.5)	[25]	0.475	1	0.95	-0.07
**PI16**	Q6UXB8	13.8	(0.6)	[62]	13.8	(1.0)	[28]	0.874	1	0.98	-0.03
**PIK3IP1**	Q96FE7-4	15.3	(0.4)	[62]	15.4	(0.4)	[27]	0.263	1	1.08	0.11
**PKM**	P14618	14.6	(0.4)	[62]	14.6	(0.6)	[28]	0.608	1	0.95	-0.07
**PLD3**	Q8IV08	14.9	(0.6)	[62]	15.0	(0.4)	[27]	0.294	1	1.08	0.11
**PLD4**	F5H2B5	13.7	(0.6)	[53]	13.7	(0.7)	[19]	0.750	1	0.96	-0.06

Name	Uniprot ID	Communicating HC Mean	(SD)	[N]	Obstructive HC Mean	(SD)	[N]	P value	P adjusted	Fold change	Log_2(fold change)
**PLG**	P00747	15.9	(0.3)	[62]	16.1	(0.4)	[28]	0.041	1	1.13	0.18
**PLOD1**	Q02809	12.9	(0.5)	[49]	13.1	(0.7)	[24]	0.145	1	1.18	0.24
**PLTP**	P55058	16.0	(0.3)	[62]	15.8	(0.4)	[28]	0.072	1	0.89	-0.17
**PLXDC2**	Q6UX71	15.3	(0.4)	[62]	15.4	(0.6)	[28]	0.214	1	1.12	0.16
**PLXNB1**	O43157	13.1	(0.5)	[58]	13.6	(0.6)	[19]	0.005	1	1.36	0.44
**PLXNB2**	O15031	14.0	(0.5)	[59]	14.3	(0.7)	[24]	0.044	1	1.24	0.31
**PMFBP1**	G3V1Q7	16.8	(0.3)	[49]	16.9	(0.5)	[18]	0.388	1	1.07	0.10
**PODXL2**	Q9NZ53	12.6	(0.9)	[54]	12.1	(0.9)	[14]	0.087	1	0.73	-0.45
**POMGNT1**	Q8WZA1	14.3	(0.5)	[62]	14.3	(0.6)	[24]	0.653	1	0.96	-0.06
**PON1**	P27169	15.2	(0.6)	[62]	15.4	(0.8)	[28]	0.191	1	1.18	0.24
**PPIA**	P62937	15.2	(0.6)	[62]	15.5	(0.9)	[28]	0.098	1	1.25	0.32
**PPIB**	P23284	15.1	(0.5)	[62]	15.4	(0.7)	[28]	0.158	1	1.17	0.23
**PPIC**	P45877	14.8	(0.3)	[60]	14.8	(0.5)	[27]	0.775	1	0.98	-0.03
**PPT1**	A0A286YFE3	12.5	(0.5)	[47]	12.5	(0.3)	[17]	0.953	1	1.00	0.00
**PRCP**	P42785	15.2	(0.6)	[62]	15.5	(0.8)	[28]	0.062	1	1.26	0.33
**PRDX1**	Q06830	15.6	(0.9)	[60]	16.1	(1.1)	[27]	0.039	1	1.44	0.53
**PRDX2**	P32119	14.9	(0.8)	[62]	15.7	(1.5)	[28]	0.006	1	1.82	0.86
**PRDX3**	P30048	13.0	(1.0)	[16]	13.0	(0.9)	[11]	0.887	1	1.04	0.06
**PRDX6**	P30041	14.5	(1.0)	[60]	14.7	(1.3)	[27]	0.363	1	1.20	0.26
**PRELP**	P51888	13.8	(0.4)	[61]	13.6	(0.9)	[21]	0.337	1	0.87	-0.20
**PRG4**	A0A0U1RR20	12.6	(0.8)	[40]	13.2	(0.6)	[11]	0.011	1	1.57	0.65

Name	Uniprot ID	Communicating HC Mean	(SD)	[N]	Obstructive HC Mean	(SD)	[N]	P value	P adjusted	Fold change	Log$_2$(fold change)
**PRKCSH**	K7ELL7	15.1	(0.5)	[50]	14.6	(0.8)	[17]	0.024	1	0.70	-0.51
**PRL**	A6XMH3	12.6	(0.9)	[45]	13.2	(1.4)	[14]	0.206	1	1.45	0.54
**PRNP**	A2A2V1	14.1	(0.5)	[62]	14.3	(0.7)	[28]	0.123	1	1.17	0.23
**PROCR**	Q9UNN8	14.4	(0.8)	[62]	14.6	(0.7)	[28]	0.260	1	1.14	0.19
**PROS1**	P07225	15.3	(0.3)	[62]	15.6	(0.5)	[28]	0.010	1	1.23	0.30
**PROZ**	P22891	13.1	(0.7)	[32]	13.5	(0.6)	[12]	0.059	1	1.33	0.41
**PRSS1**	E7EQ64	16.1	(0.6)	[31]	16.4	(0.9)	[18]	0.222	1	1.23	0.30
**PRSS3**	B1AN99	20.5	(1.6)	[20]	21.3	(2.3)	[16]	0.242	1	1.74	0.80
**PSAP**	C9JIZ6	14.2	(0.8)	[62]	14.7	(1.0)	[26]	0.074	1	1.34	0.42
**PSAT1**	Q9Y617	13.8	(0.7)	[52]	14.3	(0.8)	[23]	0.011	1	1.41	0.50
**PTGDS**	P41222	20.9	(0.3)	[62]	20.8	(0.5)	[28]	0.188	1	0.91	-0.14
**PTPRD**	P23468	14.6	(0.5)	[62]	14.9	(0.6)	[28]	0.069	1	1.19	0.25
**PTPRF**	P10586	13.4	(1.3)	[46]	14.1	(1.5)	[15]	0.116	1	1.65	0.72
**PTPRG**	P23470	15.2	(0.3)	[62]	15.0	(0.6)	[28]	0.069	1	0.85	-0.23
**PTPRK**	E9PGC5	12.4	(0.5)	[47]	12.8	(0.6)	[15]	0.05	1	1.28	0.36
**PTPRN**	Q16849	11.6	(2.2)	[26]	12.4	(2.1)	[12]	0.343	1	1.66	0.73
**PTPRN2**	Q92932	15.5	(0.5)	[62]	15.6	(0.7)	[28]	0.424	1	1.09	0.12
**PTPRS**	Q13332	14.7	(0.3)	[62]	15.0	(0.6)	[28]	0.023	1	1.23	0.30
**PTPRZ1**	P23471	15.3	(0.5)	[62]	15.6	(0.8)	[28]	0.053	1	1.24	0.31
**PVALB**	B8ZZ19	13.4	(0.7)	[37]	13.5	(0.9)	[10]	0.728	1	1.08	0.11
**QDPR**	P09417	14.6	(0.8)	[59]	15.0	(1.0)	[26]	0.077	1	1.33	0.41

Name	Uniprot ID	Communicating HC Mean	(SD)	[N]	Obstructive HC Mean	(SD)	[N]	P value	P adjusted	Fold change	Log_2(fold change)
**QPCT**	Q16769	13.9	(0.6)	[62]	14.1	(0.7)	[28]	0.164	1	1.16	0.21
**QSOX1**	O00391	14.4	(0.3)	[62]	14.6	(0.5)	[28]	0.099	1	1.13	0.18
**RARRES2**	Q99969	16.1	(0.4)	[62]	16.0	(1.0)	[28]	0.650	1	0.94	-0.09
**RBP4**	P02753	16.3	(0.4)	[62]	16.4	(0.6)	[28]	0.413	1	1.08	0.11
**RELN**	J3KQ66	14.8	(0.6)	[58]	14.5	(0.8)	[18]	0.243	1	0.85	-0.23
**RGMA**	A0A0A0MTQ4	14.8	(0.5)	[37]	14.7	(1.3)	[11]	0.85	1	0.95	-0.07
**RGMB**	J3KNF6	14.9	(0.6)	[62]	14.8	(0.9)	[24]	0.491	1	0.91	-0.14
**RNASE1**	P07998	12.5	(0.6)	[62]	12.5	(1.4)	[28]	0.816	1	1.04	0.06
**RNASE4**	P34096	13.7	(0.4)	[61]	13.7	(0.4)	[22]	0.952	1	1.00	0.00
**RNASE6**	Q93091	13.9	(0.7)	[57]	14.2	(0.7)	[25]	0.050	1	1.26	0.33
**RNASET2**	A0A087WZM2	15.0	(0.4)	[62]	15.4	(0.7)	[28]	0.012	1	1.32	0.40
**RNF13**	C9J8T4	14.3	(0.7)	[32]	14.9	(0.7)	[17]	0.004	1	1.53	0.61
**ROBO1**	Q9Y6N7	14.0	(0.6)	[61]	13.7	(0.8)	[20]	0.168	1	0.82	-0.29
**RP1**	P56715	14.7	(0.7)	[35]	14.7	(0.8)	[12]	0.917	1	0.98	-0.03
**RTN4R**	H7C0V4	13.6	(0.6)	[43]	13.1	(1.7)	[14]	0.257	1	0.69	-0.54
**RTN4RL2**	Q86UN3	14.5	(0.5)	[55]	14.5	(0.7)	[26]	0.900	1	0.99	-0.01
**S100A1**	P23297	12.7	(0.8)	[34]	13.5	(1.0)	[15]	0.010	1	1.80	0.85
**S100A9**	P06702	14.8	(1.7)	[10]	14.6	(1.1)	[11]	0.778	1	0.88	-0.18
**S100B**	P04271	15.3	(1.2)	[61]	15.1	(1.5)	[27]	0.520	1	0.86	-0.22
**SCG2**	P13521	14.6	(0.5)	[62]	14.9	(0.7)	[28]	0.059	1	1.22	0.29
**SCG3**	Q8WXD2	16.1	(0.4)	[62]	16.1	(0.6)	[28]	0.504	1	0.94	-0.09

Name	Uniprot ID	Communicating HC Mean	(SD)	[N]	Obstructive HC Mean	(SD)	[N]	P value	P adjusted	Fold change	Log_2(fold change)
**SCG5**	P05408-2	16.2	(0.3)	[62]	16.5	(0.6)	[28]	0.019	1	1.25	0.32
**SCRG1**	O75711	17.2	(0.7)	[62]	17.3	(1.0)	[28]	0.670	1	1.06	0.08
**SDF4**	Q9BRK5	13.6	(0.9)	[52]	14.1	(0.9)	[22]	0.030	1	1.45	0.54
**SELENBP1**	Q13228	14.7	(0.4)	[62]	15.0	(0.6)	[28]	0.021	1	1.24	0.31
**SELENOM**	A0A087WWF1	13.3	(0.6)	[35]	13.0	(0.9)	[14]	0.220	1	0.79	-0.34
**SELENOP**	A0A182DWH7	15.6	(0.5)	[62]	15.8	(0.6)	[25]	0.166	1	1.14	0.19
**SELL**	P14151	15.1	(0.4)	[62]	15.1	(0.4)	[26]	0.595	1	1.03	0.04
**SEMA3C**	Q99985	13.1	(0.4)	[36]	13.2	(0.6)	[15]	0.598	1	1.07	0.10
**SEMA3G**	Q9NS98	13.1	(0.8)	[46]	12.8	(1.0)	[12]	0.457	1	0.85	-0.23
**SEMA4B**	J3KNP4	13.8	(0.4)	[60]	13.7	(0.7)	[21]	0.513	1	0.93	-0.10
**SEMA6A**	A0A0A0MQU6	13.2	(1.4)	[35]	13.6	(1.3)	[20]	0.360	1	1.27	0.34
**SEMA7A**	O75326	14.8	(0.7)	[62]	15.0	(0.8)	[28]	0.101	1	1.22	0.29
**SERPINA1**	P01009	18.0	(0.3)	[62]	18.0	(0.4)	[28]	0.661	1	0.97	-0.04
**SERPINA10**	G3V2W1	11.8	(1.0)	[25]	12.1	(1.1)	[10]	0.361	1	1.29	0.37
**SERPINA3**	P01011	17.1	(0.3)	[62]	17.1	(0.5)	[28]	0.597	1	1.04	0.06
**SERPINA4**	P29622	14.9	(0.3)	[62]	14.9	(0.3)	[28]	0.526	1	1.03	0.04
**SERPINA5**	P05154	13.5	(0.8)	[60]	14.1	(1.1)	[28]	0.019	1	1.49	0.58
**SERPINA6**	P08185	15.0	(0.3)	[62]	15.2	(0.5)	[28]	0.05	1	1.14	0.19
**SERPINA7**	P05543	14.8	(0.4)	[62]	14.9	(0.4)	[28]	0.71	1	1.02	0.03
**SERPINB6**	A0A024QZX5	13.2	(0.5)	[50]	13.4	(0.6)	[17]	0.196	1	1.16	0.21
**SERPINC1**	P01008	16.6	(0.3)	[62]	16.7	(0.3)	[28]	0.571	1	1.03	0.04

Name	Uniprot ID	Communicating HC Mean	(SD)	[N]	Obstructive HC Mean	(SD)	[N]	P value	P adjusted	Fold change	Log_2(fold change)
**SERPIND1**	P05546	15.3	(0.3)	[62]	15.4	(0.3)	[28]	0.288	1	1.05	0.07
**SERPINF1**	P36955	17.8	(0.4)	[62]	18.1	(0.9)	[28]	0.061	1	1.28	0.36
**SERPING1**	P05155	16.3	(0.2)	[62]	16.3	(0.3)	[28]	0.673	1	1.02	0.03
**SERPINI1**	Q99574	14.9	(0.6)	[62]	15.1	(0.7)	[28]	0.111	1	1.19	0.25
**SEZ6**	Q53EL9	13.9	(0.6)	[59]	13.9	(0.6)	[16]	0.906	1	1.01	0.01
**SEZ6L**	B0QYH4	14.3	(0.4)	[62]	14.3	(0.6)	[27]	0.918	1	0.99	-0.01
**SEZ6L2**	A0A087WYL5	15.4	(0.4)	[62]	15.3	(0.6)	[28]	0.532	1	0.95	-0.07
**SGCE**	B7Z2R4	12.8	(0.8)	[42]	13.1	(0.9)	[15]	0.383	1	1.18	0.24
**SH3BGRL**	O75368	12.5	(0.5)	[27]	13.0	(0.6)	[16]	0.004	1	1.48	0.57
**SH3BGRL3**	Q5T123	12.7	(0.6)	[22]	13.5	(0.9)	[15]	0.005	1	1.75	0.81
**SHBG**	I3L145	13.5	(0.4)	[61]	13.8	(0.6)	[25]	0.044	1	1.22	0.29
**SHISA6**	Q6ZSJ9	14.1	(0.5)	[58]	14.0	(0.7)	[22]	0.512	1	0.93	-0.10
**SIAE**	Q9HAT2	14.8	(0.7)	[60]	15.6	(1.2)	[27]	0.002	1	1.75	0.81
**SIRPA**	P78324	15.0	(0.5)	[62]	15.2	(0.6)	[28]	0.084	1	1.17	0.23
**SKP1**	E5RJR5	13.9	(0.8)	[61]	14.2	(0.6)	[27]	0.075	1	1.21	0.28
**SLC39A10**	Q9ULF5	12.5	(0.7)	[48]	13.1	(1.0)	[12]	0.077	1	1.48	0.57
**SLC39A12**	Q504Y0	12.5	(0.4)	[27]	12.6	(0.6)	[10]	0.727	1	1.05	0.07
**SLC3A2**	F5GZS6	13.0	(0.6)	[58]	13.0	(0.6)	[21]	0.955	1	0.99	-0.01
**SLITRK1**	Q96PX8	13.2	(0.6)	[49]	13.7	(1.0)	[13]	0.149	1	1.37	0.45
**SLITRK3**	O94933	13.6	(0.4)	[49]	13.4	(0.4)	[11]	0.175	1	0.87	-0.20
**SLITRK4**	Q8IW52	13.5	(0.5)	[61]	13.3	(0.9)	[20]	0.421	1	0.89	-0.17

Name	Uniprot ID	Communicating HC Mean	(SD)	[N]	Obstructive HC Mean	(SD)	[N]	P value	P adjusted	Fold change	Log$_2$(fold change)
**SOD1**	P00441	16.2	(0.3)	[62]	16.2	(0.5)	[28]	0.635	1	1.03	0.04
**SOD2**	P04179	13.7	(0.4)	[61]	13.7	(0.5)	[28]	0.843	1	0.99	-0.01
**SOD3**	P08294	16.1	(0.6)	[62]	16.4	(1.2)	[28]	0.332	1	1.17	0.23
**SORCS3**	Q9UPU3	13.4	(0.8)	[60]	13.8	(1.0)	[25]	0.140	1	1.27	0.34
**SORL1**	Q92673	13.6	(0.7)	[37]	14.3	(1.2)	[20]	0.052	1	1.53	0.61
**SORT1**	Q99523	14.2	(0.4)	[61]	14.3	(0.7)	[26]	0.236	1	1.12	0.16
**SPARC**	P09486	16.6	(0.8)	[62]	17.1	(1.5)	[28]	0.088	1	1.43	0.52
**SPARCL1**	Q14515	15.3	(0.3)	[62]	15.5	(0.4)	[28]	0.050	1	1.13	0.18
**SPINT2**	K7EM91	12.9	(0.5)	[50]	12.9	(0.8)	[18]	0.913	1	0.98	-0.03
**SPOCK1**	Q08629	14.3	(0.5)	[62]	14.5	(0.7)	[27]	0.129	1	1.16	0.21
**SPOCK2**	Q92563	14.0	(0.7)	[50]	14.0	(0.6)	[22]	0.824	1	1.03	0.04
**SPOCK3**	Q9BQ16	15.2	(0.6)	[61]	15.6	(0.9)	[28]	0.035	1	1.34	0.42
**SPON1**	Q9HCB6	14.4	(0.6)	[62]	14.5	(0.9)	[24]	0.469	1	1.10	0.14
**SPP1**	P10451	16.2	(0.3)	[62]	16.3	(0.6)	[28]	0.253	1	1.10	0.14
**SRGN**	P10124	15.5	(0.4)	[19]	16.2	(0.9)	[17]	0.006	1	1.62	0.70
**ST6GAL2**	Q96JF0	13.8	(0.5)	[18]	13.5	(1.0)	[11]	0.351	1	0.8	-0.32
**SULF2**	Q8IWU5	15.0	(0.4)	[62]	14.9	(0.9)	[28]	0.924	1	0.99	-0.01
**SUSD5**	O60279	12.9	(0.4)	[62]	13.1	(0.7)	[22]	0.348	1	1.11	0.15
**SYNE3**	G3V533	14.5	(0.7)	[47]	14.6	(0.7)	[18]	0.642	1	1.07	0.10
**SYT2**	Q8N9I0	17.5	(0.8)	[56]	17.7	(1.0)	[22]	0.33	1	1.18	0.24
**SAA4**	P35542	14.7	(0.6)	[62]	15.0	(0.6)	[28]	0.065	1	1.19	0.25

		Communicating HC			Obstructive HC						
**Name**	**Uniprot ID**	**Mean**	**(SD)**	**[N]**	**Mean**	**(SD)**	**[N]**	**P value**	**P adjusted**	**Fold change**	**Log$_2$(fold change)**
**TAC1**	P20366	14.6	(0.6)	[40]	15.0	(0.9)	[17]	0.099	1	1.34	0.42
**TAGLN**	Q01995	14.1	(0.6)	[62]	13.9	(0.6)	[16]	0.232	1	0.86	-0.22
**TALDO1**	P37837	13.6	(1.1)	[57]	14.0	(1.7)	[15]	0.337	1	1.38	0.46
**TCN2**	B5MBX2	15.5	(1.2)	[60]	15.8	(1.9)	[24]	0.444	1	1.25	0.32
**TF**	P02787	17.9	(0.2)	[62]	17.9	(0.3)	[28]	0.541	1	0.97	-0.04
**TGFBI**	Q15582	14.5	(0.3)	[62]	14.7	(0.4)	[28]	0.044	1	1.14	0.19
**TGFBR3**	Q03167	12.9	(0.6)	[56]	13.0	(0.6)	[19]	0.461	1	1.08	0.11
**TGOLN2**	F8W8W7	12.6	(1.2)	[61]	12.7	(1.2)	[28]	0.848	1	1.04	0.06
**THBS2**	P35442	13.8	(0.5)	[43]	13.9	(0.6)	[21]	0.679	1	1.05	0.07
**THY1**	E9PIM6	17.7	(0.6)	[62]	17.8	(0.6)	[28]	0.814	1	1.02	0.03
**TIMP1**	P01033	16.7	(0.6)	[58]	16.5	(0.7)	[23]	0.204	1	0.86	-0.22
**TIMP2**	P16035	15.9	(0.3)	[60]	16.2	(0.9)	[24]	0.094	1	1.24	0.31
**TKT**	P29401	13.8	(0.8)	[37]	14.1	(1.0)	[15]	0.258	1	1.25	0.32
**TMEM132A**	Q24JP5	14.1	(0.5)	[62]	14.1	(0.8)	[21]	0.956	1	1.01	0.01
**TNC**	P24821	12.5	(0.6)	[48]	13.1	(1.0)	[23]	0.009	1	1.56	0.64
**TNFRSF21**	O75509	14.7	(0.4)	[55]	14.8	(0.5)	[26]	0.688	1	1.03	0.04
**TNXB**	A0A140TA41	13.2	(0.6)	[62]	13.2	(0.8)	[18]	0.931	1	1.01	0.01
**TPP1**	O14773	14.9	(0.7)	[62]	15.3	(1.0)	[28]	0.036	1	1.37	0.45
**TPP2**	P29144	14.7	(0.9)	[62]	14	(0.9)	[28]	0.003	1	0.63	-0.67
**TPT1**	A0A0B4J2C3	16.8	(0.4)	[28]	16.8	(0.7)	[17]	0.881	1	0.98	-0.03
**TRBV7-2**	A0A1B0GXF2	15.1	(0.5)	[54]	15.0	(0.6)	[13]	0.654	1	0.94	-0.09

Name	Uniprot ID	Communicating HC Mean	(SD)	[N]	Obstructive HC Mean	(SD)	[N]	P value	P adjusted	Fold change	Log_2(fold change)
**TREM2**	Q9NZC2	13.7	(0.9)	[60]	13.9	(1.1)	[23]	0.412	1	1.16	0.21
**TTR**	P02766	19.9	(0.8)	[62]	20.1	(1.4)	[28]	0.454	1	1.15	0.20
**TUBA1B**	P68363	16.5	(1.1)	[61]	16.1	(1.7)	[27]	0.329	1	0.78	-0.36
**TUBB**	P07437	15.8	(1.0)	[58]	16.1	(0.8)	[21]	0.177	1	1.23	0.30
**TUBB4B**	P68371	15.8	(1.0)	[61]	15.7	(1.3)	[26]	0.631	1	0.91	-0.14
**TWSG1**	J3QS03	14.6	(0.2)	[56]	14.7	(0.5)	[25]	0.73	1	1.02	0.03
**TXN**	P10599	16.2	(0.7)	[60]	16.7	(0.7)	[27]	0.003	1	1.40	0.49
**TXNDC17**	Q9BRA2	13.7	(0.3)	[47]	14.0	(0.4)	[14]	0.021	1	1.26	0.33
**UBC**	F5H265	16.2	(0.3)	[62]	16.4	(0.7)	[28]	0.061	1	1.20	0.26
**UBE2N**	P61088	13.1	(0.7)	[13]	13.3	(0.8)	[12]	0.514	1	1.15	0.20
**VASN**	Q6EMK4	14.7	(0.2)	[62]	14.8	(0.5)	[28]	0.300	1	1.07	0.10
**VCAM1**	P19320	13.7	(0.4)	[60]	13.8	(0.5)	[19]	0.832	1	1.02	0.03
**VCAN**	P13611	14.0	(0.4)	[62]	14.1	(0.6)	[28]	0.382	1	1.08	0.11
**VGF**	O15240	15.3	(0.4)	[62]	15.6	(0.8)	[28]	0.045	1	1.26	0.33
**VIM**	P08670	12.8	(1.0)	[49]	13.5	(1.5)	[17]	0.118	1	1.57	0.65
**VIP**	P01282	17.8	(0.4)	[62]	17.7	(0.5)	[28]	0.907	1	0.99	-0.01
**VSIG4**	Q9Y279	13.2	(0.6)	[62]	13.0	(0.7)	[27]	0.275	1	0.88	-0.18
**VSTM2A**	B5MCX6	14.7	(0.5)	[62]	14.5	(1.4)	[28]	0.609	1	0.90	-0.15
**VSTM2B**	A6NLU5	14.1	(1.1)	[62]	14.1	(0.9)	[27]	0.951	1	0.99	-0.01
**VTN**	P04004	16.9	(0.4)	[62]	17.0	(0.4)	[28]	0.068	1	1.13	0.18
**VWF**	P04275	14.0	(1.1)	[21]	14.4	(2.1)	[10]	0.643	1	1.26	0.33

Name	Uniprot ID	Communicating HC Mean	(SD)	[N]	Obstructive HC Mean	(SD)	[N]	P value	P adjusted	Fold change	Log_2(fold change)
**WFIKKN2**	C9J6G4	15.4	(0.7)	[62]	15.7	(0.9)	[28]	0.168	1	1.20	0.26
**XYLT1**	Q86Y38	13.8	(1.1)	[19]	13.5	(1.0)	[12]	0.428	1	0.81	-0.30
**YWHAE**	P62258	13.6	(0.8)	[61]	14.1	(1.3)	[27]	0.061	1	1.43	0.52
**YWHAG**	P61981	13.3	(1.5)	[33]	13.8	(1.7)	[18]	0.327	1	1.39	0.48
**YWHAQ**	P27348	13.7	(1.0)	[48]	13.9	(1.1)	[23]	0.490	1	1.14	0.19
**YWHAZ**	P63104	14.0	(0.6)	[62]	14.3	(1.0)	[28]	0.111	1	1.25	0.32
**ZNF511-PRAP1**	H7BY64	14.1	(0.6)	[59]	14.5	(1.1)	[26]	0.116	1	1.29	0.37
