# Supplementary material for: Ventricular CSF proteomic profiles and predictors of surgical treatment outcome in chronic hydrocephalus
Source: Acta Neurochir (Wien). 2023 Oct 19;165(12):4059–70. doi: 10.1007/s00701-023-05832-y (PMC10739511; doi:10.1007/s00701-023-05832-y)
Supplement: Supplementary file 5 — Supplementary file5 (PDF 40 KB) [file 701_2023_5832_MOESM5_ESM.pdf]

Supplemental file 5

Proteins higher in obstructive hydrocephalus (HC) compared to communicating HC but not significant

		Communicating HC			Obstructive HC						
Name	Uniprot ID	Mean	(SD)	[N]	Mean	(SD)	[N]	P value	P adjusted	Fold change	Log_2(fold change)
**HBB**	P68871	17.3	(1.6)	[62]	19.1	(2.2)	[28]	<0.001	0.342	3.42	1.77
**HBA1**	P69905	17.6	(1.5)	[62]	19.2	(2.2)	[28]	0.001	0.784	2.99	1.58
**BLVRB**	P30043	12.7	(1.1)	[23]	14.0	(1.4)	[17]	0.004	1	2.46	1.30
**DDAH1**	O94760	12.6	(1.6)	[44]	13.8	(1.3)	[18]	0.003	1	2.29	1.20
**EEF1A1**	P68104	13.6	(1.1)	[21]	14.7	(1.5)	[18]	0.013	1	2.18	1.12
**hCG_2039566**	A0A0U1RR32	13.5	(1.7)	[12]	15.4	(1.5)	[11]	0.01	1	3.74	1.90
**LTF**	E7EQB2	15.2	(1.2)	[58]	16.4	(1.9)	[25]	0.006	1	2.33	1.22
**PCSK1**	P29120	13.2	(2.3)	[44]	14.7	(1.8)	[15]	0.015	1	2.82	1.50
